# Quantitative Measurement of Cooperativity in H-Bonded Networks

**DOI:** 10.1021/jacs.2c08120

**Published:** 2022-10-12

**Authors:** Lucia Trevisan, Andrew D. Bond, Christopher A. Hunter

**Affiliations:** Yusuf Hamied Department of Chemistry, University of Cambridge, Lensfield Road, CambridgeCB2 1 EW, U.K.

## Abstract

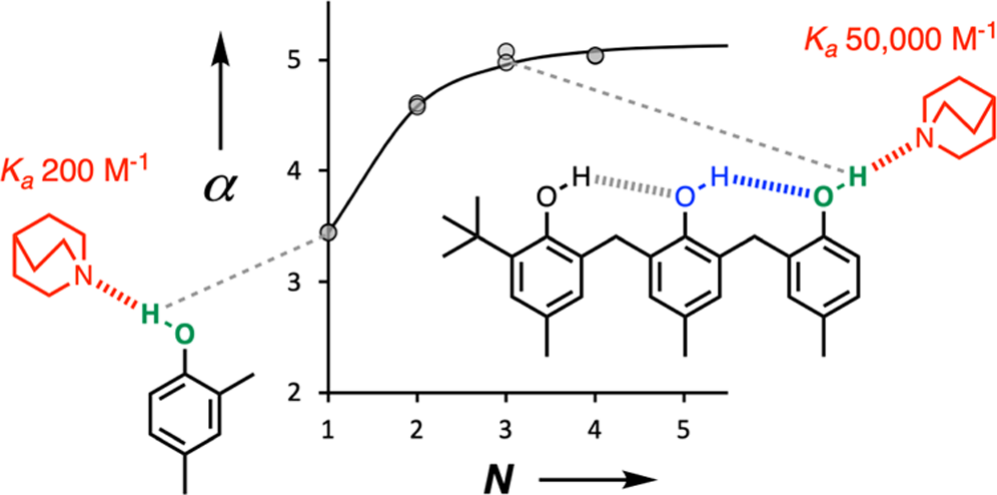

Cooperative H-bonding interactions are a feature of supramolecular networks involving alcohols. A family of phenol oligomers, in which the hydroxyl groups form intramolecular H-bonds, was used to investigate this phenomenon. Chains of intramolecular H-bonds were characterized using nuclear magnetic resonance (NMR) spectroscopy in solution and X-ray crystallography in the solid state. The phenol oligomers were used to make quantitative measurements of the effects of the intramolecular interactions on the strengths of intermolecular H-bonding interactions between the H-bond donor on the end of the chain and a series of H-bond acceptors. Intramolecular H-bonding interactions in the chain increase the strength of a single intermolecular H-bond between the terminal phenol and quinuclidine by up to 14 kJ mol^–1^ in the *n*-octane solution. Although the magnitude of the effect increases with the length of the H-bonded chain, the first intramolecular H-bond has a much larger effect than subsequent interactions. H-bond cooperativity is dominated by pairwise interactions between nearest neighbors, and longer range effects are negligible. The results were used to develop a simple model for cooperativity in H-bond networks using an empirical parameter κ to quantify the sensitivity of the H-bond properties of a functional group to polarization. The value of κ measured in these systems was 0.33, which means that formation of the first H-bond increases the polarity of the next H-bond donor in the chain by 33%. The cumulative cooperative effect in longer H-bonded chains reaches an asymptotic value, which corresponds to a maximum increase in the polarity of the terminal H-bond donor of 50%.

## Introduction

H-bonding is one of the most important noncovalent interactions in supramolecular chemistry.^1^ H-bonds are involved in molecular recognition, protein folding,^2^ DNA duplex formation,^3^ and catalysis.^4,5^ There is evidence that H-bonds become stronger upon the formation of a network, suggesting that the interaction energies in complex systems are nonadditive.^6−10^ H-bond cooperativity in water networks was first postulated in 1957,^11^ but it took more than 10 years for experimental evidence to appear for positive cooperativity in H-bonding interactions involving hydroxyl groups. Infrared studies indicate that the formation of a H-bond between an alcohol and a H-bond acceptor increases the strength of the H-bonding interaction with a second hydroxyl group.^12−15^ These cooperative effects have important consequences for the solvation properties of alcohols because the presence of self-associated H-bonded networks means that alcohols are significantly more polar solvents than the H-bonding properties of monomeric alcohols would suggest.^16−18^

Despite many theoretical studies,^7−10^ experimental quantification of the magnitude of cooperative effects on the free energy changes associated with the formation of H-bond networks has proved elusive. Synthetic molecular torsion balances have been used to measure intramolecular H-bonding interactions between a formamide H-bond acceptor and a series of phenol H-bond donors.^19^ Catechol, which has an intramolecular hydroxyl–hydroxyl H-bond, was found to make a significantly stronger H-bond with the amide group than a simple phenol. However, the two hydroxyl groups in catechol are directly conjugated, so it is difficult to disentangle the contributions due to polarization *via* the covalent bonding framework from cooperative effects due to the intramolecular H-bond. Here, we describe a new approach to direct measurement of cooperativity in H-bonded networks and show that cooperative effects due to H-bonding interactions between hydroxyl groups can increase the strength of a single intermolecular H-bonding interaction by up to 14 kJ mol^–1^.

## Approach

**Figure 1:**
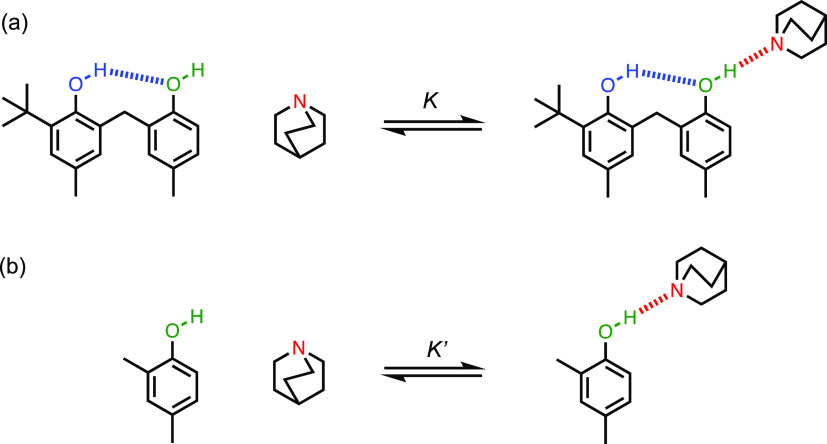
Quantification of cooperative effects on an intermolecular phenol·quinuclidine H-bond. (a) Interaction of a H-bonded phenol with quinuclidine. (b) Reference interaction of a non-H-bonded phenol with quinuclidine.

The approach is shown in Figure 1. Figure 1a shows a bisphenol, which makes an intramolecular hydroxyl–hydroxyl H-bonding interaction that alters the properties of the green phenol H-bond donor. The association constant (*K*) measures the intermolecular H-bonding interaction of the green hydroxyl group with quinuclidine. Figure 1b shows the corresponding equilibrium for the interaction of quinuclidine with a reference phenol, which does not have an intramolecular H-bond (*K*′). The effect of the blue intramolecular H-bond on the H-bond donor properties of the green phenol can be quantified by measuring the ratio of the two association constants (*K*/*K*′).

Three important features of this particular system simplify the analysis of the results: (1) the two phenol groups are separated by a methylene group, which prevents any through bond polarization *via* the covalent framework; (2) the *t*-butyl group sterically inhibits intermolecular H-bonding interactions with the second phenol group;^20^ (3) the use of a nitrogen H-bond acceptor removes any ambiguity in the structure of the complex (if an oxygen H-bond acceptor is used, the intramolecular H-bond can be broken and replaced by a second intermolecular interaction with the oxygen acceptor).^19^

**Figure 2:**
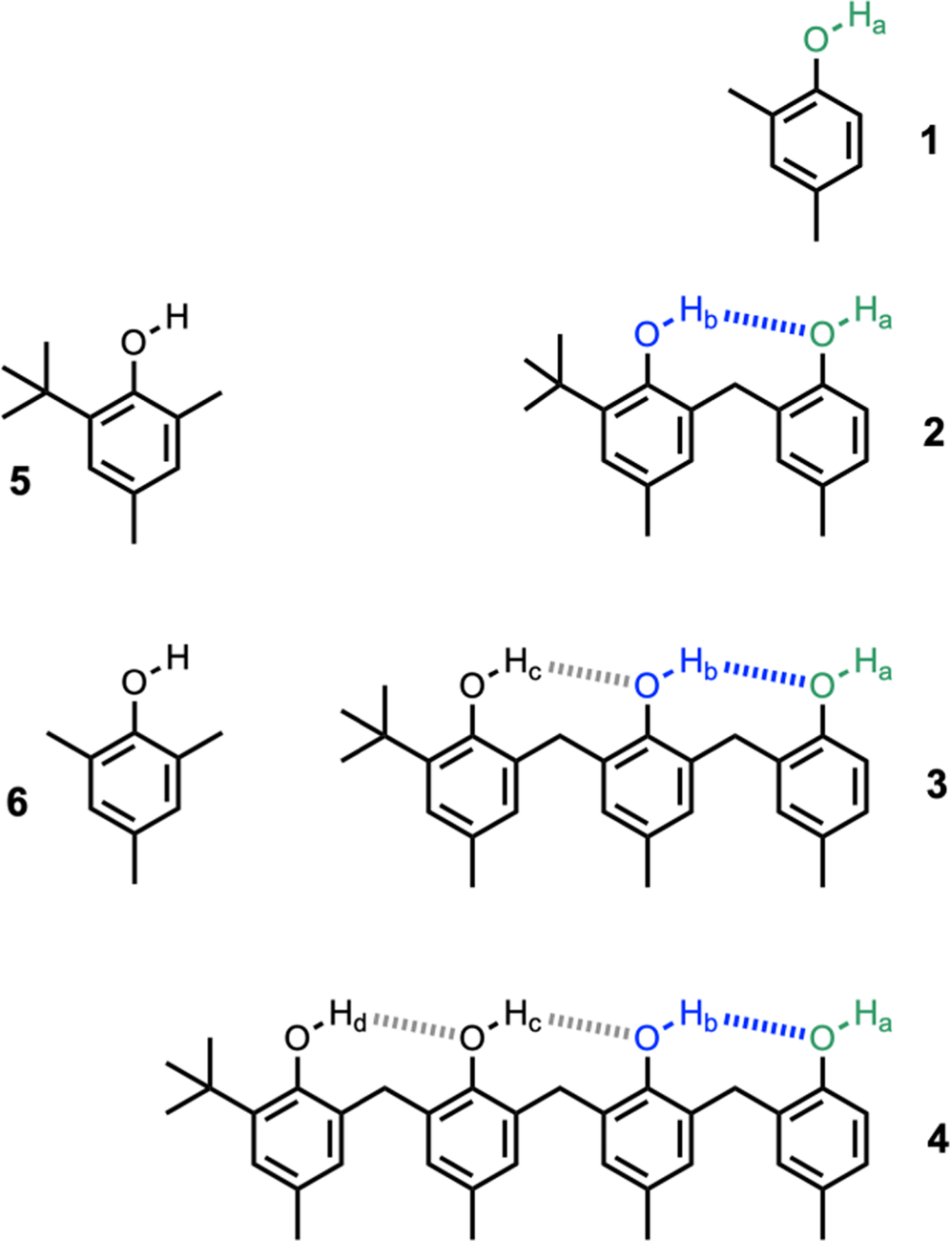
Chemical structures of phenols **1**-**6**. The ^1^H nuclear magnetic resonance (NMR) labeling scheme for the hydroxyl groups is shown.

This approach can be extended to longer H-bonded chains using compounds **3** and **4** (Figure 2). Compounds **5** and **6** in Figure 2 serve as reference molecules that will allow us to rule out alternative H-bonding modes by quantifying the strengths of possible intermolecular H-bonding interactions with H-bond donor sites that have substitution patterns that correspond to the phenol groups in the middle of the H-bonded chains.

## Results and Discussion

### Synthesis

Compounds **2**, **3**, and **4** were synthesized using procedures based on the literature (see Figure S1).^20−22^ Compounds **1**, **5**, and **6** were commercially available.

### Intramolecular H-Bonding Interactions

**Figure 3:**
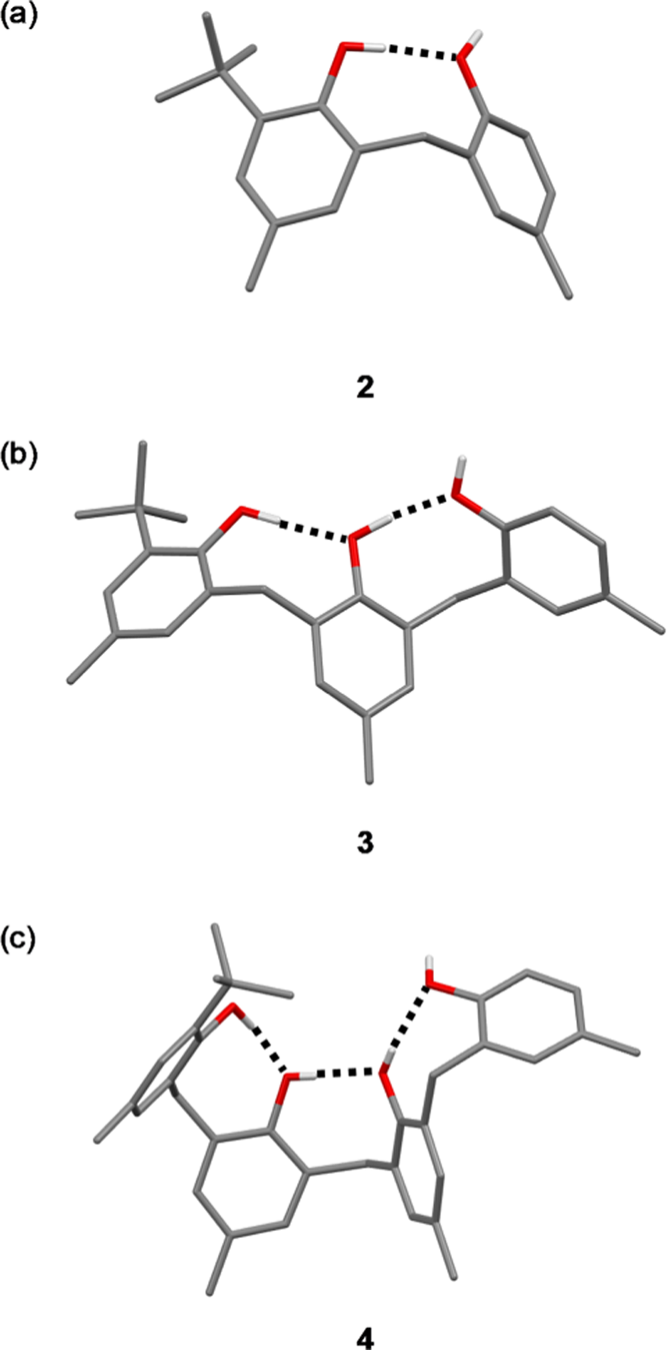
Molecular structures of (a) **2**, (b) **3**, and (c) **4**(20) taken from X-ray crystal structures. Intramolecular H-bonding interactions are shown as dotted lines. The terminal hydroxyl group of **3** forms an intermolecular H-bond to a molecule of acetonitrile present in the crystal (see below).

The X-ray crystal structures of **2**, **3·**MeCN and **4** were obtained and confirmed the anticipated presence of the network of intramolecular H-bonds in the solid state (Figure 3).^20^ In all cases, the phenol with the *ortho t*-butyl group acts as an intramolecular H-bond donor, and the phenol at the other end of the chain acts as an intramolecular H-bond acceptor. Thus, there is only one H-bond donor site, which does not make an intramolecular interaction: the phenol at the end of the chain of intramolecular H-bonds.

NMR spectroscopy was used to confirm that the conformations observed in the solid state persist in solution. It was possible to assign all of the signals in the ^1^H NMR spectra of compounds **2**–**4** in deuterochloroform using a combination of two-dimensional (2D) experiments: COSY, HSQC, and HMBC (see Figures S2–S16). The signals due to the nonequivalent phenol hydroxyl groups were clearly resolved and could be individually assigned. Differences in the chemical shift between different phenol hydroxyl groups indicate the extent to which they are involved in intramolecular H-bonding interactions. For example, for compound **2**, the hydroxyl donor that is involved in an intramolecular H-bond in the X-ray crystal structure appears at 6.5 ppm, and the hydroxyl donor that does not form an intramolecular H-bond in the X-ray crystal structure appears at 5.5 ppm (see Figure S2). The difference of 1 ppm in the chemical shift suggests that the intramolecular H-bond observed in the solid state is also present in solution.^23,24^ Similar results were obtained for compounds **3** and **4** (Figures S7–S16), confirming that the chains of intramolecular H-bonding interactions shown in Figure 3 persist in deuterochloroform solution.

**Figure 4:**
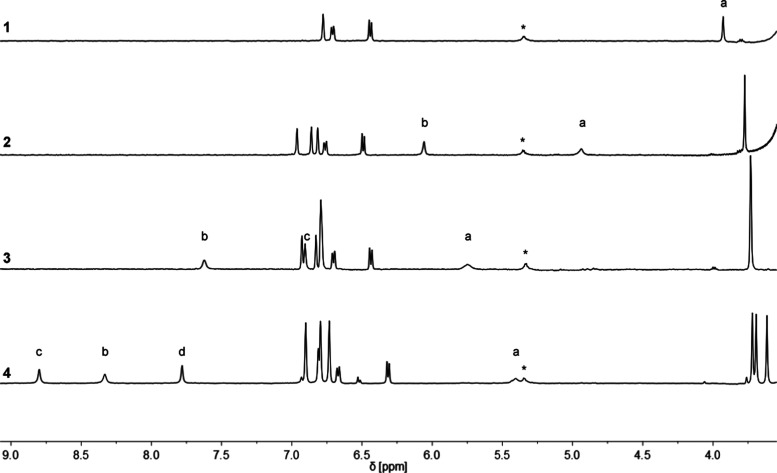
Partial 500 MHz ^1^H NMR spectra of 0.24 mM solutions of **1**, **2**, **3**, and **4** recorded at 298 K in *n*-octane with WET solvent suppression. Signals due to hydroxyl protons are labeled (see Figure 2 for the labeling scheme), and the asterisk indicates an impurity present in the solvent.

^1^H NMR spectra of compounds **1**–**4** were also recorded in *n*-octane using WET solvent suppression,^25^ and the spectra were very similar to those recorded in deuterochloroform, allowing direct assignment of the signals. Figure 4 shows the region of the spectra where the phenol hydroxyl groups appear. In each case, the signal due to the hydroxyl donor that is not involved in an intramolecular H-bond (a) appears at the lowest chemical shift (<6 ppm), and the chemical shifts of the signals due to the hydroxyl donors that are involved in intramolecular H-bonds are up to 5 ppm higher. As the length of the H-bonded chain increases, the chemical shifts of the hydroxyl donors involved in intramolecular H-bonds increase from 6 to 9 ppm.^26^ In addition to increasing the chemical shift of the hydroxyl group that acts as a donor, the formation of an intramolecular H-bond causes a smaller increase in the chemical shift of the hydroxyl group that acts as an acceptor. For example, comparing compound **2** with compound **1**, the chemical shift of the hydroxyl group that acts as the donor in the intramolecular H-bond in compound **2** increases by 2 ppm (b), and the chemical shift of the hydroxyl group that acts as the acceptor increases by 1 ppm (a). Thus, the hydroxyl groups in the middle of the H-bonded chain in compound **4** (signals b and c) show significantly larger increases in the chemical shift than either the donor or the acceptor on the ends of the chain (signals a and d).

### Intermolecular H-Bonding Interactions

**Figure 5:**
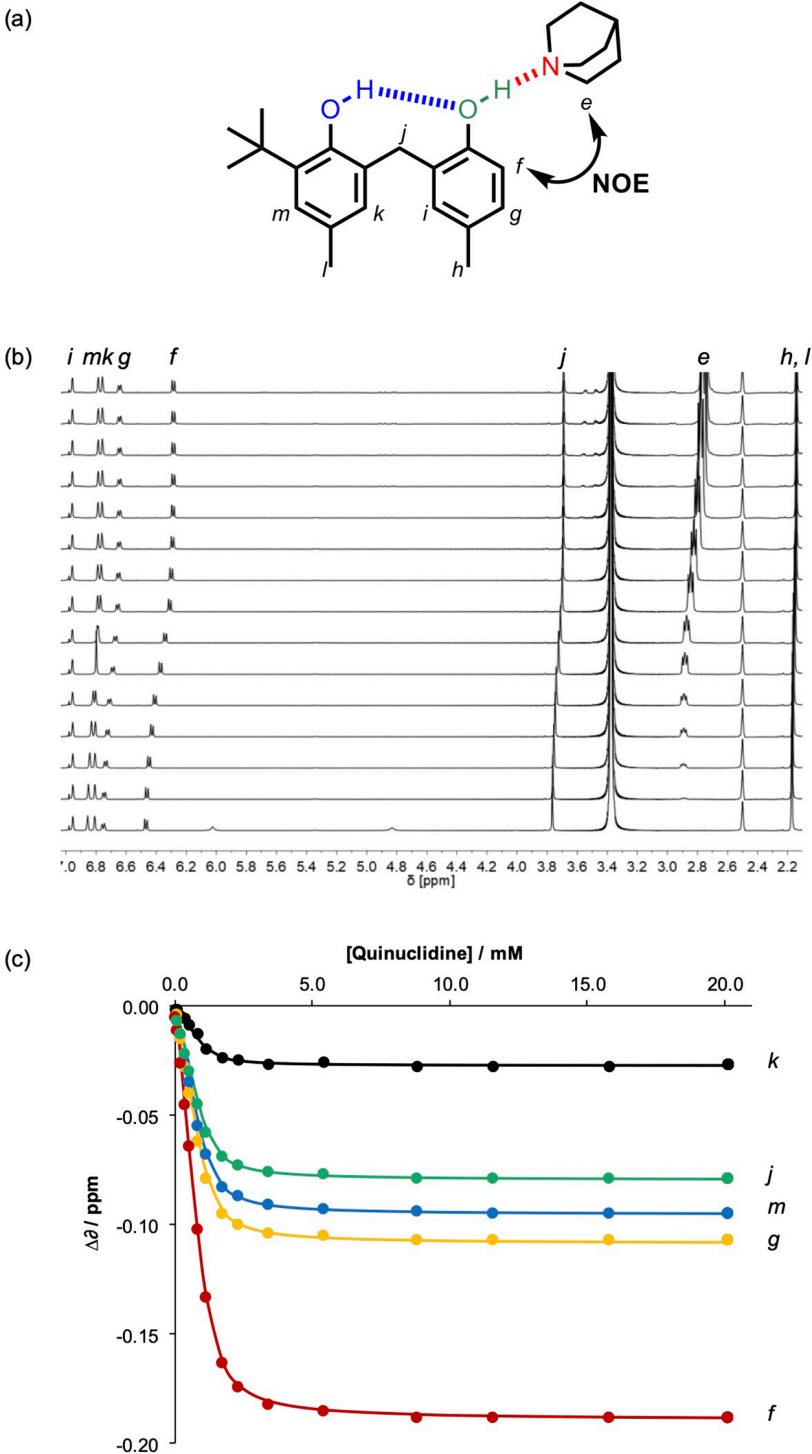
^1^H NMR titration of quinuclidine into a 1.22 mM solution of **2** in *n*-octane at 298 K. (a) Structure of the 1:1 complex showing the proton labeling scheme. The intermolecular NOE observed in a NOESY spectrum of a 1:1 mixture of quinuclidine and **2** is indicated. (b) 500 MHz ^1^H-NMR spectra recorded with WET solvent suppression. (c) Lines of best fit of the ^1^H-NMR data (points) to a 1:1 binding isotherm.

The formation of intermolecular H-bonds with quinuclidine was investigated using ^1^H NMR spectroscopy. ^1^H-NMR dilution experiments in *n*-octane show that there is no self-association at millimolar concentrations. Figure 5 shows data from a ^1^H-NMR titration of quinuclidine into a solution of **2** in *n*-octane. The addition of quinuclidine catalyzed the chemical exchange of the hydroxyl protons, resulting in fast exchange spectra where the individual hydroxyl signals were not resolved and could not be separately monitored in titrations. Figure 5c shows that the changes in the chemical shift observed for all of the signals due to CH protons fit well to a 1:1 binding isotherm. One signal showed a much larger change in the chemical shift than any of the other signals. This signal is due to proton f, which is *ortho* to the only phenol H-bond donor not involved in an intramolecular H-bond, suggesting that quinuclidine binds to this phenol group. A NOESY spectrum provided further evidence for this interaction: Figure 5a illustrates the intermolecular NOE that was observed between proton f and the quinuclidine methylene group in a NOESY spectrum of a 1:1 mixture of quinuclidine and **2**. NOESY spectra recorded for 1:1 mixtures of **1**, **3**, or **4** and quinuclidine all showed the corresponding NOE, that is, a cross-peak between the signal due to the aromatic CH proton *ortho* to the terminal phenol hydroxyl group and the signal due to the quinuclidine methylene group (see Figures S41–S44).^27,28^ These observations indicate that quinuclidine binds to the end of the chain of intramolecular H-bonded phenols in all cases.

**Figure 6:**
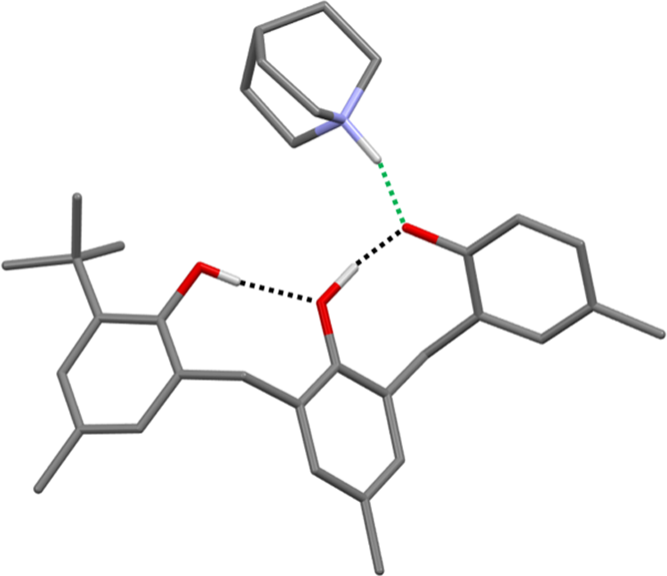
X-ray crystal structure of the **3**·quinuclidine complex. The location of the proton on the quinuclidine nitrogen was deduced from the X-ray data and supported by DFT-D calculations.

When quinuclidine was added to **3** in chloroform solution, a 1:1 complex precipitated. The X-ray crystal structure of this complex is shown in Figure 6. The chain of intramolecular H-bonds observed in the X-ray crystal structure of **3** (Figure 3b) is also present in the complex, and quinuclidine is H-bonded to the terminal phenol. The positions of protons H_a_ and H_c_ were clearly visible from the X-ray data, with the latter transferred to quinuclidine to form a salt in the solid state. The position of H_b_ was less clearly defined, with the electron density appearing to be distributed between the central and terminal oxygen atoms. Periodic dispersion-corrected density functional theory (DFT-D) calculations support that the complex exists as a salt in the solid state and suggest that the minimum-energy location of H_b_ is indeed close to the midpoint of the central and terminal oxygen atoms (see SI for details).

### H-Bond Donor Parameters

The H-bond donor properties of compounds **1**–**4** were determined by measuring the association constants for the formation of 1:1 complexes with three different H-bond acceptors, quinuclidine (Quin), *n*-heptylamine (HeptNH_2_), and tri-*n*-octylamine (Oct_3_N), in *n*-octane. UV–vis dilution experiments in *n*-octane show that there is no self-association of compounds **1**–**4** at the concentrations used to carry out the titrations. The addition of the H-bond acceptors to solutions of the phenols led to the appearance of a blue-shifted band in the UV–vis absorption spectra, which is characteristic of the formation of a H-bonded complex (see SI). The UV–vis absorption titration data fit well to a 1:1 binding isotherm in all cases, and the association constants are reported in Table 1. The presence of intramolecular H-bonding interactions in compounds **2**–**4** leads to large increases in the association constant compared with **1**. Compounds **5** and **6** are simple phenols, which have the same substitution pattern as the phenol units in the middle of the chains of intramolecular H-bonds in compounds **2**–**4**. The association constants determined for the formation of 1:1 complexes between quinuclidine and compounds **5** and **6** are an order of magnitude lower than the corresponding values measured for compound **1** (see Table S2). This result supports the conclusion that H-bond acceptors interact with the terminal phenol donor in compounds **2**–**4** and not with the other phenol units, which are involved in intramolecular H-bonds and are intrinsically weaker H-bond donors.

**Table 1:** Association Constants (M^–1^) for the Formation of 1:1 Complexes Measured by UV–Vis Absorption Titrations in *n*-Octane at 298 K^a^

	Acceptor		
Donor	Quin	HeptNH_2_	Oct_3_N
**1**	(1.8 ± 0.1) × 10^2^	46 ± 9	<5
**2**	(9.1 ± 0.3) × 10^3^	(1.1 ± 0.1) × 10^3^	(3.3 ± 0.1) × 10^2^
**3**	(4.5 ± 0.5) × 10^4^	(3.3 ± 0.2) × 10^3^	(6.7 ± 0.3) × 10^2^
**4**	(3.9 ± 0.5) × 10^4^	(3.9 ± 0.2) × 10^3^	(7.6 ± 0.2) × 10^2^

a Errors are the standard error of the mean of three independent experiments.

**Figure 7:**
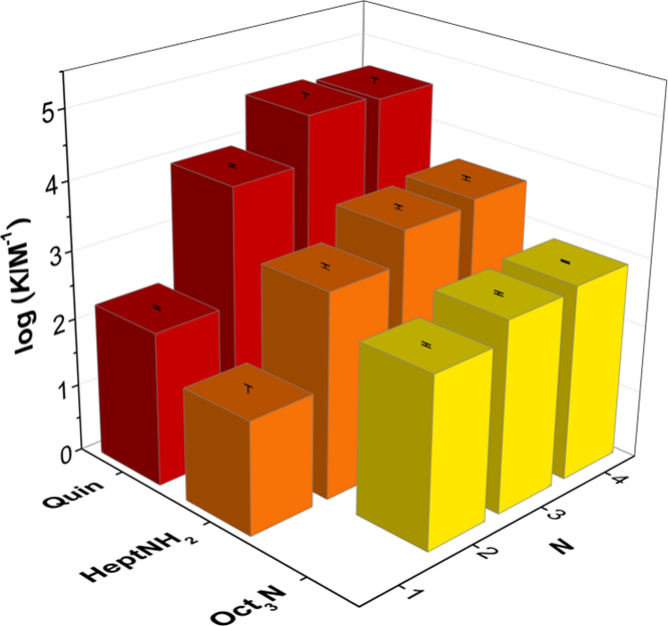
Association constants for the formation of 1:1 complexes, log(*K*/M^–1^), plotted as a function of the number of phenols present in the H-bonded chain (*N*).

Figure 7 shows the relationship between the association constant and the number of phenol units in the H-bonded chain (*N*). The presence of a single intramolecular H-bond in **2** increases the association constant for the complex formed with quinuclidine by two orders of magnitude compared with compound **1**. Cooperative effects in compound **2** increase the strength of the intermolecular H-bond by 10 kJ mol^–1^. The addition of the second intramolecular H-bond in **3** leads to a further increase in the association constant, but the increase in H-bond strength is smaller (4 kJ mol^–1^). The addition of the third intramolecular H-bond in **4** does not result in any further changes.

The association constant for the formation of a H-bonded complex can be written in terms of the H-bond parameters for the H-bond donor, α, the H-bond acceptor, β, and the solvent, α_S_ and β_S_ (eq 1).^29^
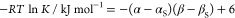
1Using literature values for the solvent parameters (α_S_ = 1.2, β_S_ = 0.6)^30^ and the H-bond acceptors (β = 9.0 for Quin,^31,32^ β = 7.5 for HeptNH_2_,^33,34^ β = 6.8 for Oct_3_N^33,34^), it is possible to use the experimental values of the association constants in Table 1 to determine the H-bond donor parameter α for each of compounds **1**–**4** (eq 2).
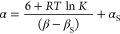
2

The results are shown in Table 2. The values of *α* determined using different H-bond acceptors are consistent, which shows that eq 1 provides an accurate description of the behavior of these systems. The presence of the intramolecular H-bond in compound **2** causes a large increase in the H-bond donor parameter compared with compound **1**. The second H-bond in the chain in compound **3** causes a smaller increase in α, and the third H-bond in the chain in compound **4** has minimal impact. Theoretical values of H-bond donor parameters can be obtained from the maximum in the molecular electrostatic potential calculated on the van der Waals surface using *ab initio* methods (see SI pages S71–S72).^35^ The calculated values of α for compounds **1**–**4** are listed in Table 2 and agree well with the experimental results. Thus, gas phase *ab initio* calculations of molecular electrostatic potential appear to provide an accurate description of the effects of H-bond cooperativity on the free energies of solution phase interactions.

**Table 2:** H-Bond Donor Parameters (α)

		Experiment		
		Acceptor		
Donor	Calculated	Quin	HeptNH_2_	Oct_3_N
**1**	3.7	3.5	3.5	
**2**	4.7	4.6	4.6	4.5
**3**	5.0	5.1	5.0	4.8
**4**	5.1	5.0	5.1	4.8

**Figure 8:**
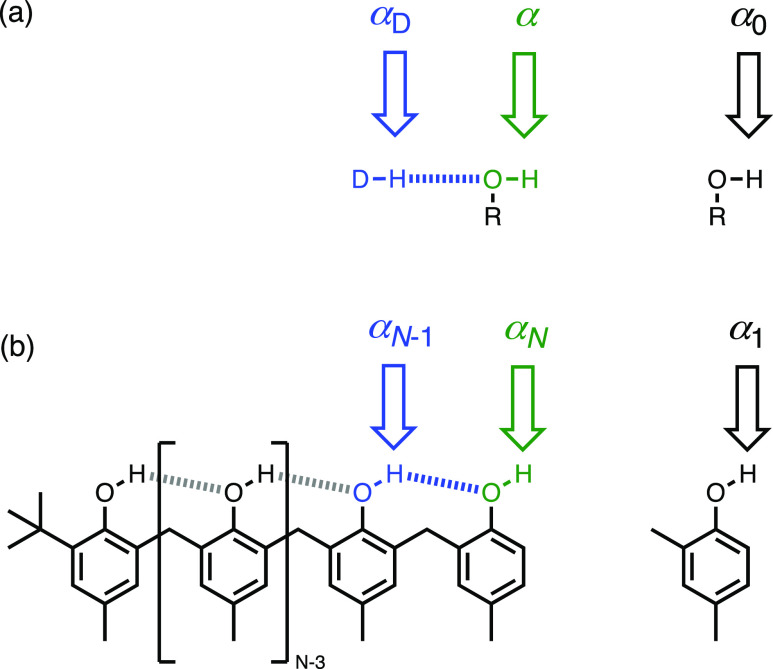
(a) When a hydroxyl group interacts with a H-bond donor (DH), the H-bond donor parameter (α) increases relative to a non-H-bonded hydroxyl group (α_0_). (b) H-bond donor parameter of the green phenol on the end of a H-bonded chain (α_*N*_) is increased relative to a non-H-bonded phenol (α_1_) by an amount that depends on the polarity of the phenol that makes the blue intramolecular H-bond (α_*N*–1_).

The effects of H-bond cooperativity clearly attenuate with the length of the H-bonded chain. A simple model that accounts for this observation is to assume that the magnitude of the cooperative effect depends on the polarity of the H-bond donor that makes the intramolecular H-bond. The idea is illustrated in Figure 8a. A free hydroxyl group has a H-bond donor parameter α_0_, but the interaction with a second H-bond donor DH, which has a H-bond donor parameter α_D_, leads to an increase in the polarity of the hydroxyl donor. The H-bond donor parameter of the H-bonded hydroxyl group, α, is given by eq 3, where κ is a functional-group-specific parameter that quantifies the sensitivity to cooperative effects.
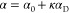
3Figure 8b shows how this idea translates to the H-bonded chains of phenol units investigated here. The H-bond parameter α_*N*_ describes the polarity of the phenol unit on the end of a H-bonded chain of *N* phenols. The H-bond parameter α_*N*–1_ describes the polarity of the intramolecular donor (*cf.* α_D_ in Figure 8a). The H-bond parameter α_1_ describes the polarity of a free phenol, that is a chain length of one. Thus, applying eq 3 to the phenol oligomers shown in Figure 8b gives eq 4.
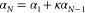
4The H-bond donor properties of the intramolecular donor that causes the cooperative effects can be described in the same way by considering this group as the phenol unit on the end of a H-bonded chain of *N* – 1 phenols (eq 5).
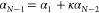
5Assuming that each of the phenol units in the H-bonded chain would have the same H-bond donor parameter when they do not make intramolecular H-bonds, that is, α_0_, then eqs 4 and 5 can be generalized to describe the H-bond donor properties of the end of a chain of any length in terms of just two parameters (eq 6).
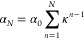
6

**Figure 9:**
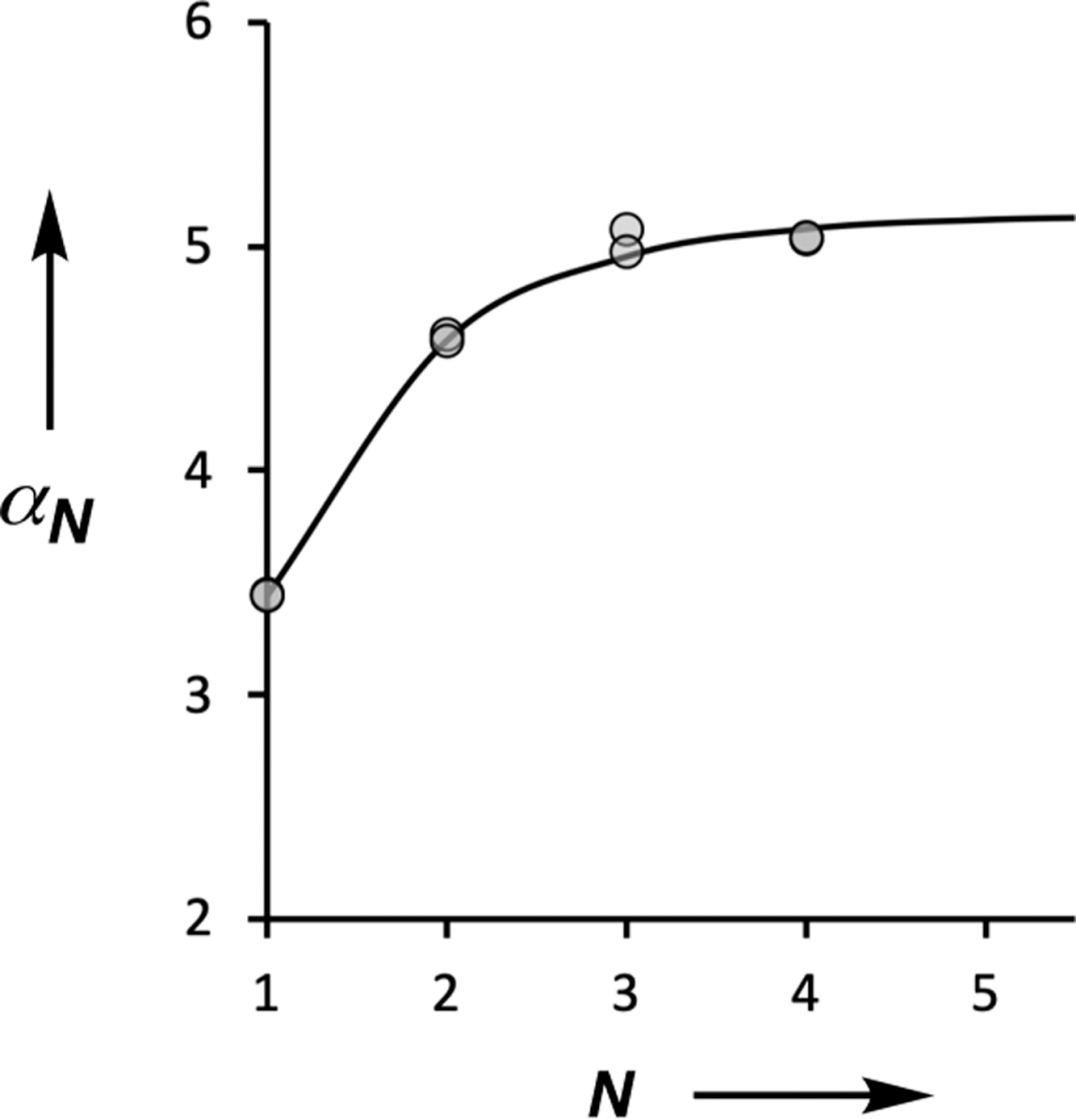
Relationship between the H-bond donor parameter and the number of phenol units in the H-bonded chain (*N*). The experimental values determined using quinuclidine and *n*-heptylamine are shown as points, and the line corresponds to eq 6 with α_0_ = 3.5 and κ = 0.33.

Figure 9 shows that the experimental data obtained for compounds **1**–**4** are described accurately by eq 6 using a value of α_0_ = 3.5 and κ = 0.33. In other words, the effects of cooperativity on the H-bonding properties of a network can be understood based on the sum of nearest-neighbor pairwise interactions, and longer range multibody effects are negligible. In the limit of an infinite chain, the series in eq 6 simplifies to eq 7. Using κ = 0.33 in eq 7 implies that the maximum increase in H-bond donor strength that will be observed at the end of a long chain of H-bonded phenols is 50%. The value of α_∞_ is 5.2, which is effectively reached in compound **3** (α = 5.1) when the chain length is only three phenol units long.
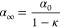
7

The intramolecular H-bonding interactions investigated here all involve an eight-membered ring containing a conformationally flexible methylene group. It is unlikely that conformational entropy makes a contribution to the measured association constants because there is no change in conformation or in conformational flexibility on binding, and the same intramolecular H-bonding network is present in both the free and bound states. However, it is possible that the geometry of the intramolecular H-bonds affects the magnitude of the cooperative effects observed. The eight-membered ring allows the H-bonds to attain an optimal alignment of the hydroxyl groups with OH···O bond angles close to 180°. Studies of H-bonded systems with different ring sizes and bond angles would be required to establish the significance of this parameter.

**Figure 10:**
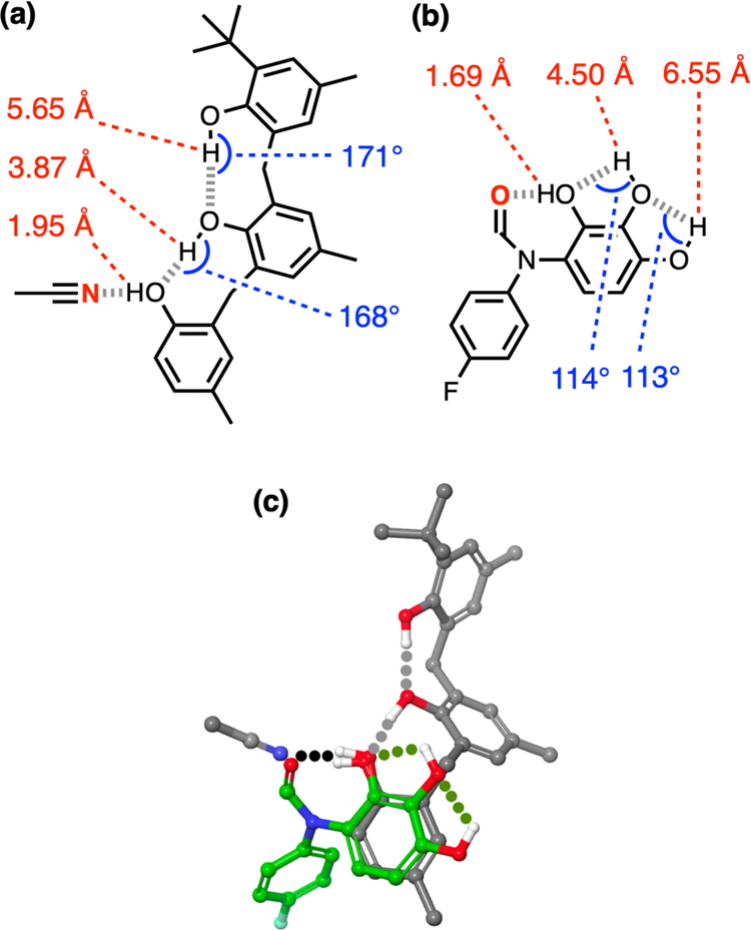
Comparison of the geometry of the H-bonded network in **3**·MeCN with the Cockroft torsion balance. (a) Distances between the acetonitrile nitrogen atom and H-bond donor protons (red), and OH···O bond angles for intramolecular H-bonds (blue) in the X-ray crystal structure of **3**·MeCN. (b) Distances between the formamide oxygen atom and H-bond donor protons (red), and OH···O bond angles for intramolecular H-bonds (blue) in the DFT structure of a torsion balance (B3LYP-D3 6-31G*). (c) Overlay of the three-dimensional structures obtained by aligning the C-OH···N and C-OH···O atoms involved in the H-bond indicated in black. Intramolecular H-bonds between hydroxyl groups are highlighted in gray for **3**·MeCN and green for the torsion balance.

There are two different factors that contribute to H-bond cooperativity in these systems, bond polarization, and secondary electrostatic interactions. H-bonding polarizes the electron density in an OH bond, increasing the size of the effective positive charge on the hydroxyl proton and the dipole associated with the OH bond.^36^*Ab initio* calculations indicate that the Mulliken charge on the terminal hydroxyl proton increases from 0.339 for compound **1** to 0.352 for compound **2**, 0.354 for compound **3**, and 0.357 for compound **4** (see SI pages S72). In addition to the primary interaction between the H-bond acceptor and the terminal phenol group, direct long-range secondary electrostatic interactions with the hydroxyl protons of the other phenol groups further along the H-bonded chain can stabilize the complexes. Figure 10a shows the distances between the phenol protons and the nitrogen atom of the H-bonded acetonitrile molecule in the X-ray crystal structure of **3**·MeCN. The second hydroxyl group in the chain is twice as far away (3.87 Å) as the H-bonded proton (1.95 Å) but close enough to be considered a contact with the acetonitrile, and the third hydroxyl proton is much further away (5.65 Å). In multiple H-bonded donor-acceptor arrays, each attractive secondary electrostatic interaction contributes a factor of about 3 to the stability of the complex, but the distances involved are considerably shorter (3.0 Å).^37−39^ The contribution due to secondary electrostatic interactions is therefore unlikely to account for the 250-fold increase in the stability of the **3**·Quin complex compared with the **1**·Quin complex, and bond polarization must play a major role.

Comparison of the cooperativity observed in compounds **1**–**4** with the torsion balances reported by Cockroft provides further insight.^19^ There are important differences between the two systems because the H-bonds measured with the torsion balances involve a different H-bond acceptor, different solvent, and different substituents on the phenol groups, and the geometries of the intramolecular interactions between the hydroxyl groups are different (see Figure 10). However, in both cases, the strength of the interaction with the terminal H-bond donor increases significantly when it is involved in an intramolecular H-bond with a second hydroxyl group: the **2**·Quin complex is 50 times more stable than the **1**·Quin complex in *n*-octane; in the torsion balances, the formamide·catechol H-bond is five times more stable than the formamide·phenol H-bond in chloroform. An overlay of the three-dimensional structures of the **3**·MeCN complex and the pyrogallol torsion balance (Figure 10c) shows that the geometry of the H-bond network is quite different in the two systems. The fact that both types of networks lead to substantial positive cooperativity suggests that H-bond cooperativity is not a sensitive function of the precise arrangement of the H-bonded groups. The torsion balance geometry illustrated in Figure 10b reinforces the idea that polarization of the OH bond, and not secondary electrostatic interactions, is the major source of cooperativity in these systems because the distances between the formamide oxygen atom and the second and third phenol protons in the H-bonded chain are even longer than in the **3**·MeCN complex as shown in Figure 10a.

The formamide·pyrogallol H-bond in the torsion balance shown in Figure 10b is actually less stable than the corresponding formamide·catechol H-bond in chloroform. In contrast, the presence of the third hydroxyl group in the H-bonded chain stabilizes the **3**·Quin complex by a factor of five times compared with the **2**·Quin complex in *n*-octane. There are two possible reasons for this discrepancy. First, the three hydroxyl groups in the torsion balance are conjugated through the same aromatic ring, and the H-bond donor properties of the terminal hydroxyl group in the H-bonded chain are perturbed by through-bond polarization effects as well as by through-space effects due to intramolecular H-bonding. Second, the geometries of the intramolecular H-bonds in the torsion balance are suboptimal with OH···O bond angles of 113–114° (Figure 10b) compared with the almost linear geometries in compound **3** (168–171° in Figure 10a). These two factors are difficult to disentangle but appear to suppress cooperative effects in the pyrogallol torsion balance.

## Conclusions

In conclusion, we have developed a supramolecular approach to measuring cooperativity in H-bonded networks. The presence of chains of intramolecular H-bonding interactions between hydroxyl groups in a family of phenol oligomers was established through NMR spectroscopy and X-ray crystallography. These intramolecular interactions persist when a 1:1 complex is formed with a H-bond acceptor in *n*-octane, and there is a clear relationship between the stability of this complex and the number of intramolecular H-bonding interactions. The presence of intramolecular H-bonds between hydroxyl groups increases the H-bond donor strength of the terminal phenol donor in the chain, resulting in an increase of up to 14 kJ mol^–1^ in the strength of a single intermolecular H-bond formed with a H-bond acceptor. H-bond donor parameters α were determined for the phenol oligomers using the experimental association constants for complexation with three different amine acceptors. The values increase from 3.5 for a simple phenol to 5.0 for a donor on the end of a chain of four H-bonded phenol units. These results are consistent with theoretical values obtained from molecular electrostatic potential surfaces calculated in the gas phase using *ab initio* methods. Intramolecular H-bonding interactions could increase the polarity of the terminal H-bond donor by secondary electrostatic interactions, but the results indicate that polarization of the OH bond plays a major role.

There is a large increase in H-bond donor strength associated with the formation of the first intramolecular H-bond in the chain, but the increase in H-bond donor strength becomes lower as the number of intramolecular H-bonds in the chain grows. The results can be explained by a model that assumes that cooperativity is dominated by pairwise interactions between nearest neighbors in the chain. We propose a parameter κ, which quantifies the sensitivity of the H-bond properties of a specific functional group to cooperative effects. For the phenol hydroxyl group, the value of κ is 0.33, which means that the formation of a H-bond with a donor with an α value of 3.0 increases the H-bond donor strength of the phenol by 1.0 (=0.33 × 3.0). This approach can be extended to other systems, and we are collecting experimental data to determine the values of κ for a range of different functional groups.

## Supporting Information Available

Materials and methods; detailed synthetic procedures; full characterization including ^1^H and ^13^C NMR spectra of all compounds; and details of X-ray crystallography, NMR experiments, UV–vis absorption titrations, and *ab initio* calculation of H-bond parameters are available in the Supporting Material (PDF)

## References

[ref1] Martin D. L.; Rossotti F. J. C. In The Hydrogen-Bonding of Monocarboxylates in Aqueous Solution, Proceedings of the Chemical Society of London 2; Royal Society Chemistry, 1959; pp 29–72.

[ref2] Pauling L.; Corey R. B.; Branson H. R. The structure of proteins: Two hydrogen-bonded helical configurations of the polypeptide chain. Proc. Natl. Acad. Sci. U.S.A. 1951, 37, 205–211. 10.1073/pnas.37.4.205. 14816373 PMC1063337

[ref3] Watson J. D.; Crick F. H. C. Molecular Structure of Nucleic Acids: A Structure for Deoxyribose Nucleic Acid. Nature 1953, 171, 737–738. 10.1038/171737a0. 13054692

[ref4] Putnam C. D.; Arvai A. S.; Bourne Y.; Tainer J. A. Active and inhibited human catalase structures: ligand and NADPH binding and catalytic mechanism. J. Mol. Biol. 2000, 296, 295–309. 10.1006/jmbi.1999.3458. 10656833

[ref5] Taylor M. S.; Jacobsen E. N. Asymmetric Catalysis by Chiral Hydrogen-Bond Donors. Angew. Chem., Int. Ed. 2006, 45, 1520–1543. 10.1002/anie.200503132. 16491487

[ref6] Kariuki B. M.; Harris K. D. M.; Philp D.; Robinson J. M. A. A Triphenylphosphine Oxide-Water Aggregate Facilitates an Exceptionally Short C-H···O Hydrogen Bond. J. Am. Chem. Soc. 1997, 119, 12679–12680. 10.1021/ja973506z.

[ref7] Hermansson K.; Alfredsson M. Molecular polarization in water chains. J. Chem. Phys. 1999, 111, 1993–2000. 10.1063/1.479468.

[ref8] Kollman P. A General Analysis of Noncovalent Intermolecular Interactions. J. Am. Chem. Soc. 1977, 99, 4875–4894. 10.1021/ja00457a002.

[ref9] Del Bene J.; Pople J. A. Intermolecular Energies of Small Water Polymers. Chem. Phys. Lett. 1969, 4, 426–428. 10.1016/0009-2614(69)85004-9.

[ref10] Clementi E. Determination of Liquid Water Structure. In Lecture Notes in Chemistry; Springer Verlag: Berlin/Heidelberg, 1976; 2.

[ref11] Frank H. S.; Wen W.-Y. Ion-solvent interaction. Structural aspects of ion-solvent interaction in aqueous solutions: a suggested picture of water structure. Discuss. Faraday Soc. 1957, 24, 133–140. 10.1039/DF9572400133.

[ref12] Kleeberg H.; Klein D.; Luck W. A. P. Quantitative Infrared Spectroscopic Investigations of Hydrogen-Bond Cooperativity. J. Phys. Chem. A 1987, 91, 3200–3203. 10.1021/j100296a019.

[ref13] Clotman D.; Van Lerberghe D.; Zeegers-Huyskens Th. Etude par spectrométrie infrarouge de la stoechiométrie des complexes phenols-triéthylamine. Spectrochim. Acta, Part A 1970, 26, 1621–1631. 10.1016/0584-8539(70)80221-5.

[ref14] Huyskens P. L. Factors Governing the Influence of a First Hydrogen Bond on the Formation of a Second One by the Same Molecule or Ion. J. Am. Chem. Soc. 1977, 99, 2578–2582. 10.1021/ja00450a028.

[ref15] Kleeberg H.; Luck W. A. P. Experimental Tests of the H-bond Cooperativity. Z. Phys. Chem. 1989, 2700, 613–625. 10.1515/zpch-1989-27072.

[ref16] Cook J. L.; Hunter C. A.; Low C. M. R.; Perez-Velasco A.; Vinter J. G. Solvents Effects on Hydrogen Bonding. Angew. Chem., Int. Ed. 2007, 46, 3706–3709. 10.1002/anie.200604966. 17415725

[ref17] Cabot R.; Hunter C. A. Molecular probes of solvation phenomena. Chem. Soc. Rev. 2012, 41, 3485–3492. 10.1039/C2CS15287H. 22382921

[ref18] Henkel S.; Misuraca M. C.; Troselj P.; Davidson J.; Hunter C. A. Polarisation effects on the solvation properties of alcohols. Chem. Sci. 2018, 9, 88–99. 10.1039/C7SC04890D. 29629077 PMC5875020

[ref19] Dominelli-Whiteley N.; Brown J. J.; Muchowska K. B.; Mati I. K.; Adam C.; Hubbard T. A.; Elmi A.; Brown A. J.; Bell I. A. W.; Cockroft S. L. Strong Short-Range Cooperativity in Hydrogen-Bond Chains. Angew. Chem., Int. Ed. 2017, 56, 7658–7662. 10.1002/anie.201703757. PMC5488241 28493462

[ref20] Paulus E.; Böhmer V. Die Kristallstruktur von Oligo[(2-hydroxy-1,3-phenylen)methylen]en. Makromol. Chem. 1984, 185, 1921–1935. 10.1002/macp.1984.021850914.

[ref21] Sartori G.; Bigi F.; Maggi R.; Porta C. Metal-template *ortho*-regioselective mono and bis-de-*tert*-butylation of poly-*tert*-butylated phenols. Tetrahedron Lett. 1994, 35, 7073–7076. 10.1016/0040-4039(94)88229-0.

[ref22] Yu S.; Wang Y.; Ma Y.; Wang L.; Zhu J.; Liu S. Structure, thermal stability, antioxidant activity and DFT studies of trisphenols and related phenols. Inorg. Chim. Acta 2017, 468, 159–170. 10.1016/j.ica.2017.07.022.

[ref23] Rohlfing C. M.; Allen L. C.; Ditchfield R. Proton chemical shift tensors in hydrogen-bonded dimers of RCOOH and ROH. J. Chem. Phys. 1983, 79, 4958–4966. 10.1063/2.445589.

[ref24] Yadav L. D. S. Organic Spectroscopy; Springer: Dordrecht, Netherlands, 2005; p 151.

[ref25] Smallcombe S. H.; Patt S. L.; Keifer P. A. WET Solvent Suppression and Its Applications to LC NMR and High-Resolution NMR Spectroscopy. J. Magn. Reson., Ser. A 1995, 117, 295–303. 10.1006/jmra.1995.0759.

[ref26] Kar T.; Scheiner S. Comparison of Cooperativity in CH···O and OH···O Hydrogen Bonds. J. Phys. Chem. A 2004, 108, 9161–9168. 10.1021/jp048546l.

[ref27] Jeener J.; Meier B. H.; Bachmann P.; Ernst R. R. Investigation of exchange processes by two-dimensional NMR spectroscopy. J. Chem. Phys. 1979, 71, 4546–4553. 10.1063/1.438208.

[ref28] Berger S. Gradient-selected NOESY – A fourfold reduction of the measurement time for the NOESY experiment. J. Magn. Reson., Ser. A 1996, 123, 119–121. 10.1006/jmra.1996.0222. 8980072

[ref29] Hunter C. A. Quantifying Intermolecular Interactions: Guidelines for the Molecular Recognition Toolbox. Angew. Chem., Int. Ed. 2004, 43, 5310–5324. 10.1002/anie.200301739. 15468180

[ref30] Cabot R.; Hunter C. A.; Varley L. M. Hydrogen bonding properties of non-polar solvents. Org. Biomol. Chem. 2010, 8, 1455–1462. 10.1039/B921003B. 20204221

[ref31] Graton J.; Besseau F.; Berthelot M.; Raczyńska E. D.; Laurence C. L’échelle pK_HB_ de basicité de liaison hydogène des amines tertiaires aliphatiques. Can. J. Chem. 2002, 80, 1375–1385. 10.1139/V02-176.

[ref32] Joris L.; Mitsky J.; Taft R. W. The Effects of Polar Aprotic Solvents on Linear Free-Energy Relationships in Hydrogen-Bonded Complex Formation. J. Am. Chem. Soc. 1972, 94, 3438–3442. 10.1021/ja00765a029.

[ref33] Abraham M. H.; Grellier P. L.; Prior D. V.; Morris J. J.; Taylor P. J. Hydrogen Bonding. Part 10. A scale of solute hydrogen-bond basicity using log K values for complexation in tetrachloromethane. J. Chem. Soc., Perkin Trans. 2 1990, 521–529. 10.1039/P29900000521.

[ref34] Clotman D.; Zeegers-Huyskens Th. Application des relations de Taft à la complexation. Spectrochim. Acta, Part A 1967, 23A, 1627–1634. 10.1016/0584-8539(67)80045-X.

[ref35] Calero C. S.; Farwer J.; Gardiner E. J.; Hunter C. A.; Mackey M.; Scuderi S.; Thompson S.; Vinter J. G. Footprinting Molecular Electrostatic Potential Surfaces for Calculation of Solvation Energies. Phys. Chem. Chem. Phys. 2013, 15, 18262–18273. 10.1039/C3CP53158A. 24064723

[ref36] Krijn M. P. C. M.; Feil D. A local density-functional study of the electron density distribution in the H_2_O dimer. J. Chem. Phys. 1988, 89, 5787–5793. 10.1063/1.455554.

[ref37] Kyogoku Y.; Lord R. C.; Rich A. An infrared study of the hydrogen-bonding specificity of hypoxanthine and other nucleic acid derivatives. Biochim. Biophys. Acta, Nucleic Acids Protein Synth. 1969, 179, 10–17. 10.1016/0005-2787(69)90116-6. 5781935

[ref38] Kyogoku Y.; Lord R. C.; Rich A. The effect of substituents on the hydrogen bonding of adenine and uracil derivatives. Proc. Natl. Acad. Sci. U.S.A. 1967, 57, 250–257. 10.1073/pnas.57.2.250. 16591461 PMC335497

[ref39] Jorgensen W. L.; Pranata J. Importance of Secondary Interactions in Triply Hydrogen Bonded Complexes: Guanine-Cytosine vs Uracil-2,6-Diaminopyridine. J. Am. Chem. Soc. 1990, 112, 2008–2010. 10.1021/ja00161a061.

