# Supplementary material for: Quantitative Measurement of Cooperativity in H-Bonded Networks
Source: J Am Chem Soc. 2022 Oct 12;144(42):19499–507. doi: 10.1021/jacs.2c08120 (PMC9619404; doi:10.1021/jacs.2c08120)
Supplement: Supplementary file 1 — ja2c08120_si_001.pdf [file ja2c08120_si_001.pdf]

Supporting Information

Quantitative Measurement of Cooperativity in H-Bonded Networks.

Lucia Trevisan, Andrew D. Bond and Christopher A. Hunter*

[a] Yusuf Hamied Department of Chemistry, University of Cambridge, Lensfield Road, Cambridge, CB2 1EW, UK.

* herchelsmith.orgchem@ch.cam.ac.uk

Table of contents

Table of contents	Page
**Materials and Methods**	S2
**Synthesis of Compounds**	S4
**NMR Characterisation of 2, 3, 4, 10, 11, 12, 13**	S13
**X-ray Crystallography**	S34
**NMR Experiments**	S38
**^{1}H-NMR Titration and Dilution**	S38
**NOESY Experiments**	S42
**UV-Vis experiments**	S46
**Molecule 1**	S48
**Molecule 2**	S52
**Molecule 3**	S56
**Molecule 4**	S60
**Molecule 5**	S64
**Molecule 6**	S67
**Details of computational study**	S71
**References**	S73

Materials and Methods

All reagents were purchased from commercial sources (Sigma Aldrich UK, Acros, Fluorochem) and were used as received without any further purification. Dry solvents were obtained by means of a Grubbs solvent system.

Flash chromatography was done with an automated system (Combiflash Companion) using pre-packed cartridges of silica (50 µm PuriFlash® column) or reverse phase C18HP (15 µm, PuriFlash® column).

The LC-MS analysis of samples was performed using Waters Acquity H-class UPLC coupled with a single quadrupole Waters SQD2. ACQUITY UPLC CSH C18 Column, 130 Å, 1.7 µm, 2.1 mm X 50 mm was used as the UPLC column for all samples. The conditions of the UPLC method are as follows: Solvent A: Water +0.1% Formic acid; Solvent B: Acetonitrile +0.1% Formic acid; Gradient of 0-2 minutes 5% -100%B + 1 minute 100% B with re-equilibration time of 2 minutes. Flow rate: 0.6 ml/min; column temperature of 40°C; injection volume of 2 µL. The signal was monitored with MS-ES+, at 254nm or at 290 nm.

^{1}H-NMR and ^{13}C-NMR were recorded on a 400 MHz or 500 MHz Bruker spectrophotometer as indicated. The reference values used for the chemical shifts of the various spectra are reported in the literature.[1] The splitting pattern is indicated with the following abbreviations: s for singlet, d for doublet, t for triplet, q for quartet, quint for quintet, m for multiplet, dd for doublet of doublets and dt for doublet of triplets.

FT-IR spectra were collected with an ALPHA FT-IR Spectrometer from Bruker.

UV-Vis spectra were recorded with a UV-Vis Cary 60 spectrophotometer (Agilent).

Melting points were recorded with a Mettler Toledo MP90 melting point apparatus.

Synthesis of Compounds

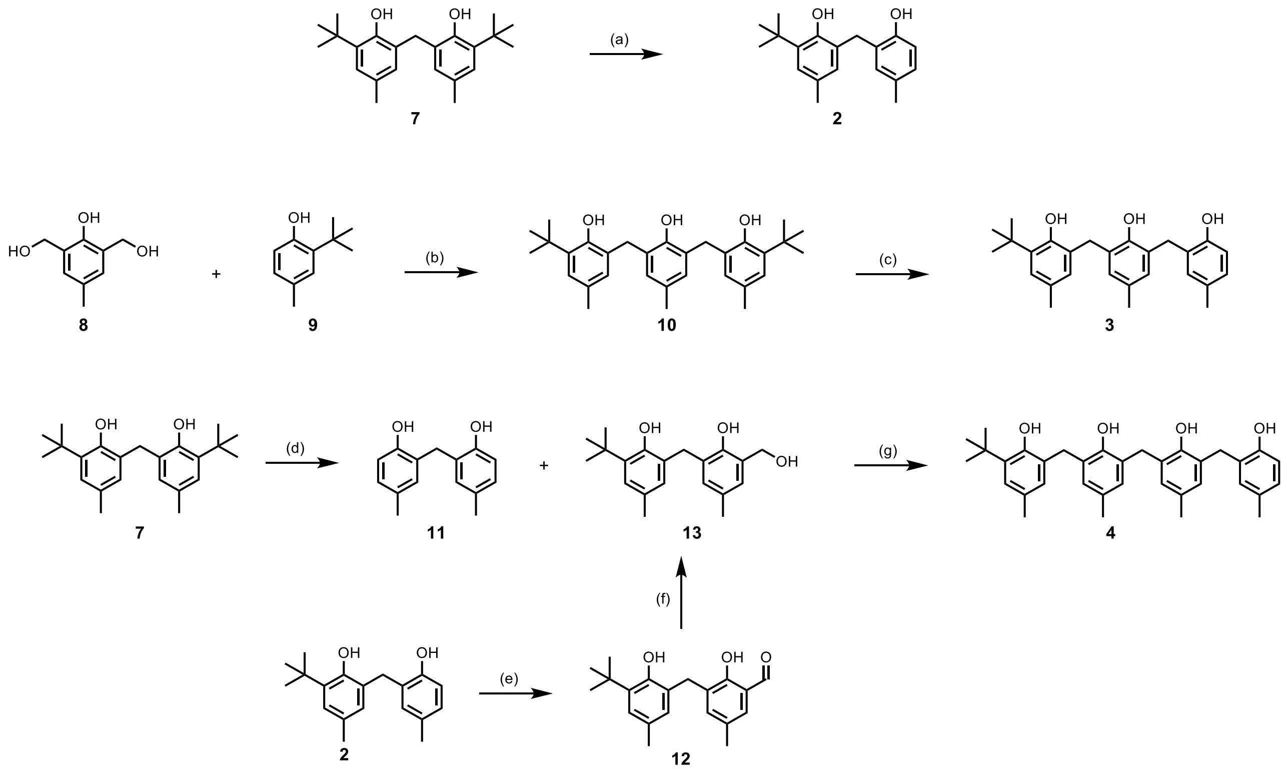

Figure S1. Synthesis of **2**, **3** and **4**. Reactions conditions: (a) $AlCl_3$, toluene, r.t., 2 h; (b) *p*-TsOH, toluene, 120°C, 96 h; (c) $AlCl_3$, toluene, r.t., 2 h; (d) $AlCl_3$, toluene, r.t., 0.5 h; (e) $(CH_2O)_n$, $MgCl_2$, Et_3N, dry MeCN, 82°C, 22 h; (f) $NaBH_4$, MeOH, r.t., 1.75 h; (g) *p*-TsOH, toluene, 120°C, 19 h.

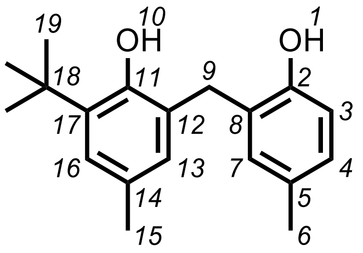

2

Modified from a previously reported procedure.[2]

Solid aluminium chloride (600.4 mg, 4.5 mmol) was added to a solution of 2,2'-methylenebis(6-*tert*-butyl-4-methylphenol) **7** (1.5434 g, 4.5 mmol) in dry toluene (30 mL). The reaction mixture was stirred for 2 hours at room temperature under an inert atmosphere. An aqueous solution of hydrochloric acid (1 M, 30 mL) was added. The mixture was extracted with diethyl ether (2x100 mL). The combined organic layers were dried over magnesium sulphate, filtered and the solvent was removed under reduced pressure. The residue was purified by flash column chromatography (SiO_2, 0-100% gradient of ethyl acetate in petroleum ether). The desired product is a white solid (417.6 mg, 1.5 mmol, 32%).

^{1}H-NMR (400 MHz, $CDCl_3$) δ_H (ppm): 7.09 (s, 1H, H(7)), 6.96 (s, 2H, H(13), H(16)), 6.89 (d, J = 8.0 Hz, 1H, H(4)), 6.66 (d, 1H, H(3)), 6.46 (s, 1H, H(10)), 5.49 (s, 1H, H(1)), 3.86 (s, 1H, H(9)), 2.26 (d, 2H, H(6), H(15)), 1.39 (s, 1H, H(19)).

^{13}C{^{1}H}-NMR (101 MHz, $CDCl_3$) δ_C (ppm): 150.2 (1C, C(11)), 150.0 (1C, C(2)), 136.7 (1C, C(17)), 131.5 (1C, C(7)), 131.2 (1C, C(5)), 129.4 (1C, C(14)), 129.0 (1C, C(16)), 128.4 (1C, C(4)), 127.3 (1C, C(12)), 126.6 (1C, C(8)), 126.4 (1C, C(13)), 115.5 (1C, C(3)), 34.7 (1C, C(18)), 31.2 (1C, C(9)), 30.1 (1C, C(19)), 21.0 (1C, C(15)), 20.7 (1C, C(6)).

HRMS: calc. for $C_{19}H_{24}O_2$ $[M]^+$ 284.1776, found 284.1764.

IR spectrum ṽ (cm^{-1}): 3563-3292 ($\nu_{O\text{-}H}$), 3002 ($\nu_{C\text{-}H\ alkene}$), 2952-2867 ($\nu_{C\text{-}H\ alkane}$), 1507 ($\nu_{C=C\ cyclic\ alkene}$), 1443 ($\delta_{C\text{-}H\ alkane,\ methyl\ group}$), 1361 ($\delta_{O\text{-}H\ phenol}$).

m.p.: 115.9-117.8°C, (lit. 116-117°C).[3]

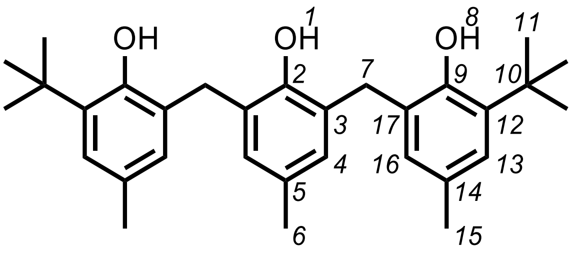

10

Modified from a previously reported procedure on a different substrate.[4]

2,6-Bis(hydroxymethyl)-*p*-cresol **8** (2.0731 g, 12 mmol), *p*-toluenesulfonic acid monohydrate (0.1942 g, 1.0 mmol) and 2-*tert*-butyl-4-methylphenol **9** (10.5042 g, 64 mmol) were dissolved in toluene (85 mL) and heated to reflux (T = 120°C) for 4 days. The solvent was dried under vacuum. The crude product was purified by flash column chromatography (SiO_2, 0-100% gradient of ethyl acetate in petroleum ether). The desired product was obtained as a white solid (0.9360 g, 2.0 mmol, 16%).

^{1}H-NMR (400 MHz, $CDCl_3$) δ_H (ppm): 7.80 (s, 1H, H(1)), 7.02-6.87 (m, 6H, H(4), H(13), H(16)), 6.49 (s, 2H, H(8)), 3.83 (s, 4H, H(7)), 2.24 (s, 9H, H(6), H(15)), 1.39 (s, 18H, H(11)).

^{13}C{^{1}H}-NMR (101 MHz, $CDCl_3$) δ_C (ppm): 149.5 (2C, C(9)), 148.0 (1C, C(2)), 136.2 (2C, C(12)), 130.9 (1C, C(5)), 129.9 (2C, C(14)), 129.8 (2C, C(4)), 129.2 (2C, C(16)), 127.7 (2C, C(17)), 127.2 (2C, C(3)), 126.2 (2C, C(13)), 34.1 (2C, C(10)), 31.3 (2C, C(7)), 30.0 (6C, C(11)), 20.7 (2C, C(15)), 20.4 (1C, C(6)).

HRMS: calc. for $C_{31}H_{41}O_3$ $[M+H]^+$ 461.3056, found 461.3034.

IR spectrum $\tilde{\nu}$ (cm^{-1}): 3334 ($\nu_{\text{O-H alcohol, intermolecular bonded}}$), 3004 ($\nu_{\text{C-H alkene}}$), 2952-2866 ($\nu_{\text{C-H alkane}}$), 1480 ($\nu_{\text{C=C cyclic alkene}}$), 1444 ($\delta_{\text{C-H alkane, methyl group}}$), 1361 ($\delta_{\text{O-H phenol}}$).

m.p.: 171.8-174.0°C (lit. 174-175°C).[5]

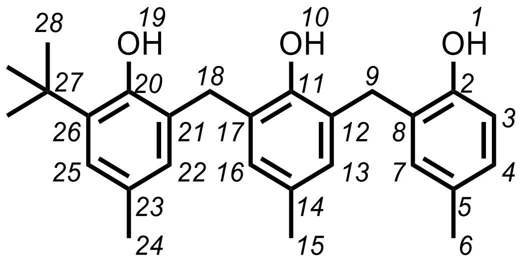

3

Modified from a previously reported procedure.[2]

Solid aluminium chloride (144.7 mg, 1.1 mmol) was added to a solution of **10** (0.5018 g, 1.1 mmol) in dry toluene (14 mL). The reaction mixture was stirred at room temperature for 2 hours under an inert atmosphere. A 10% aqueous solution of hydrochloric acid (10 mL) was added to the reaction mixture and it was extracted with diethyl ether (2x50 mL). The combined organic phases were dried over magnesium sulphate and the solvent was removed reduced pressure. The crude product was purified by flash column chromatography (SiO_2, 0-10% gradient of ethyl acetate in petroleum ether). The desired product was obtained as a white solid (121.7 mg, 0.3 mmol, 28%).

^{1}H-NMR (400 MHz, $CDCl_3$) δ_H (ppm): 8.00 (s, 1H, H(10)), 7.14 (s, 1H, H(19)), 7.05 (d, J = 1.6 Hz, 2H, H(7)), 6.94 (dt, J = 9.2 Hz, 2.6 Hz, 4H, H(13), H(16), H(22), H(25)), 6.86 (dd, J = 8.1 Hz, 2.1 Hz, 1H, H(4)), 6.62 (d, J = 8.1 Hz, 1H, H(3)), 6.24 (s, 1H, H(1)), 3.83 (s, 4H, H(9), H(18)), 2.31 – 2.16 (m, 9H, H(6), H(15), H(24)), 1.39 (s, 9H, H(28)).

^{13}C{^{1}H}-NMR (101 MHz, $CDCl_3$) δ_C (ppm): 149.9 (1C, C(20)), 149.7 (1C, C(2)), 147.7 (1C, C(11)), 136.8 (1C, C(26)), 131.5 (1C, C(7)), 131.3 (1C, C(5)), 131.0, 129.5 (1C, C(14), C(23)), 129.8, 129.6, 129.1 (1C, C(13), C(16), C(22)), 128.5 (1C, C(4)), 128.1, 127.9, 127.2 (1C, C(12), C(17), C(21)), 126.7 (1C, C(8)), 126.2 (1C, C(25)), 115.5 (1C, C(3)), 34.7 (1C, C(27)), 31.7 (1C, C(18)), 31.3 (1C, C(9)), 30.1 (3C, C(28)), 21.0 (1C, C(24)), 20.7 (1C, C(15)), 20.6 (1C, C(6)).

HRMS: calc. for $C_{27}H_{31}O_3$ $[M-H]^-$ 403.2279, found 403.2280.

IR spectrum $\tilde{\nu}$ (cm^{-1}): 3197 ($\nu_{\text{O-H alcohol, intramolecular bonded}}$), 2952 ($\nu_{\text{C-H alkene}}$), 2919-2864 ($\nu_{\text{C-H alkane}}$), 1503 ($\nu_{\text{C=C cyclic alkene}}$), 1449 ($\delta_{\text{C-H alkane, methyl group}}$), 1378 ($\delta_{\text{O-H phenol}}$).

m.p.: 179.0-180.2°C (170-172°C,[2] 178.3°C[6]).

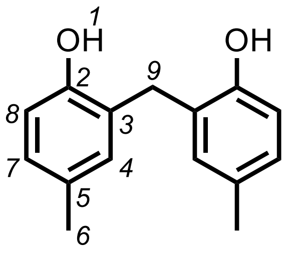

11

Modified from a previously reported procedure.[2]

Solid aluminium chloride (850.2 mg, 6.4 mmol) was added to a solution of 2,2'-methylenebis(6-*tert*-butyl-4-methylphenol) **7** (1.0746 g, 3.2 mmol) in dry toluene (32.0 mL). The reaction mixture was stirred for 30 minutes at room temperature under an inert atmosphere. An aqueous solution of hydrochloric acid (1 M, 30 mL) was added. The mixture was extracted with diethyl ether (2x100 mL). The combined organic layers were dried over magnesium sulphate, filtered and the solvent was removed under reduced pressure. The residue was purified by flash column chromatography (SiO_2, 0-100% gradient of ethyl acetate in petroleum ether). The desired product is a white solid (607.9 mg, 2.7 mmol, 84%).

^{1}H-NMR (400 MHz, $CDCl_3$) δ_H (ppm): 7.08 (d, J = 2.1 Hz, 2H, H(4)), 6.89 (dd, J = 8.2 Hz, 2.1 Hz, 2H, H(7)), 6.78-6.66 (m, 4H, H(1), H(8)), 3.85 (s, 2H, H(9)), 2.26 (s, 6H, H(6)).

^{13}C{^{1}H}-NMR (101 MHz, $CDCl_3$) δ_C (ppm): 150.4 (2C, C(2)), 131.4 (2C, C(4)), 130.8 (2C, C(5)), 128.6 (2C, C(7)), 126.7 (2C, C(3)), 115.9 (2C, C(8)), 31.0 (1C, C(9)), 20.7 (2C, C(6)).

HRMS: calc. for $C_{15}H_{16}O_2$ $[M]^+$ 228.1150, found 228.1157.

IR spectrum $\tilde{\nu}$ (cm^{-1}): 3255-3239 ($\nu_{O\text{-}H}$, intramolecular bonded), 3055-3015 ($\nu_{C\text{-}H\ alkene}$), 2921-2862 ($\nu_{C\text{-}H\ alkane}$), 1613-1594 ($\nu_{C=C\ cyclic\ alkene}$), 1456 ($\delta_{C\text{-}H\ alkane,\ methyl\ group}$), 1380-1358 ($\delta_{O\text{-}H\ phenol}$).

m.p.: 124.9-126.3°C (lit. 125-126°C).[2]

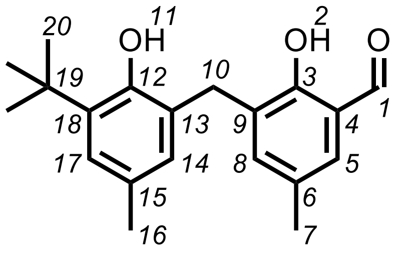

12

Modified from a previously reported procedure on a different substrate.[7]

To a solution of **2** (539.7 mg, 1.9 mmol) in dry acetonitrile (5.0 mL), magnesium chloride (287.0 g, 3.0 mmol), triethylamine (1.0 mL, 7.2 mmol) and paraformaldehyde (437.8 mg, 15 mmol) were added. The reaction mixture was stirred at reflux (T=82°C) under an inert atmosphere for 22 hours. The reaction was cooled to room temperature and then quenched with an aqueous solution of hydrochloric acid (1 M, 8.0 mL). The aqueous layer was extracted with ethyl acetate (3x50 mL). The combined organic phases were washed with brine (1x150 mL) and then they were dried over magnesium sulphate, filtered and concentrated under reduced pressure. The crude product was purified by flash column chromatography (SiO_2, 0-10% gradient of ethyl acetate in petroleum ether). Long transparent needle-like crystals were obtained as the product (0.475 g, 1.5 mmol, yield 80%).

^{1}H-NMR (400 MHz, $CDCl_3$) δ_H (ppm): 11.76 (s, 1H, H(2)), 9.82 (s, 1H, H(1)), 7.39 (d, J = 1.5 Hz, 1H, H(8)), 7.23 (d, J = 1.5 Hz, 1H, H(5)), 6.97 (d, J = 1.7 Hz, 1H, H(17)), 6.93 (d, J = 1.7 Hz, 1H, H(14)), 6.89 (s, 1H, H(11)), 3.89 (s, 2H, H(10)), 2.41 – 2.18 (m, 6H, H(7), H(16)), 1.39 (s, 9H, H(20)).

^{13}C{^{1}H}-NMR (101 MHz, $CDCl_3$) δ_C (ppm): 197.0 (1C, C(1)), 155.6 (1C, C(3)), 150.7 (1C, C(12)), 139.1 (1C, C(8)), 137.7 (1C, C(18)), 132.1 (1C, C(5)), 130.4 (1C, C(6)), 129.1 (1C, C(15)), 129.0 (1C, C(9)), 128.8 (1C, C(14)), 126.7 (1C, C(13)), 126.6 (1C, C(17)), 120.3 (1C, C(4)), 35.0 (1C, C(19)), 29.9 (4C, C(10), C(20)), 21.0 (1C, C(16)), 20.5 (1C, C(7)).

HRMS: calc. for $C_{20}H_{24}O_3$ $[M]^+$ 312.1725, found 312.1721.

IR spectrum $\tilde{v}$ (cm^{-1}): 3412 ($v_{O\text{-}H\ alcohol,\ intermolecular\ bonded}$), 3001 ($v_{C\text{-}H\ alkene}$), 2946-2862 ($v_{C\text{-}H\ alkane}$), 1651 ($v_{C=O\ conjugated\ aldehyde}$), 1620 ($v_{C=C\ cyclic\ alkene}$), 1445 ($\delta_{C\text{-}H\ alkane,\ methyl\ group}$), 1389-1323 ($\delta_{O\text{-}H\ phenol}$).

m.p.: 176.5°-178.5°C.

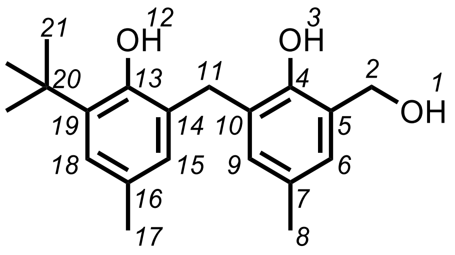

13

Modified from a previously reported procedure on a different substrate.[8]

To a vigorously stirred solution of **12** (403.9 mg, 1.3 mmol) in methanol (5.0 mL) cooled to 0°C, sodium borohydride (204.2 g, 5.4 mmol) was added. The reaction mixture was then warmed to room temperature and stirred for 1.75 hours at room temperature. The solvent was removed under reduced pressure. The residue was dissolve in ethyl acetate (100 mL) and the organic phase was washed with an aquoues solution of hydrochloric acid 3 M (3x50 mL) and with brine (1x50 mL). The organic phase was dried over magnesium sulphate, filtered and concentrated under reduces pressure. The crude product was purified by flash column chromatography (SiO_2, 0-100% gradient of ethyl acetate in petroleum ether). A white solid was obtained as the product (0.3465 g, 1.1 mmol, yield 85%).

^{1}H-NMR (400 MHz, $CDCl_3$) δ_H (ppm): 8.36 (s, 1H, H(3)), 7.31 (s, 1H, H(12)), 7.07 (d, 1H, H(9)), 6.99-6.91 (m, 2H, H(15), H(18)), 6.66 (d, 1H, H(6)), 4.84 (d, 2H, H(2)), 3.85 (s, 2H, H(11)), 2.24 (d, 7H, H(1), H(8), H(17)), 1.40 (s, 9H, H(21)).

^{13}C{^{1}H}-NMR (101 MHz, $CDCl_3$) δ_C (ppm): 150.7 (1C, C(13)), 150.2 (1C, C(4)), 137.3 (1C, C(19)), 131.1 (1C, C(9)), 130.2 (1C, C(7)), 128.9 (1C, C(16)), 128.8 (1C, C(15)), 128.2 (1C, C(10)), 127.7 (1C, C(14)), 126.4 (1C, C(6)), 126.2 (1C, C(18)), 123.7 (1C, C(5)), 65.4 (1C, C(2)), 34.9 (1C, C(20)), 30.8 (1C, C(11)), 29.9 (1C, C(21)), 21.0 (1C, C(17)), 20.6 (1C, C(8)).

HRMS: calc. for $C_{20}H_{25}O_3$ $[M\text{-}H]^-$ 313.1804, found 313.1820.

IR spectrum $\tilde{\nu}$ (cm^{-1}): 3352 ($\nu_{O\text{-}H\ alcohol,\ intermolecular\ bonded}$), 3001 ($\nu_{C\text{-}H\ alkene}$), 2951-2989 ($\nu_{C\text{-}H\ alkane}$), 1443 ($\delta_{C\text{-}H\ alkane,\ methyl\ group}$), 1390-1360 ($\delta_{O\text{-}H\ phenol}$).

m.p.: 119.7°-121.0°C.

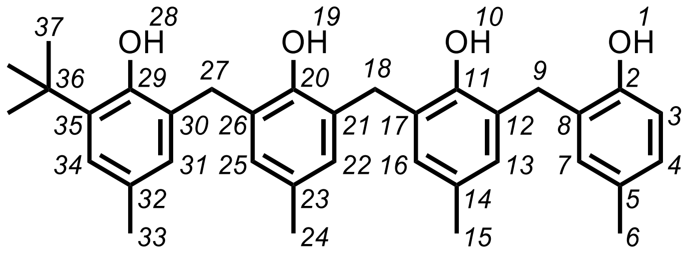

4

13 (272.5 mg, 0.9 mmol), **11** (1.0956 g, 4.8 mmol) and *p*-toluensulfonic acid monohydrate (50.9 mg, 0.3 mmol) were dissolved in dry toluene (35.0 mL) and heated to reflux (T=120°C) under an inert atmosphere for 19 hours. The solvent was removed under reduced pressure and the crude product was purified by flash column chromatography twice (SiO_2, 0-100% gradient of ethyl acetate in petroleum ether; C18, 5-100% gradient of acetonitrile in water). A white solid was obtained as the product (36.10 mg, 0.1 mmol, yield 8%).

^{1}H-NMR (400 MHz, $CDCl_3$) δ_H (ppm): 8.65 (s, 1H, H(19)), 8.35 (s, 1H, H(10)), 7.38 (s, 1H, H(28)), 7.06 (d, J = 2.1Hz, 1H, H(7)), 6.98-6.84 (m, 7H, H(4), H(13), H(16), H(22), H(25), H(34), H(31)), 6.60 (d, J = 8.1 Hz, 1H, H(3)), 5.91 (s, 1H, H(1)), 3.86-3.72 (m, 6H, H(9), H(18), H(27)), 3.86-3.72 (m, 12H, H(6), H(15), H(24), H(33)), 1.43 (s, 9H, H(37)).

^{13}C{^{1}H}-NMR (101 MHz, $CDCl_3$) δ_C (ppm): 150.1 (1C, C(29)), 149.6 (1C, C(2)), 147.7 (1C, C(20)), 147.3 (1C, C(11)), 137.0 (1C, C(35)), 131.5 (1C, C(7)), 131.3, 131.2, 130.9, 129.4 (4C, C(5), C(14), C(23), C(32)), 129.8, 129.7, 129.6 (4C, C(13), C(16), C(22), C(25)), 129.3 (1C, C(31)), 128.6, 128.3, 128.2, 127.5, 127.3 (4C, C(12), C(17), C(21), C(26), C(30)), 128.5 (1C, C(4)), 126.7 (1C, C(8)), 125.9 (1C, C(34)), 115.4 (1C, C(3)), 34.8 (1C, C(36)), 31.8 (2C, C(18), C(27)), 31.3 (1C, C(9)), 30.1 (1C, C(37)), 21.0, 20.7, 20.7, 20.6 (1C, C(6), C(15), C(24), C(33)).

HRMS: calc. for $C_{35}H_{40}O_4$ $[M+H]^+$ 525.3005, found 525.2985.

IR spectrum $\tilde{\nu}$ (cm^{-1}): 3152 ($\nu_{\text{O-H alcohol, intramolecular bonded}}$), 3062-3015 ($\nu_{\text{C-H alkene}}$), 2951-2989 ($\nu_{\text{C-H alkane}}$), 1438 ($\delta_{\text{C-H alkane, methyl group}}$), 1391-1360 ($\delta_{\text{O-H phenol}}$).

m.p.: 178.8°-180.0°C.

NMR Characterisation of 2, 3, 4, 10, 11, 12, 13

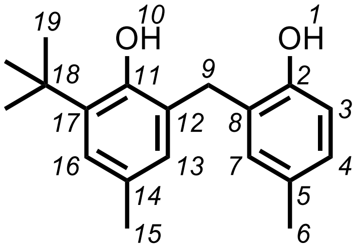

2

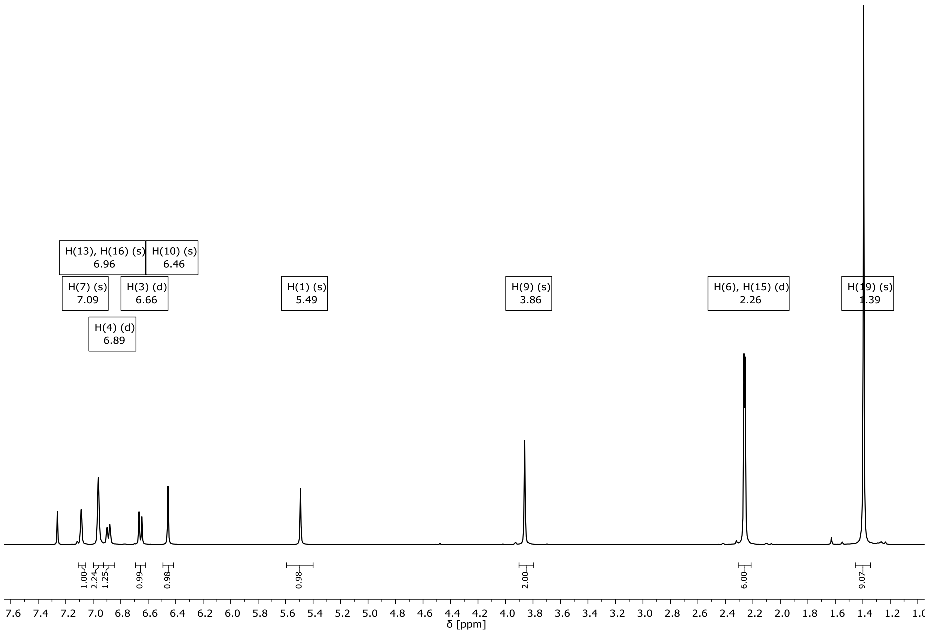

Figure S2. 400 MHz ^{1}H-NMR of **2** in $CDCl_3$.

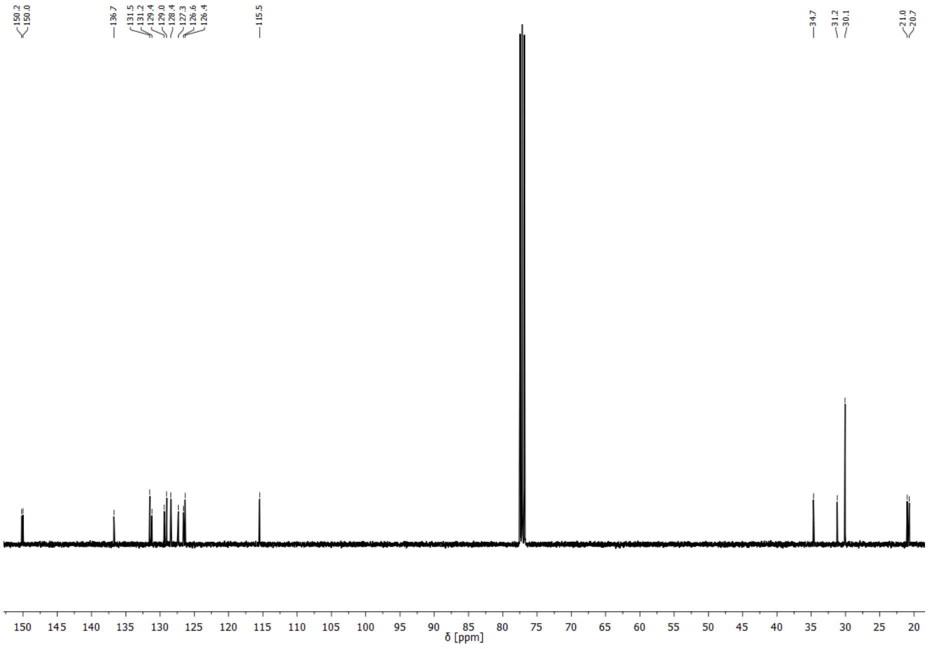

Figure S3. 400 MHz ^{13}C-NMR of **2** in $CDCl_3$.

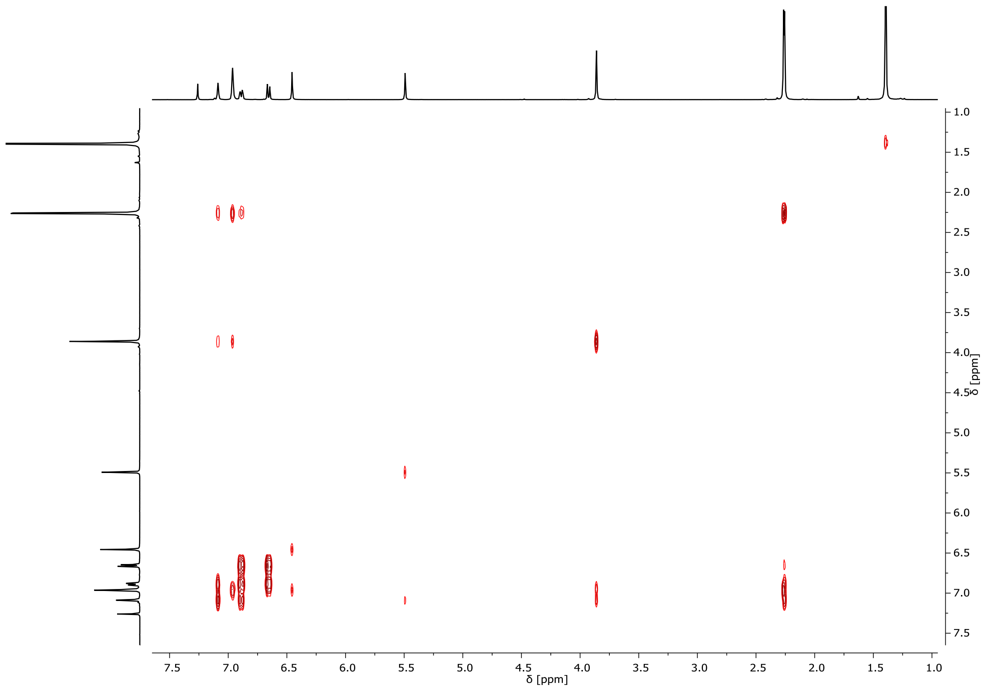

Figure S4. 400 MHz ^{1}H-^{1}H COSY spectrum of **2** in $CDCl_3$.

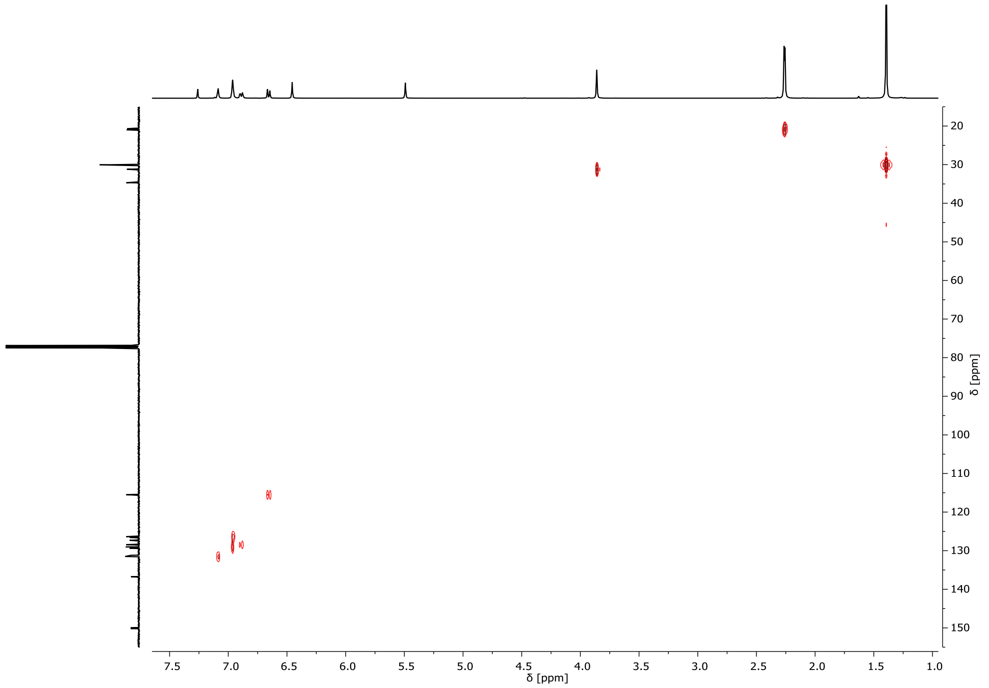

Figure S5. 400 MHz ^{1}H-^{13}C Heteronuclear Single Quantum Coherence (HSQC) spectrum of **2** in $CDCl_3$.

(a)

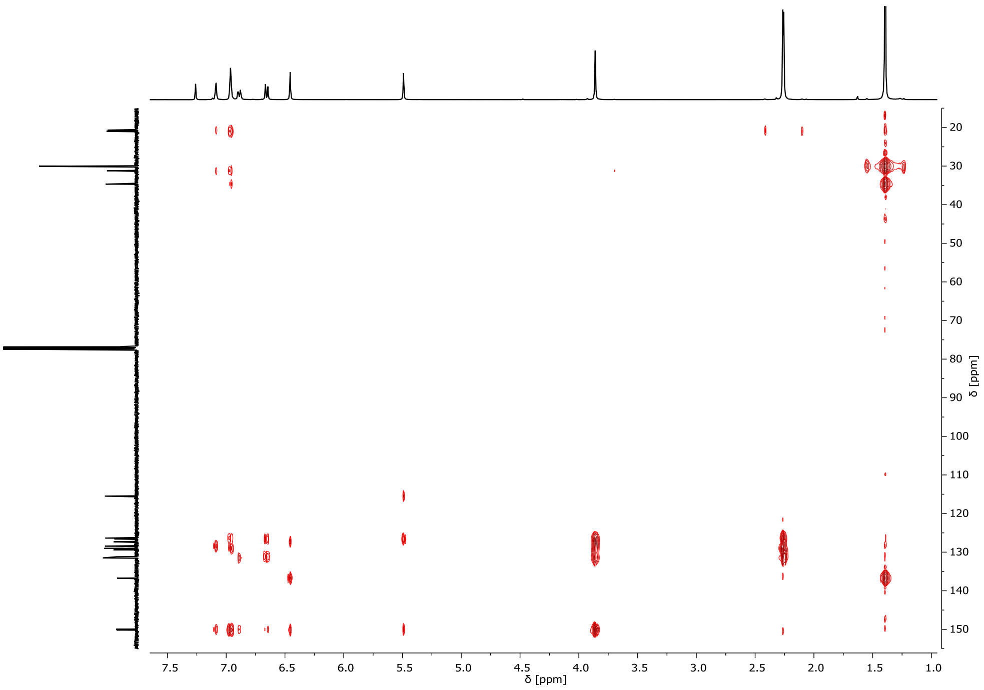

(b)

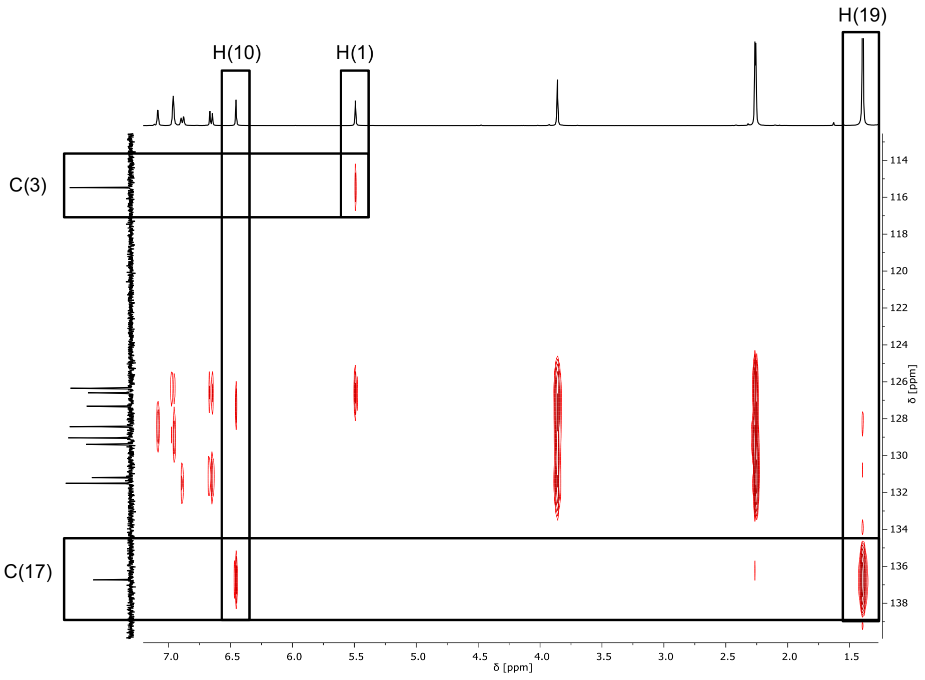

Figure S6. (a) 400 MHz ^{1}H-^{13}C Heteronuclear Multiple Bond Correlation (HMBC) spectrum of **2** in $CDCl_3$, (b) inset of HMBC spectrum of **2** in $CDCl_3$.

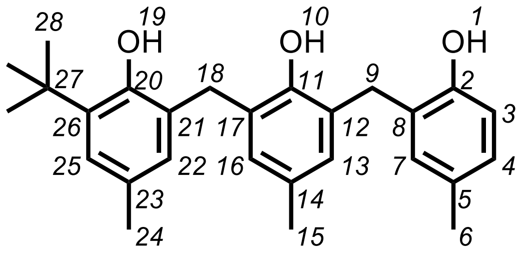

3

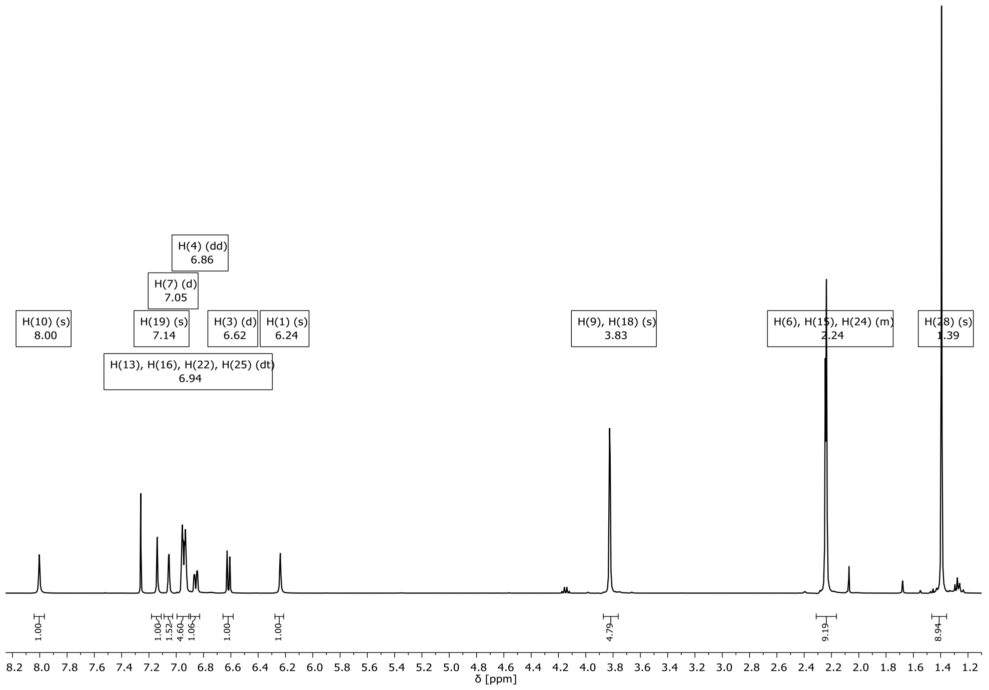

Figure S7. 400 MHz ^{1}H-NMR spectrum of **3** in $CDCl_3$.

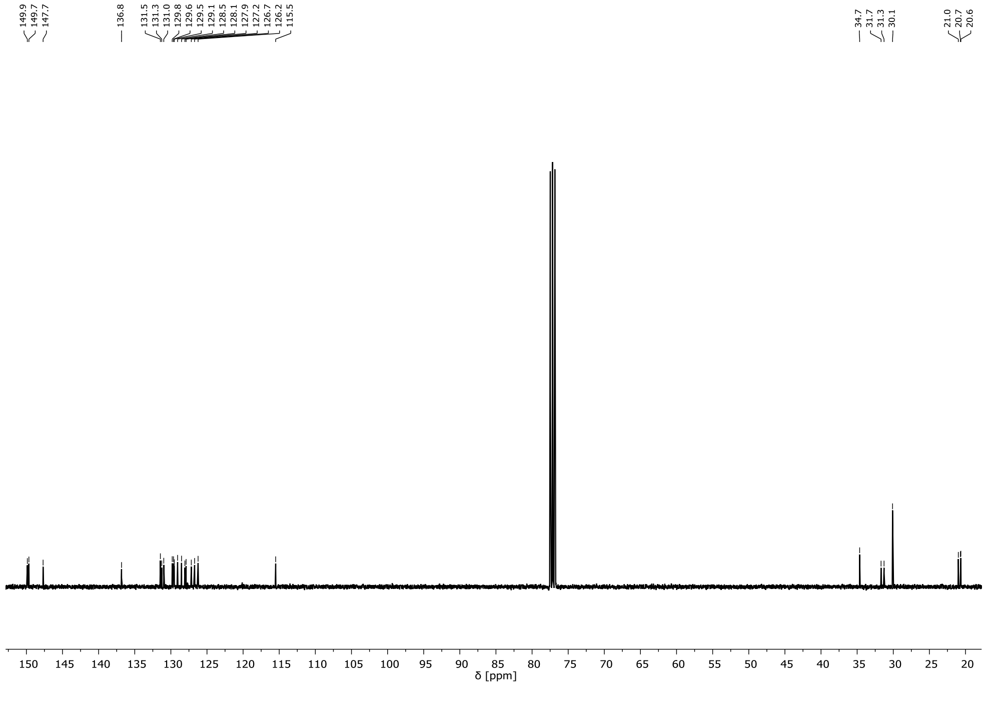

Figure S8. 400 MHz ^{13}C-NMR of **3** in $CDCl_3$.

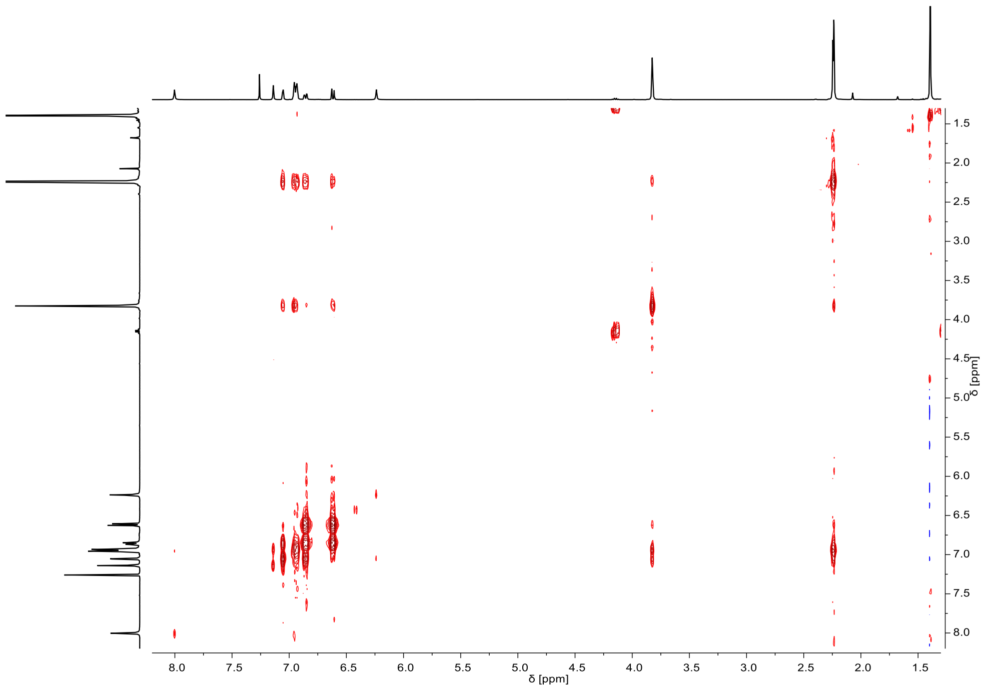

Figure S9. 400 MHz ^{1}H-^{1}H COSY spectrum of **3** in $CDCl_3$.

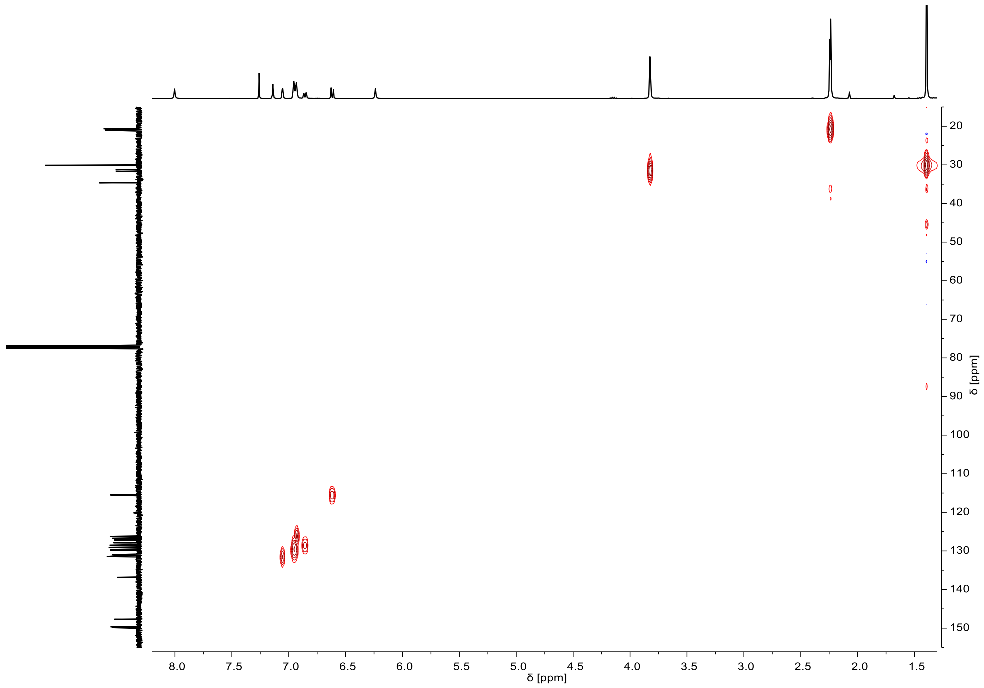

Figure S10. 400 MHz ^{1}H-^{13}C Heteronuclear Single Quantum Coherence (HSQC) spectrum of **3** in $CDCl_3$.

(a)

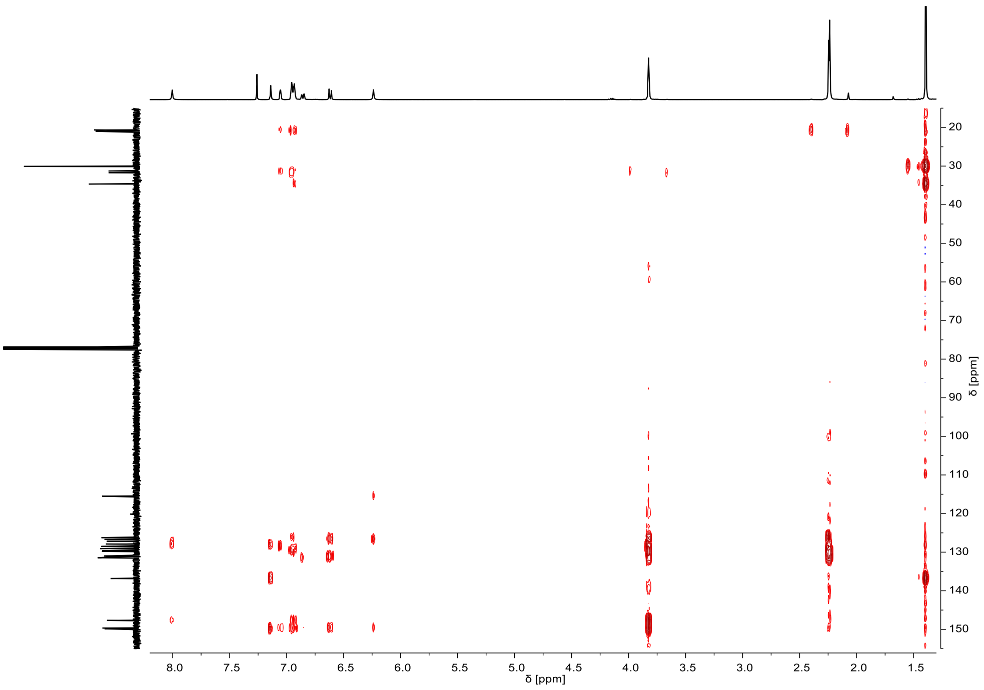

(b)

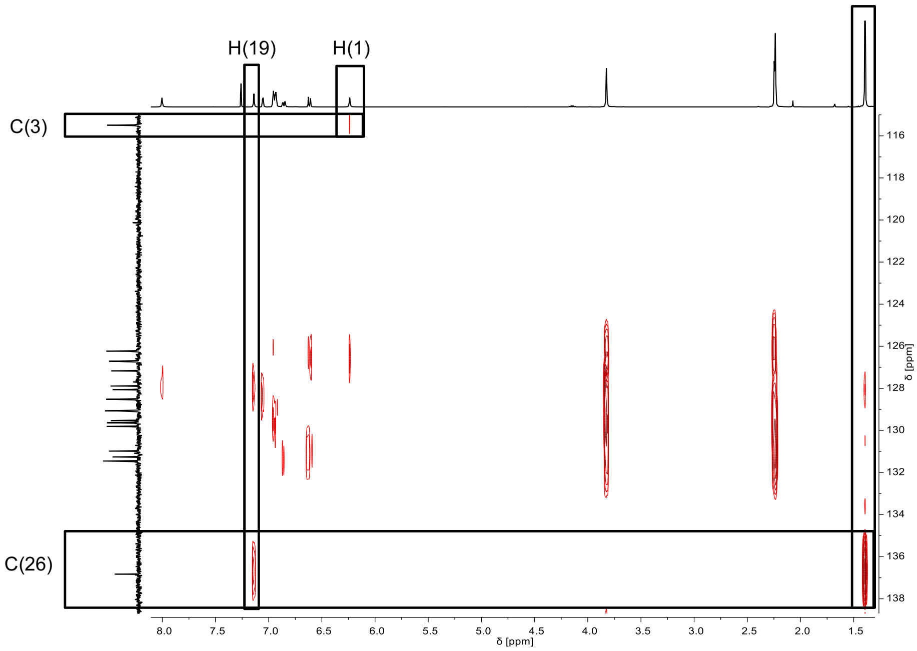

Figure S11. (a) 400 MHz ^{1}H-^{13}C Heteronuclear Multiple Bond Correlation (HMBC) spectrum of **3** in $CDCl_3$, (b) inset of HMBC spectrum of **3** in $CDCl_3$.

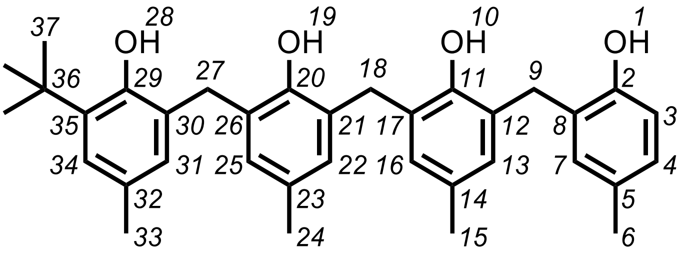

4

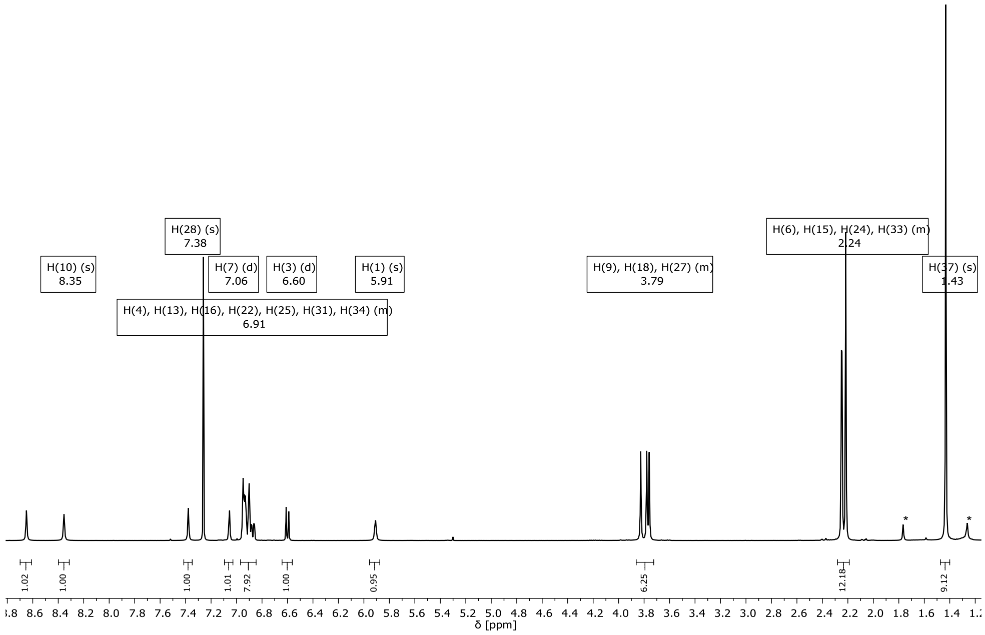

Figure S12. 400 MHz ^{1}H-NMR spectrum of **4** in $CDCl_3$.

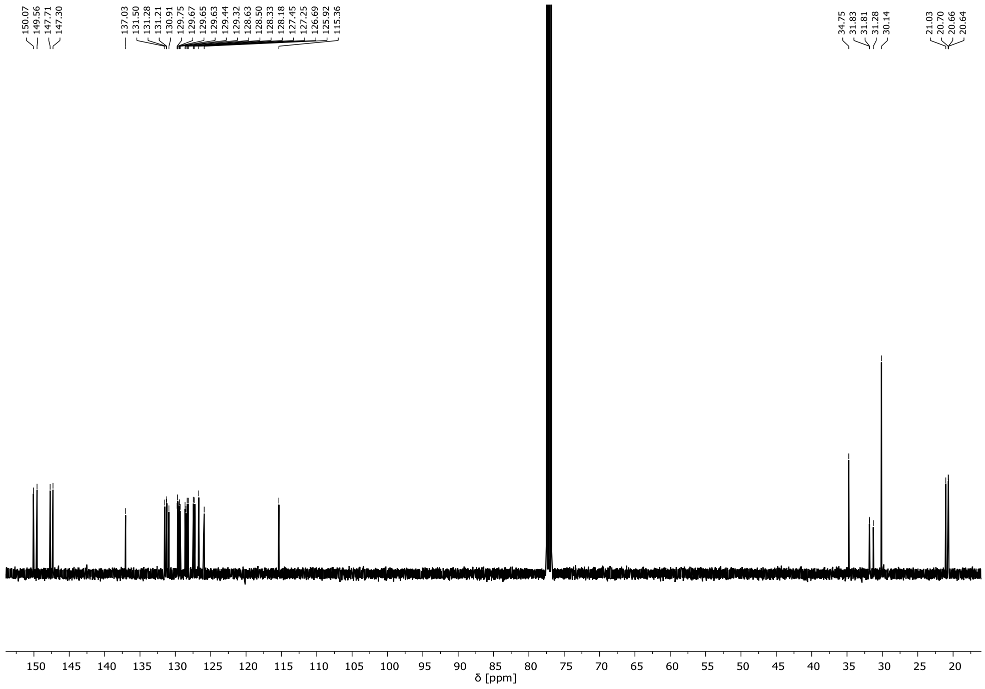

Figure S13. 400 MHz ^{13}C-NMR of **4** in $CDCl_3$.

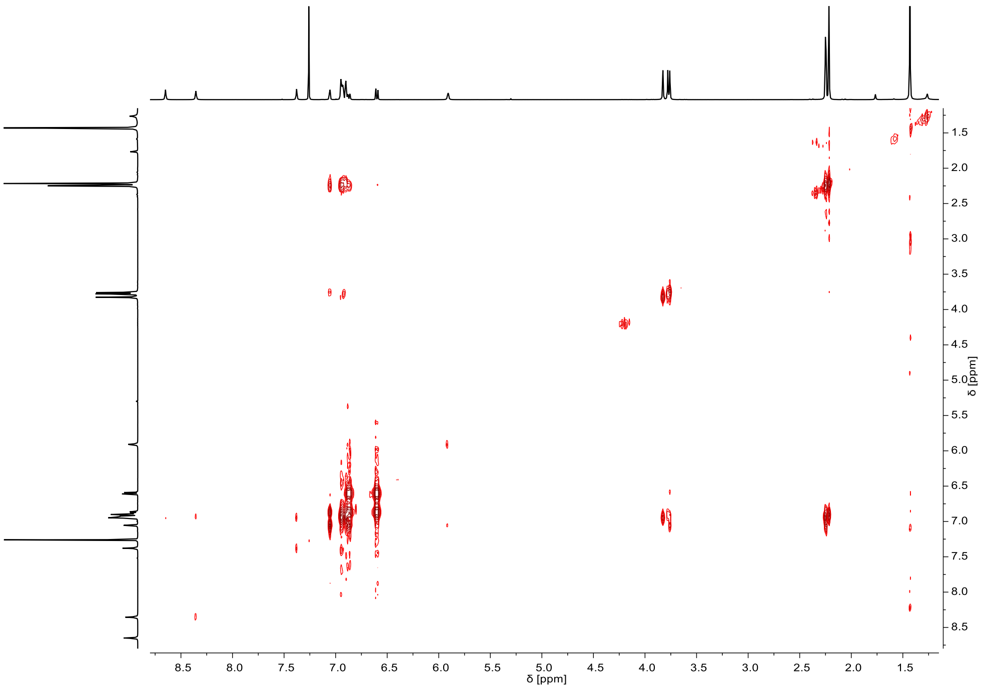

Figure S14. 400 MHz ^{1}H-^{1}H COSY spectrum of **4** in $CDCl_3$.

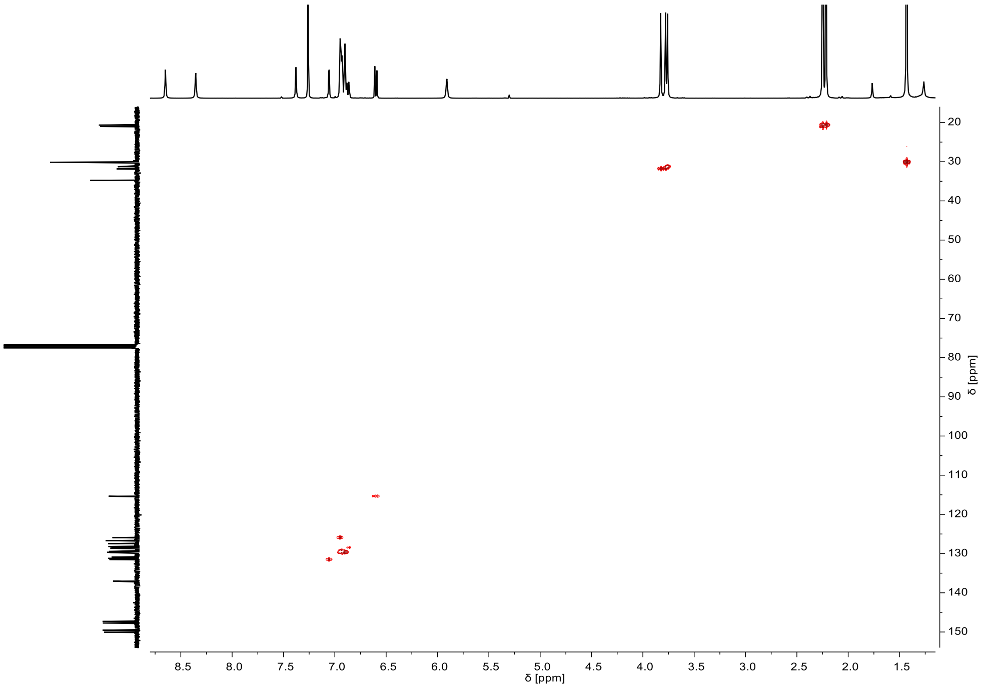

Figure S15. 400 MHz ^{1}H-^{13}C Heteronuclear Single Quantum Coherence (HSQC) spectrum of **4** in $CDCl_3$.

(a)

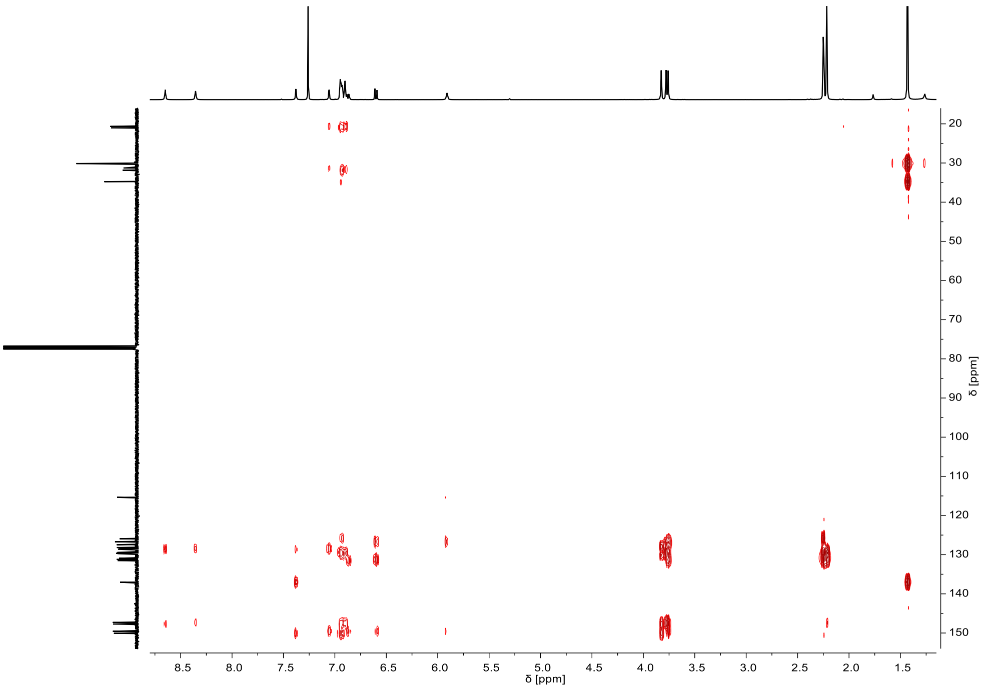

(b)

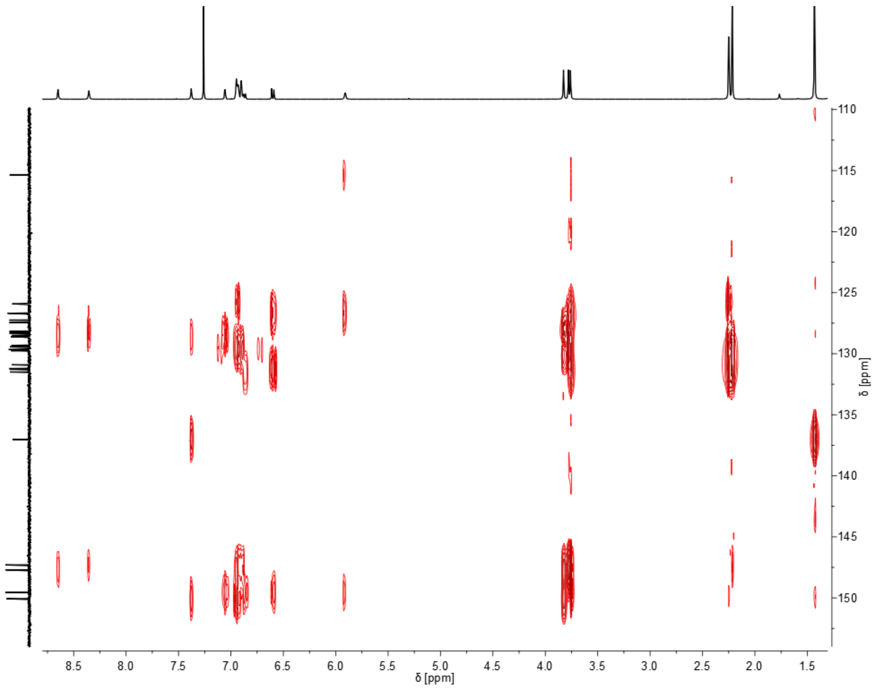

Figure S16. (a) 400 MHz ^{1}H-^{13}C Heteronuclear Multiple Bond Correlation (HMBC) spectrum of **4** in $CDCl_3$, (b) inset of HMBC spectrum of **4** in $CDCl_3$.

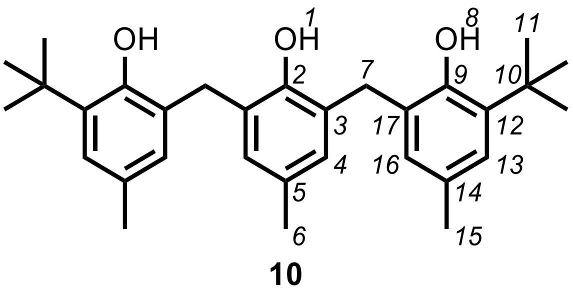

10

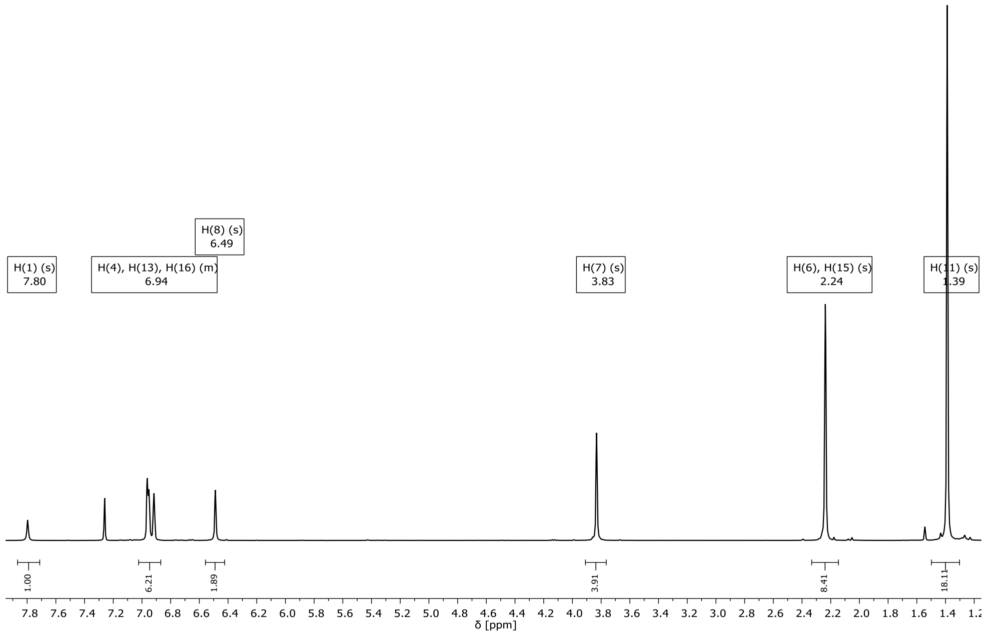

Figure S17. 400 MHz ^{1}H-NMR spectrum of **10** in $CDCl_3$.

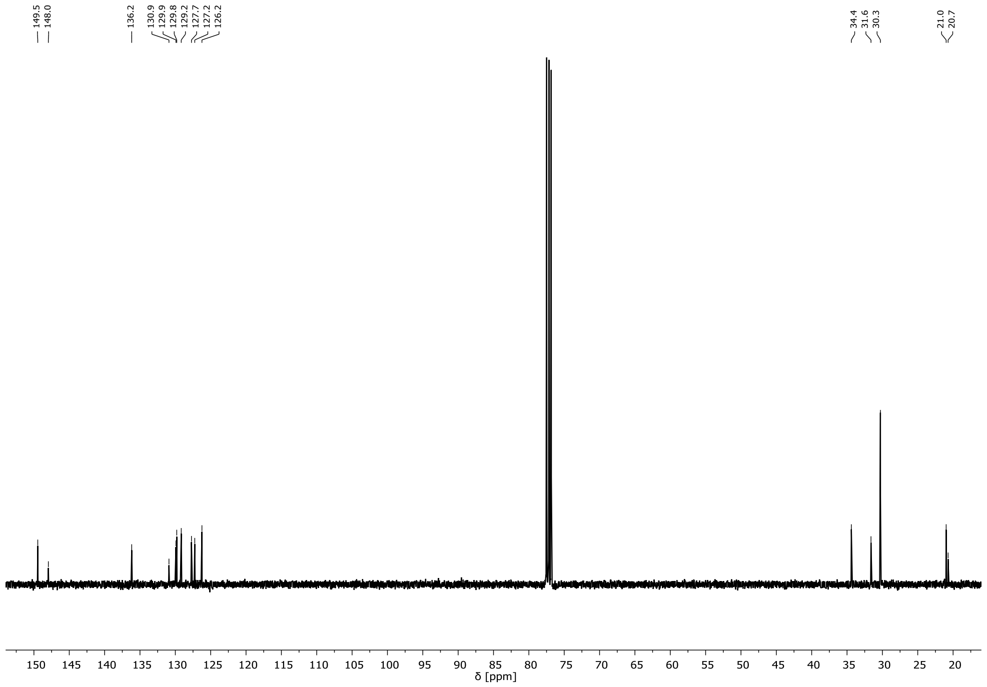

Figure S18. 400 MHz ^{13}C-NMR of **10** in $CDCl_3$.

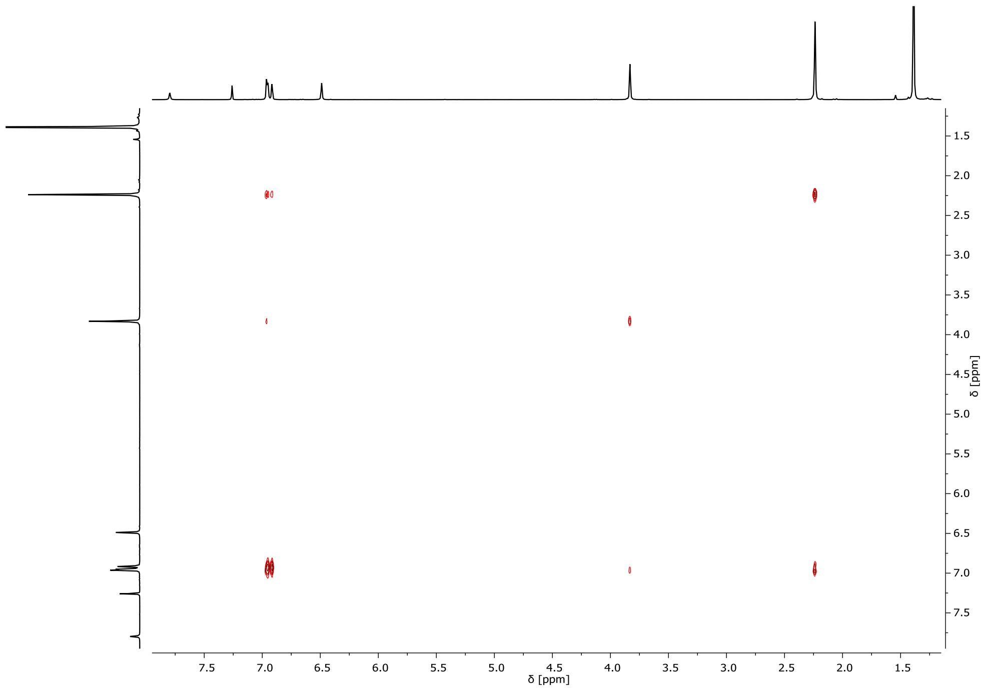

Figure S19. 400 MHz ^{1}H-^{1}H COSY spectrum of **10** in $CDCl_3$.

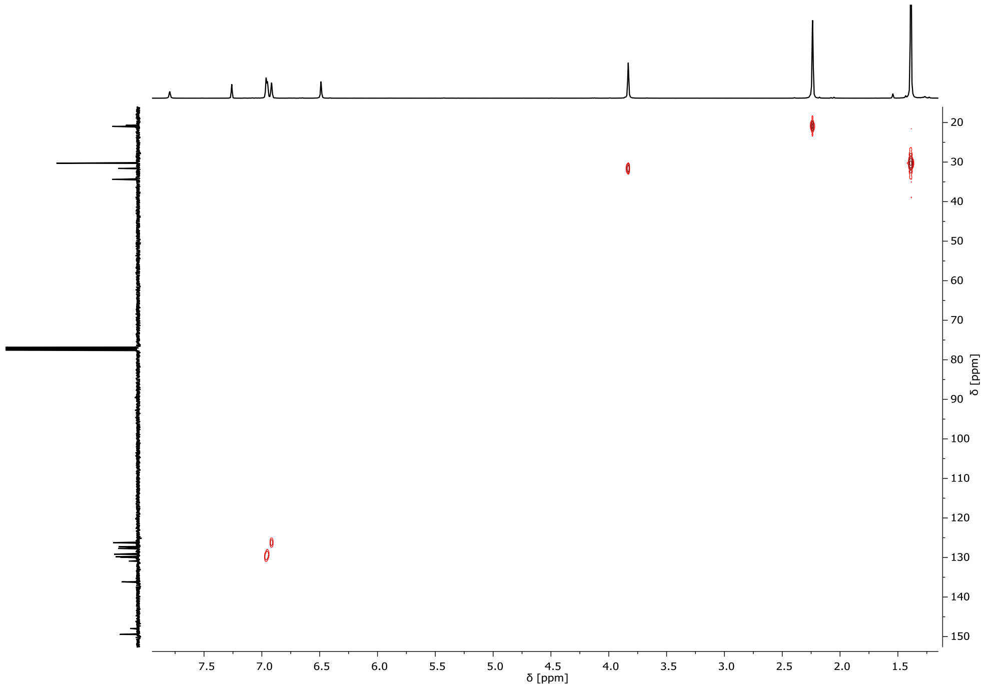

Figure S20. 400 MHz ^{1}H-^{13}C Heteronuclear Single Quantum Coherence (HSQC) spectrum of **10** in $CDCl_3$.

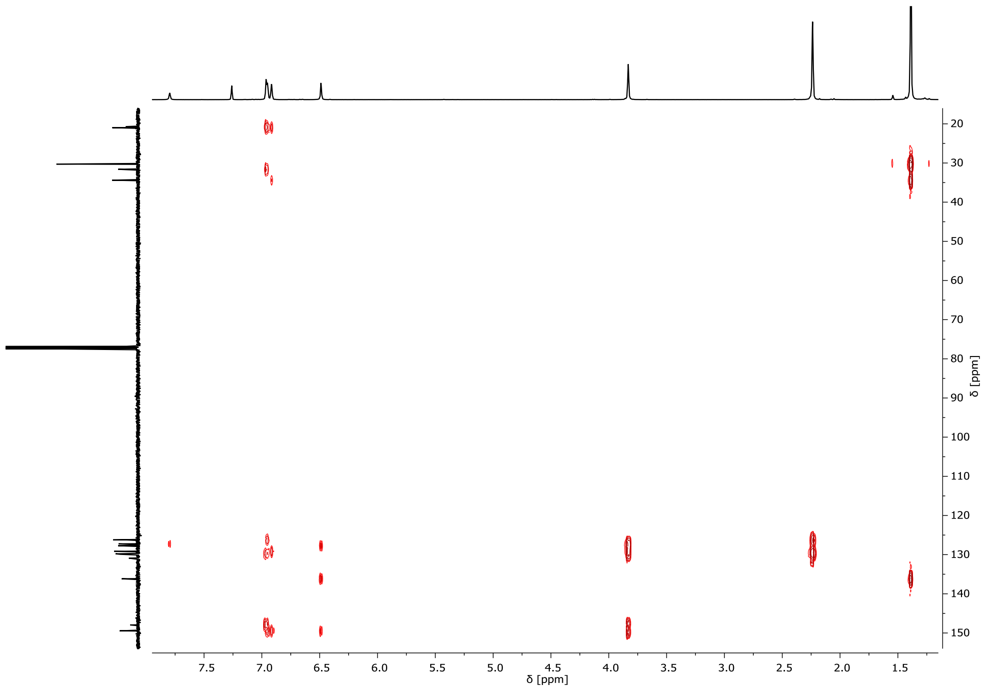

Figure S21. 400 MHz ^{1}H-^{13}C Heteronuclear Multiple Bond Correlation (HMBC) spectrum of **10** in $CDCl_3$.

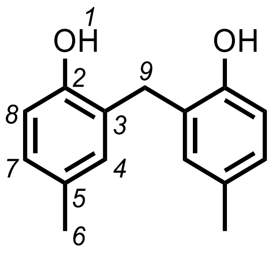

11

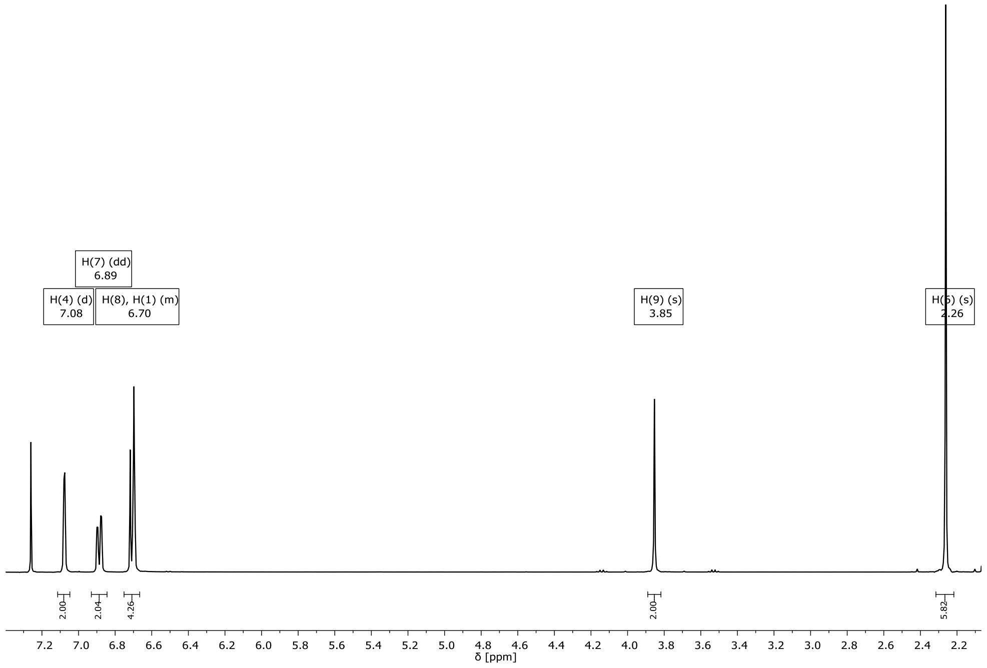

Figure S22. 400 MHz ^{1}H-NMR spectrum of **11** in $CDCl_3$.

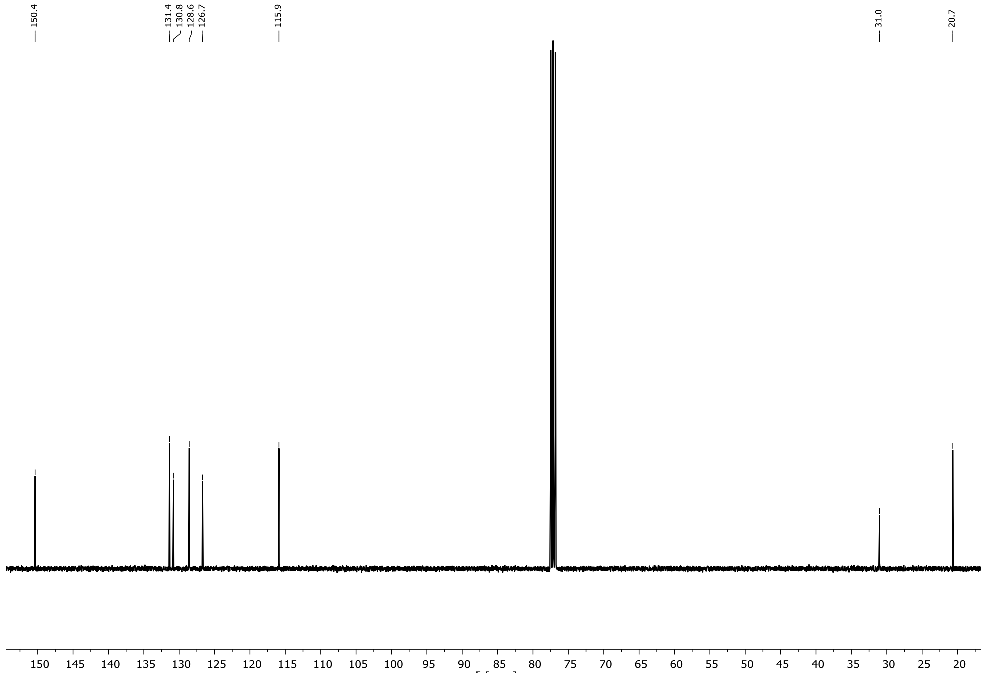

Figure S23. 400 MHz ^{13}C-NMR of **11** in $CDCl_3$.

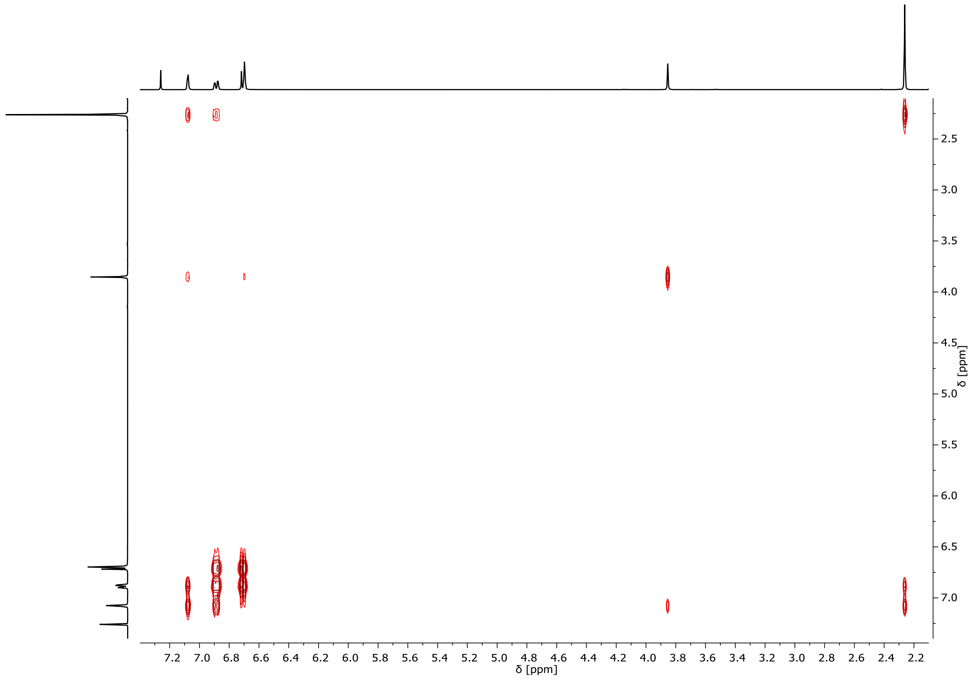

Figure S24. 400 MHz ^{1}H-^{1}H COSY spectrum of **11** in $CDCl_3$.

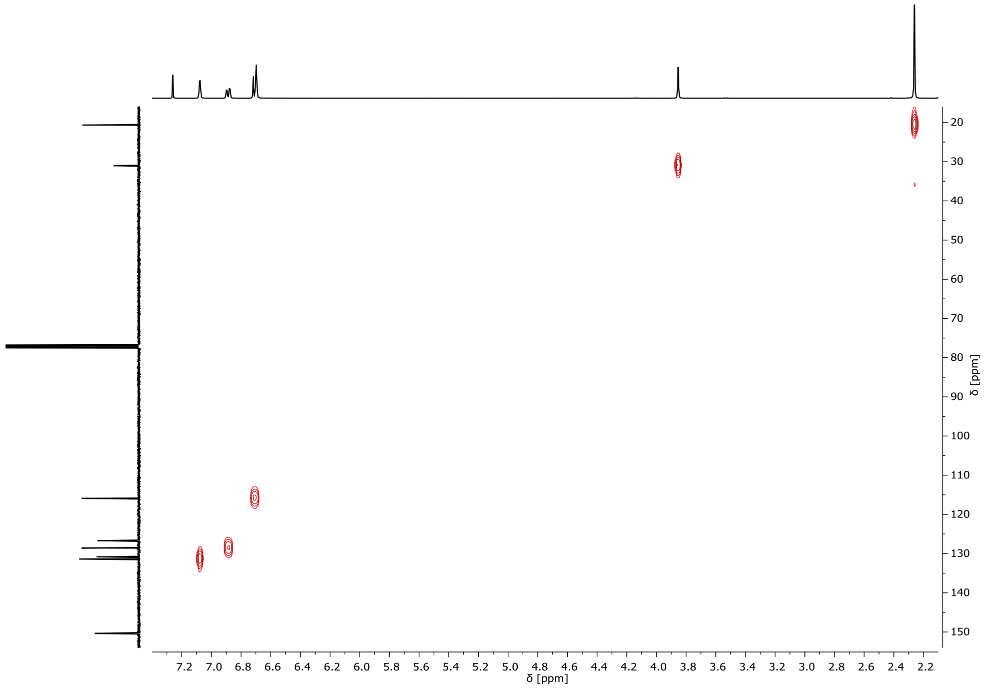

Figure S25. 400 MHz ^{1}H-^{13}C Heteronuclear Single Quantum Coherence (HSQC) spectrum of **11** in $CDCl_3$.

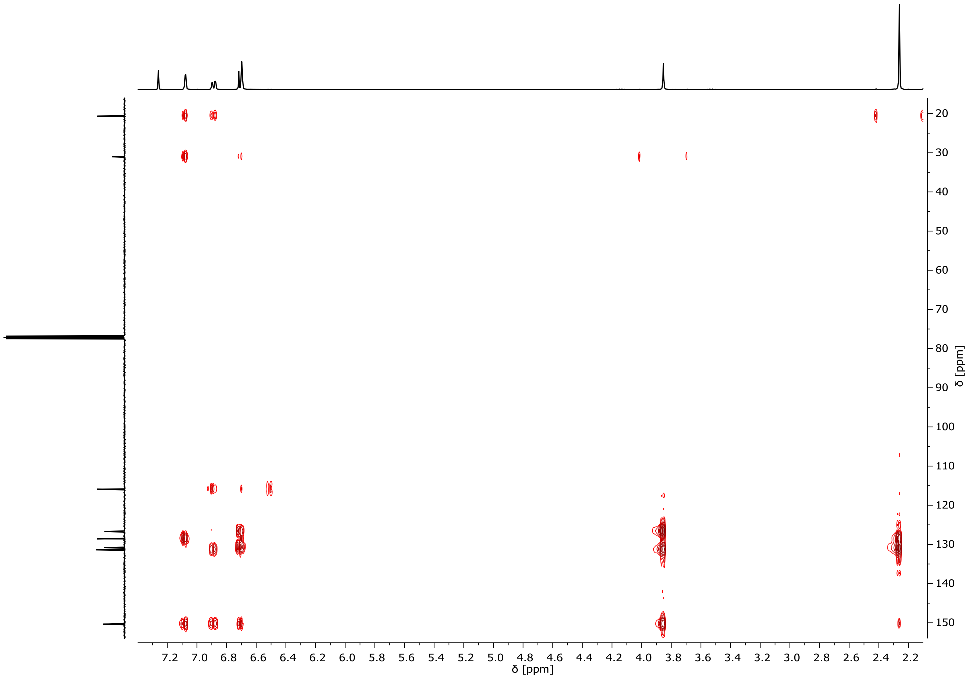

Figure S26. 400 MHz ^{1}H-^{13}C Heteronuclear Multiple Bond Correlation (HMBC) spectrum of **11** in $CDCl_3$.

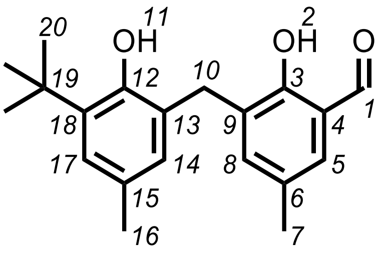

12

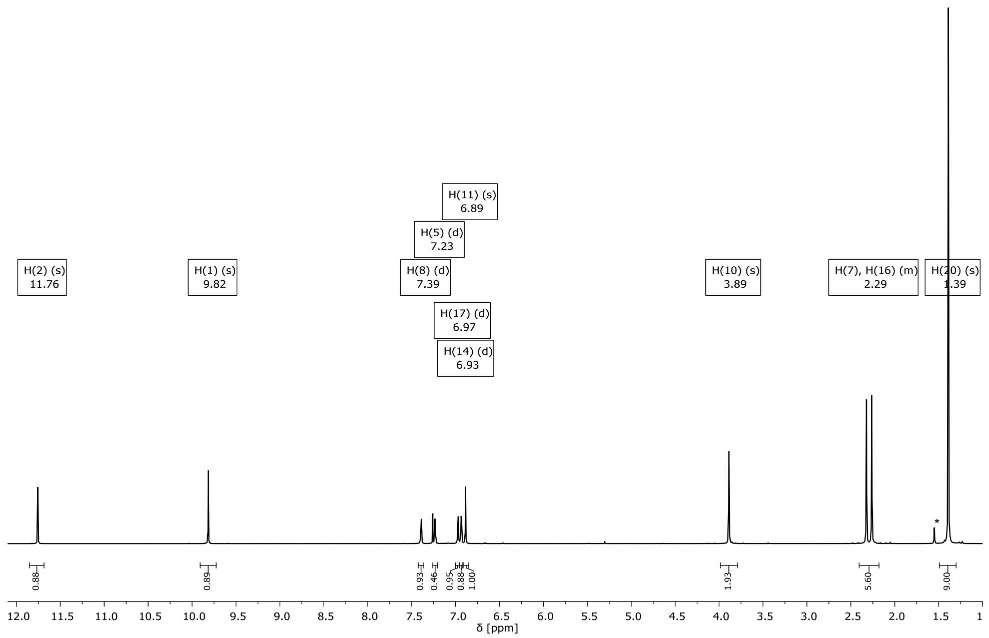

Figure S27. 400 MHz ^{1}H-NMR spectrum of **12** in $CDCl_3$.

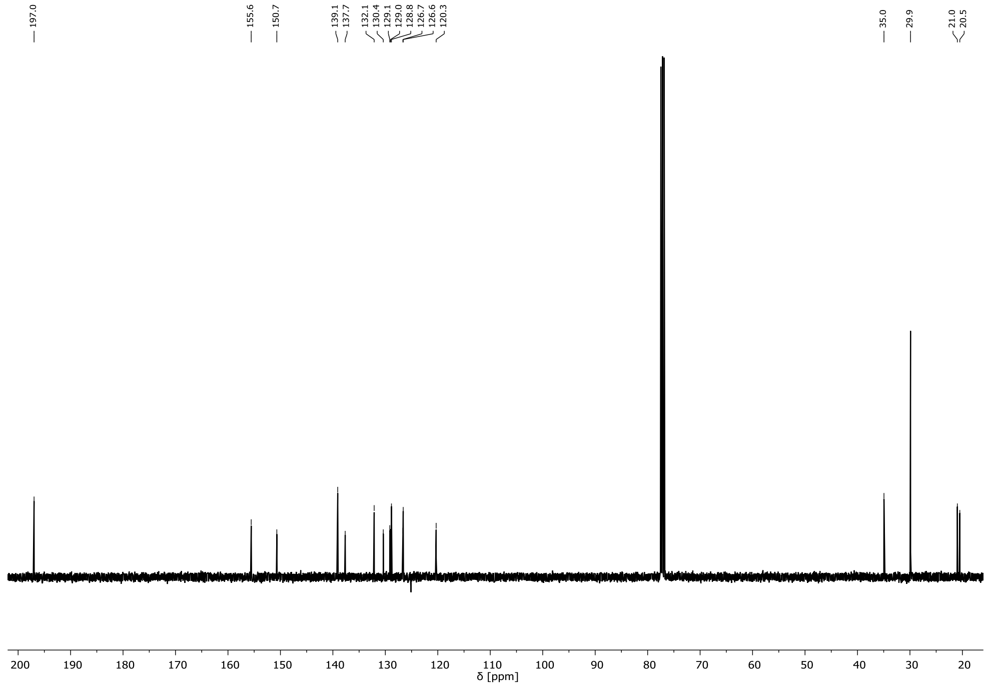

Figure S28. 400 MHz ^{13}C-NMR of **12** in $CDCl_3$.

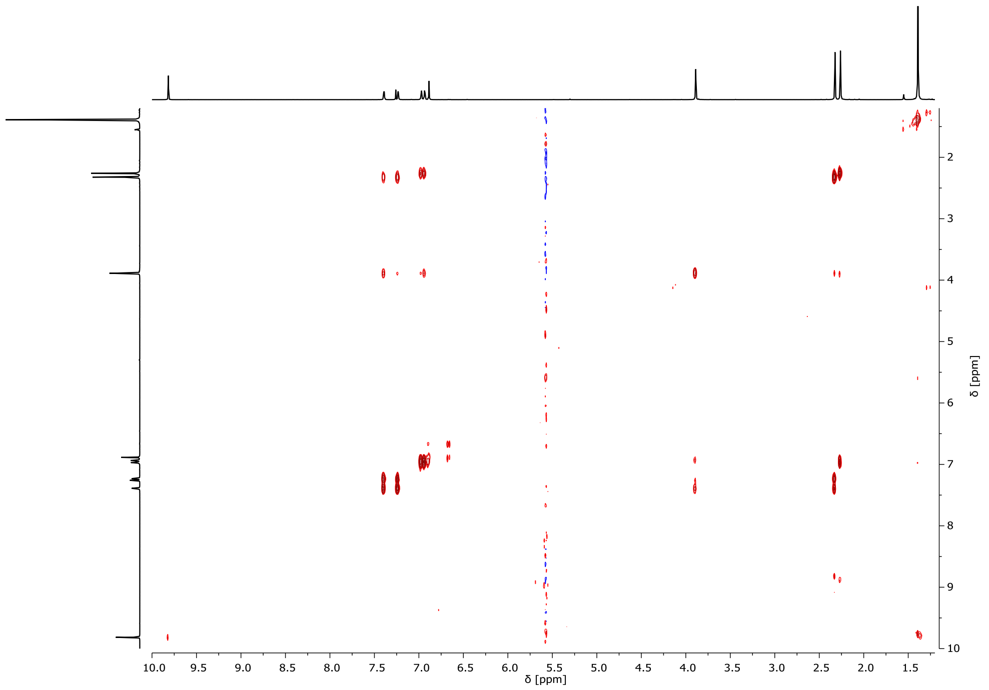

Figure S29. 400 MHz ^{1}H-^{1}H COSY spectrum of **12** in $CDCl_3$.

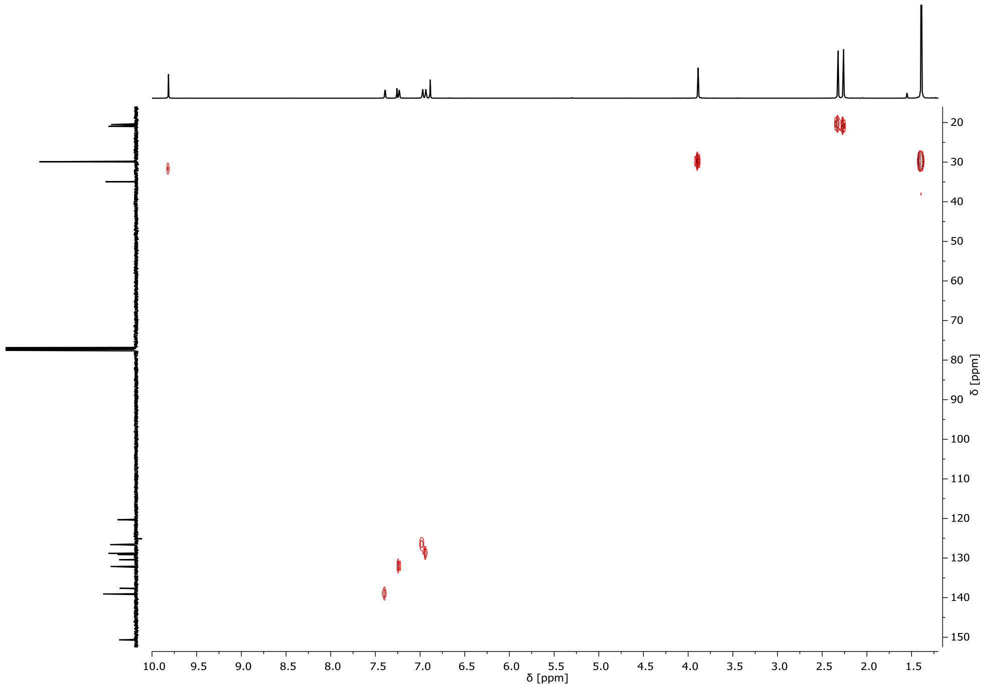

Figure S30. 400 MHz ^{1}H-^{13}C Heteronuclear Single Quantum Coherence (HSQC) spectrum of **12** in $CDCl_3$.

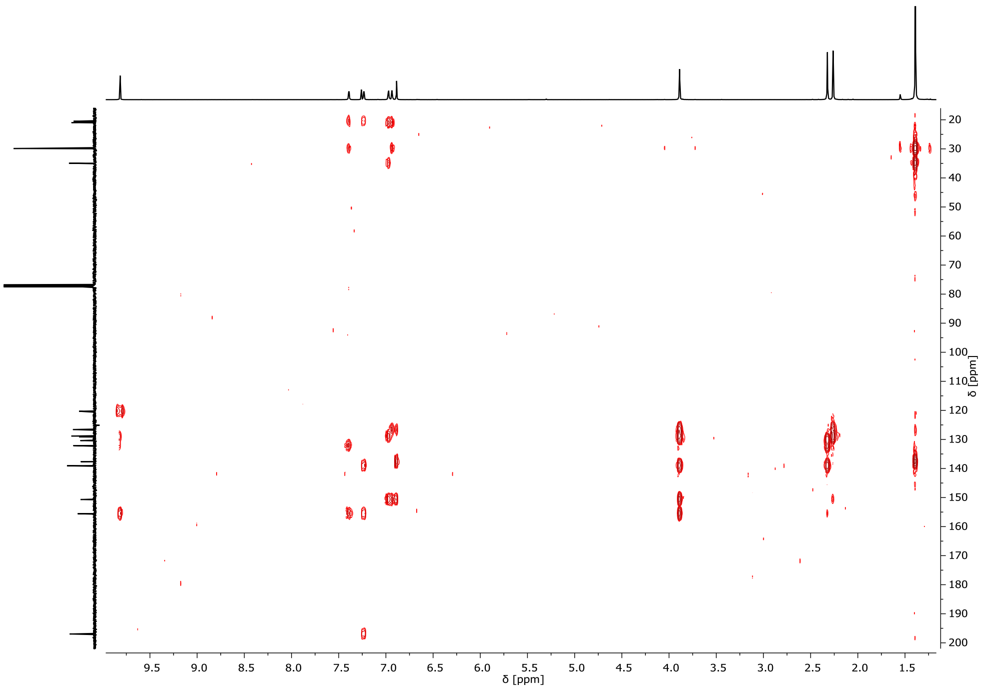

Figure S31. 400 MHz ^{1}H-^{13}C Heteronuclear Multiple Bond Correlation (HMBC) spectrum of **12** in $CDCl_3$.

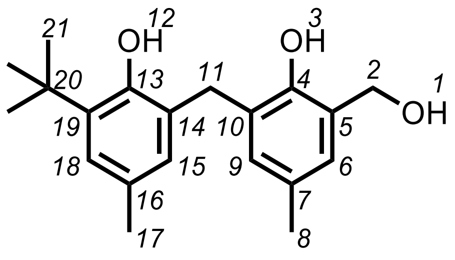

13

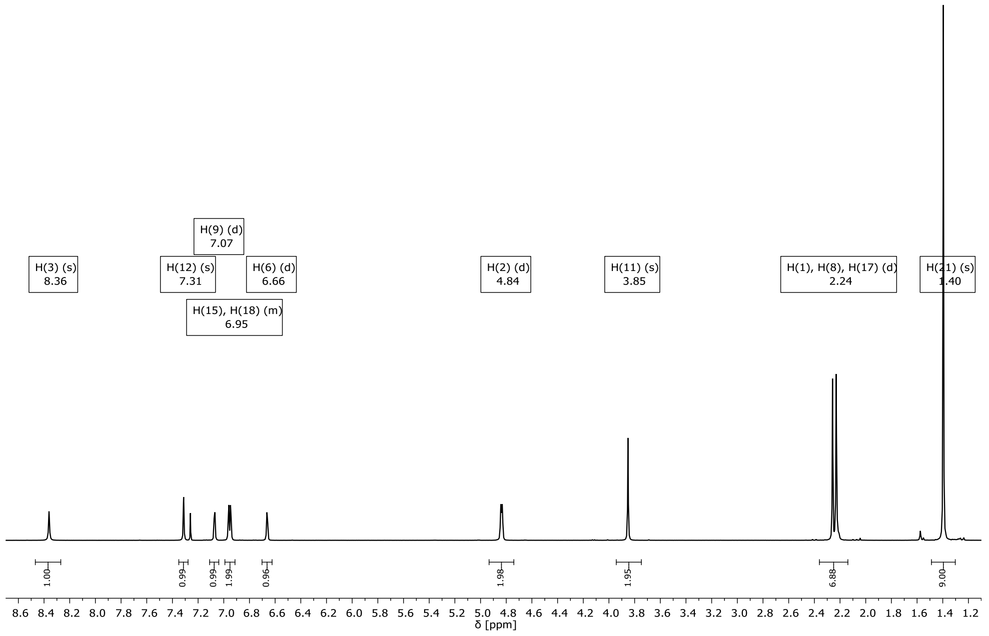

Figure S32. 400 MHz ^{1}H-NMR spectrum of **13** in $CDCl_3$.

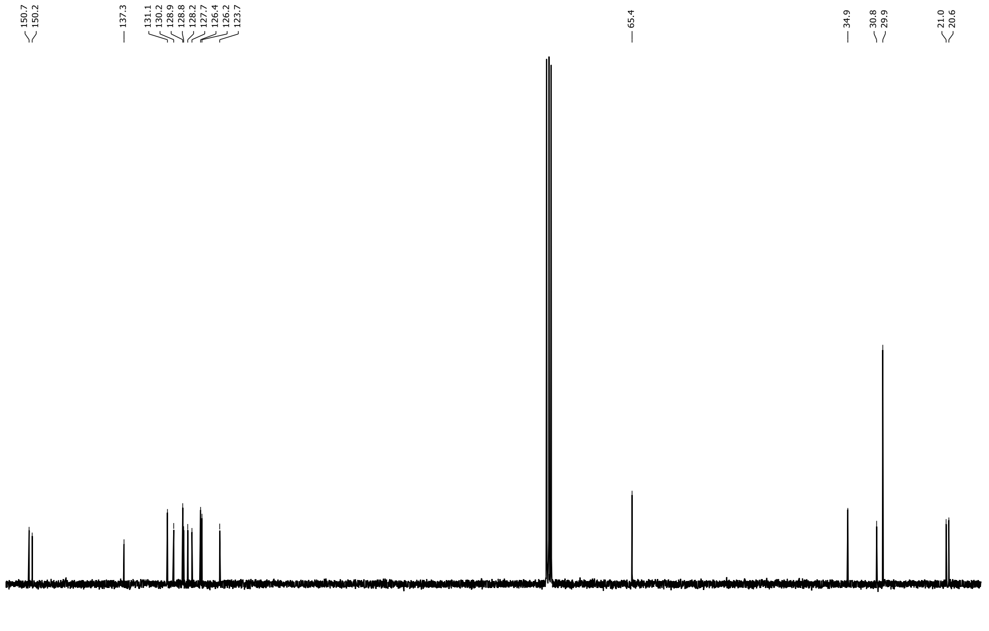

Figure S33. 400 MHz ^{13}C-NMR of **13** in $CDCl_3$.

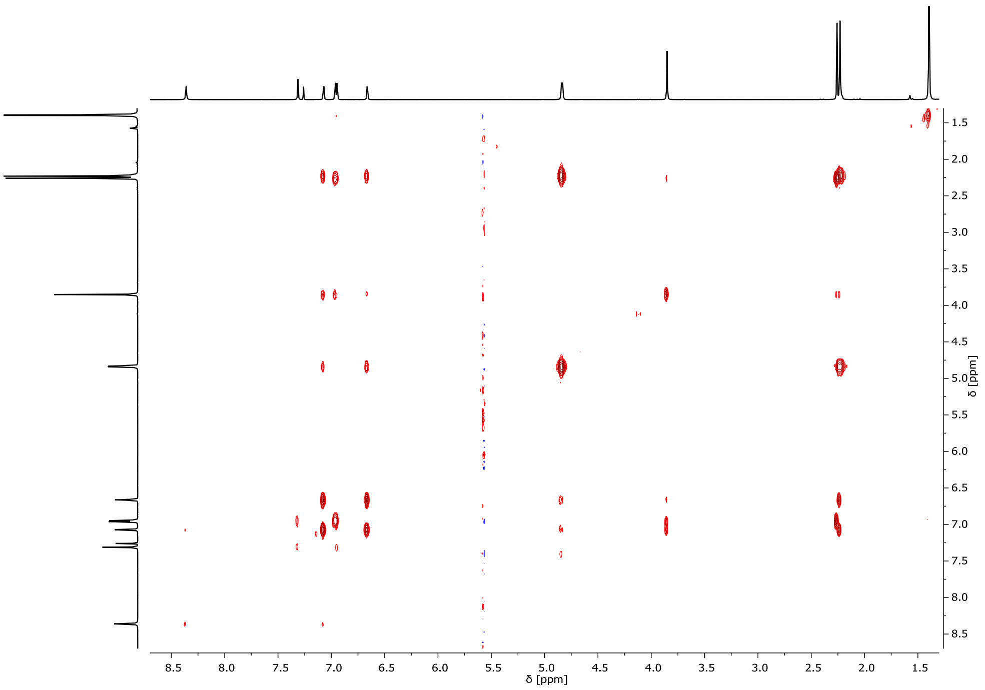

Figure S34. 400 MHz ^{1}H-^{1}H COSY spectrum of **13** in $CDCl_3$.

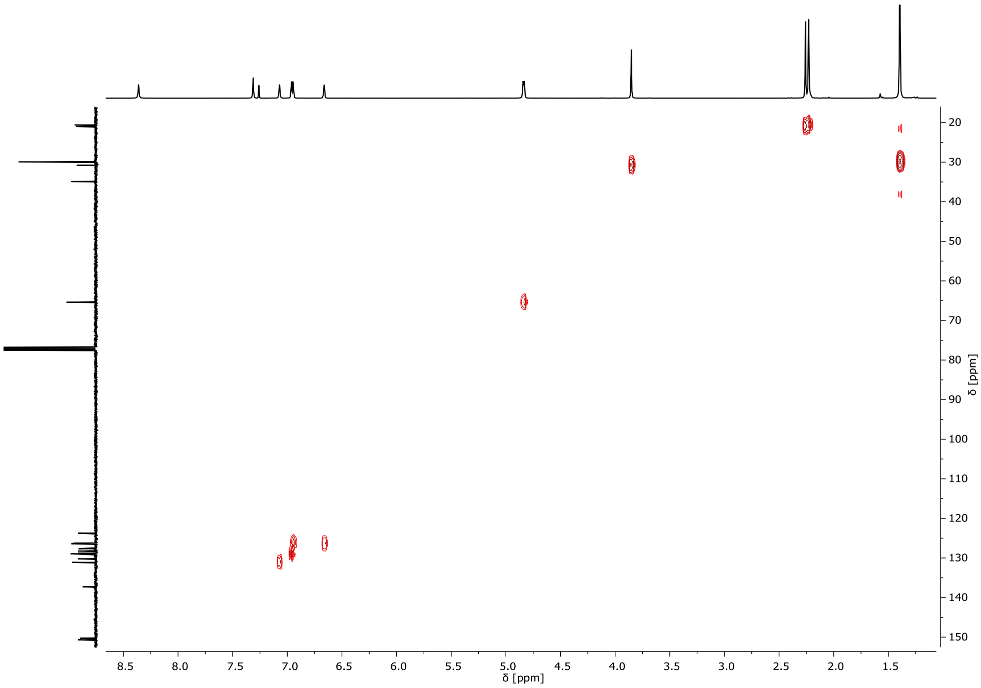

Figure S35. 400 MHz ^{1}H-^{13}C Heteronuclear Single Quantum Coherence (HSQC) spectrum of **13** in $CDCl_3$.

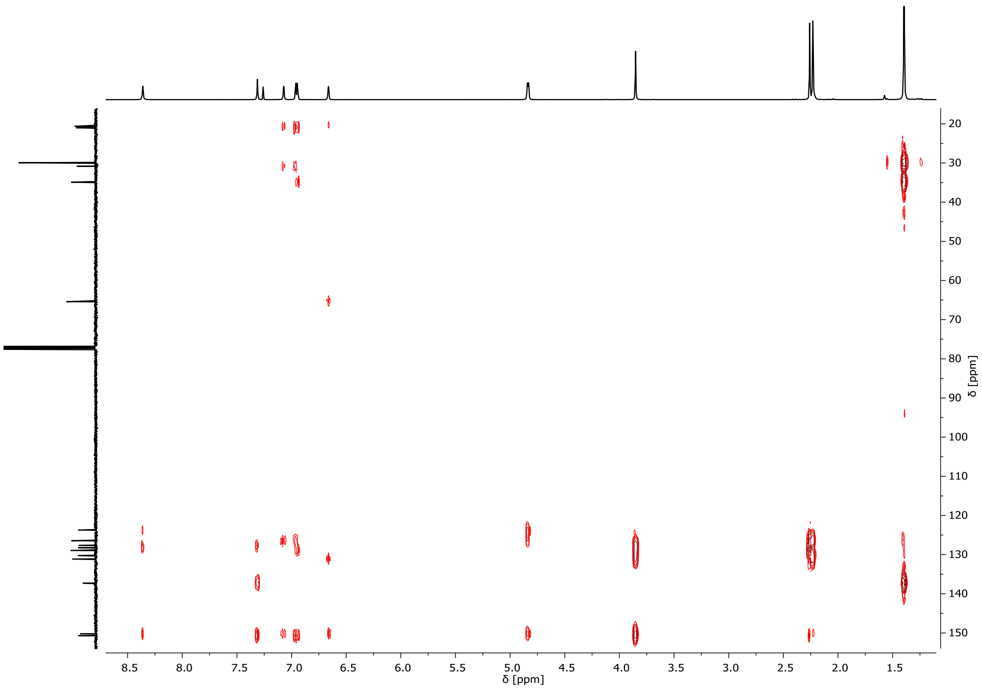

Figure S36. 400 MHz ^{1}H-^{13}C Heteronuclear Multiple Bond Correlation (HMBC) spectrum of **13** in $CDCl_3$.

X-Ray Crystallography

X-ray data were collected on a Bruker D8-QUEST diffractometer, equipped with an Incoatec IμS Cu microsource (λ = 1.5418 Å) and a PHOTON-III detector operating in shutterless mode. The crystal temperature was held at 180(2) K using an Oxford Cryosystems open-flow N_2 Cryostream. The control and processing software was Bruker *APEX4* (ver. 2021.4-0). Structures were solved using SHELXT[9] and refined using SHELXL.[10]

Analysis of **2** and **3·MeCN** was straightforward. Crystals of **3·Quin·$CHCl_3$** were twinned. The diffraction pattern was indexed and integrated as two components related by 180° rotation around the *a* axis. Refinement was carried out using the HKLF-5 format in SHELXL, including all reflections with a contribution from domain 1. The final refined batch scale factor (0.442(1)) suggested that the two crystal domains were present in roughly equal proportions.

For all structure refinements, the H atoms associated with the OH groups were located in the difference Fourier map and their positions were refined freely (without O–H distance restraints), with individual isotropic displacement parameters. In each case, the refinements converged to yield satisfactory models, as illustrated below.

Structure 2 (displacement ellipsoids at 50% probability):

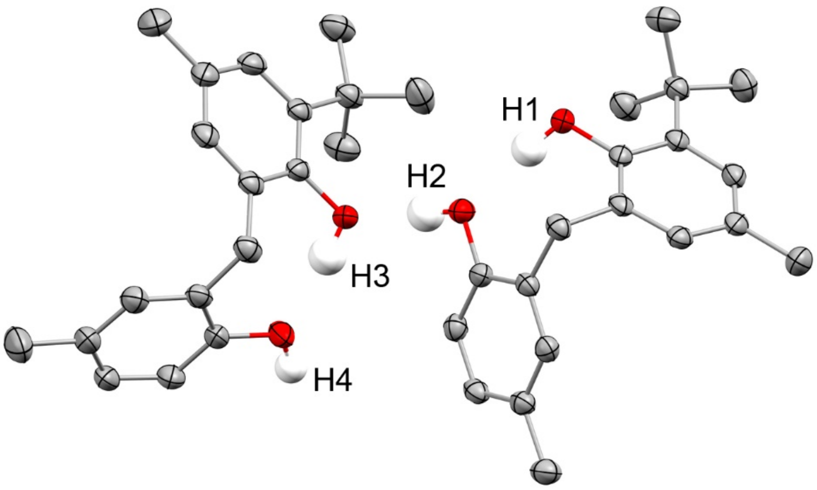

D	H	A	D–H (Å)	H···A (Å)	D···A (Å)	D–H···A (°)
O1A	H1	O2A	0.88(3)	1.87(3)	2.7500(19)	174(3)
O2A	H2	O1B	0.87(3)	1.89(3)	2.760(2)	177(3)
O1B	H3	O2B	0.87(3)	1.86(3)	2.722(2)	173(3)
O2B	H4	O1A	0.88(3)	1.89(3)	2.767(2)	176(2)

Structure 3·MeCN (displacement ellipsoids at 50% probability):

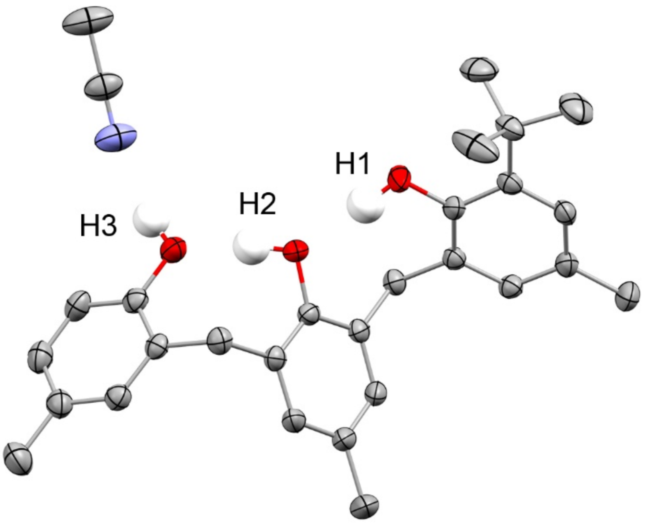

D	H	A	D–H (Å)	H···A (Å)	D···A (Å)	D–H···A (°)
O1	H1	O2	0.91(2)	1.81(2)	2.7094(15)	171(2)
O2	H2	O3	0.95(2)	1.71(2)	2.6430(14)	168(2)
O3	H3	N1	0.87(2)	1.95(2)	2.8018(18)	167(2)

Structure 3·Quin·$CHCl_3$ (displacement ellipsoids at 50% probability):

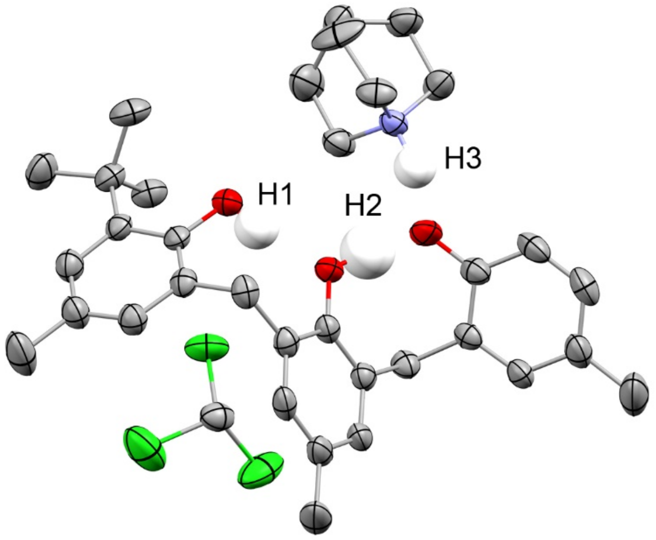

D	H	A	D–H (Å)	H···A (Å)	D···A (Å)	D–H···A (°)
O1	H1	O2	0.81(6)	1.81(6)	2.612(4)	170(6)
O2	H2	O3	1.04(8)	1.39(8)	2.419(4)	168(7)
N1A	H3	O3	1.08(6)	1.56(6)	2.615(4)	162(5)

Table S1. Summary of the crystal and refinement details.

	**2**	**3·MeCN**	**3·Quin·CHCl$_3$**
CCDC number	2189671	2189672	2189670
Cambridge data number	CH_B1_0040	CH_B2_0043	CH_B2_0041
Chemical formula	$C_{19}H_{24}O_2$	$C_{29}H_{35}NO_3$	$C_{35}H_{46}Cl_3NO_3$
Moiety formula	$C_{19}H_{24}O_2$	$C_{27}H_{32}O_3$, C_2H_3N	$C_{27}H_{31}O_3^-$, $C_7H_{14}N^+$, $CHCl_3$
Formula weight	284.38	445.58	635.08
Temperature / K	180(2)	180(2)	180(2)
Crystal system	monoclinic	triclinic	monoclinic
Space group	P 21/c	P –1	P 21/c
a / Å	8.9280(3)	9.0243(5)	9.2560(8)
b / Å	21.8663(7)	9.9731(5)	15.3211(11)
c / Å	16.8001(5)	16.0080(8)	24.385(2)
alpha / °	90	101.915(2)	90
beta / °	90.300(2)	101.650(2)	99.502(4)
gamma / °	90	106.203(2)	90
Unit-cell volume / Å^3	3279.71(18)	1301.02(12)	3410.7(5)
Z	8	2	4
Calc. density / g cm^{-3}	1.152	1.137	1.237
F(000)	1232	480	1352
Radiation type	CuKα	CuKα	CuKα
Absorption coefficient / mm^{-1}	0.569	0.571	2.695
Crystal size / mm^3	0.40 x 0.02 x 0.02	0.20 x 0.15 x 0.06	0.30 x 0.06 x 0.02
2-Theta range / °	6.63-133.23	9.62-133.40	6.84-133.25
Completeness to max 2-theta	0.999	0.989	0.972
No. of reflections measured	60548	18799	11415
No. of independent reflections	5795	4558	5951
R(int)	0.1030	0.0347	0.0859
No. parameters / restraints	405 / 0	317 / 0	453 / 0
Final R1 values (I > 2σ (I))	0.0459	0.0421	0.0552
Final wR(F^2) values (all data)	0.1230	0.1150	0.1423
Goodness-of-fit on F^2	1.014	1.060	1.052
Largest difference peak & hole / e Å^{-3}	0.241, -0.230	0.245, -0.235	0.248, -0.286

Dispersion-corrected density functional theory (DFT-D) calculations for the crystal structures

To support the conclusions on the location of the H atoms in the crystal structures, particularly for the twinned structure of **3·Quin·CHCl$_3$**, the crystal structures containing **3** and **4** were energy minimised using periodic dispersion-corrected density functional theory (DFT-D). The calculations were carried out using CASTEP[11] via the interface in Materials Studio (Accelrys, 2012). The PBE exchange-correlation functional was applied,[12] with a dispersion correction according to Grimme.[13] The plane-wave basis-set cut-off was set to 340 eV and all other parameters were set to the "Fine" defaults in Materials Studio. Unit-cell parameters were constrained in each case to those from the reported crystal structure and the space-group symmetry was imposed. For **4**, the starting model was taken from the structure available in the Cambridge Structural Database (CUPDUM[14]). Prior to energy minimisation, the positions of all H atoms were normalised

using the default settings in Mercury[15] to produce starting positions close to nuclear positions. For **3·Quin·CHCl₃**, in which quinuclidine is modelled in two orientations indicative of rotational disorder, only the major component was retained. The energy minimised structures were compared to the starting structures using the method described by van de Streek and Neumann.[16] In each case, the shifts of the non-H atoms on minimisation are in line with expectations for correct crystal structures:

Cartesian displacement (Å)	**3·MeCN**	**3·Quin·CHCl$_3$**	**4**
All atom RMS	0.142	0.174	0.155
All atom maximum	0.356 [H29C]	0.426 [H2]	0.511 [H29]
Non-H RMS	0.113	0.147	0.071
Non-H maximum	0.230 [C19]	0.281 [C11]	0.130 [C33]

To confirm the salt structure as the energy minimum for **3·Quin·CHCl$_3$**, the minimisation was carried out using two different starting models: (1) with H3 on N3A, as obtained from the X-ray refinement; (2) with H3 transferred to atom O3 to form a neutral co-crystal. For (2), atom H3 migrated during the optimisation and both starting models converged to the same result in which H3 is bonded to N3A. Atom H2 converges at a position close to half-way between O2 and O3 (closer to O3; see below). The same result is **not** seen in the minimised structures of **3·MeCN** or **4**, which include clearly-defined OH groups with O–H distances in the range 1.00–1.02 Å.

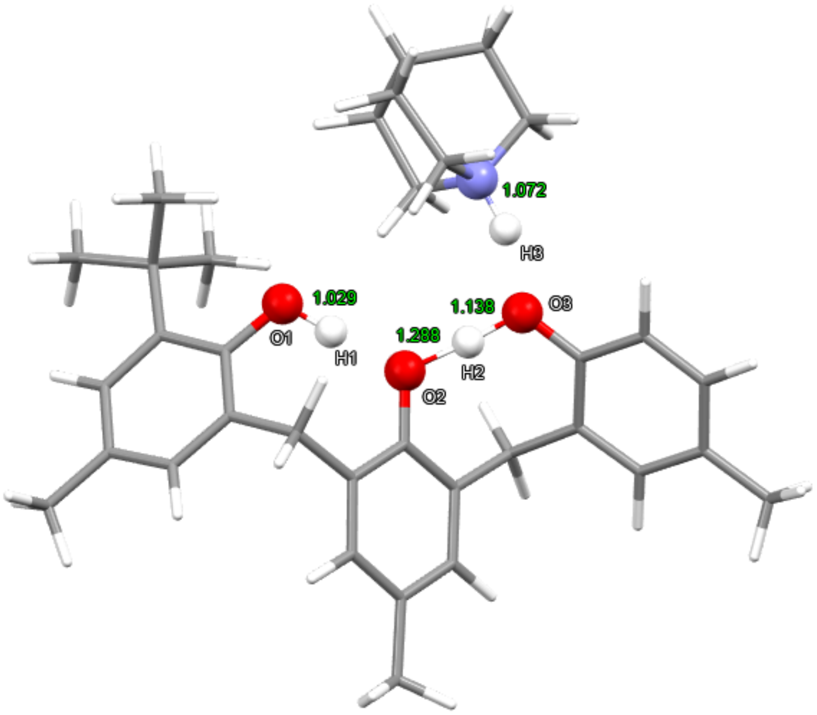

NMR Experiments

^{1}H-NMR Titration – General Procedure

A diluted solution of the host (5 mL) in *n*-octane is prepared from the stock solution of the receptor in *n*-octane. 600 μL of the host solution is titrated with a solution of the guest (G) containing also the host at the same concentration in *n*-octane. A ^{1}H-NMR spectrum with WET solvent suppression is recorded for every point of the titration on a 500 MHz spectrometer. The chemical shifts of the protons of the host are monitored upon addition of various concentrations of guest. The observed chemical shift (δ_{obs}, ppm, Equation 1) is a weighted average of the chemical shifts of the free host (δ_H, ppm) and the host-guest complex ($\delta_{H \cdot G}$, ppm):

$$\delta_{obs} = \delta_H X_H + \delta_{H \cdot G} X_{H \cdot G} \quad (1)$$

where X_H and $X_{H \cdot G}$ are the mole fractions of H ($X_H = [H]/[H]_0$) and of H·G ($X_{H \cdot G} = [H \cdot G]/[H]_0$), respectively. $[H]_0$ is the initial concentration of H ($[H]_0$=[H·G]+ [H]) and $[G]_0$ is the initial concentration of G ($[G]_0$=[H·G]+ [G]). Since $X_H = 1 - X_{H \cdot G}$, equation (1) can be rearranged:

$$\delta_{obs} = \delta_H (1 - X_{H \cdot G}) + \delta_{H \cdot G} X_{H \cdot G} \quad (2)$$

$$\frac{\delta_{obs} - \delta_H}{\delta_{HG} - \delta_H} = X_{H \cdot G} = \frac{[HG]}{[H]_0} \quad (3)$$

Given that the association constant K_a for the 1:1 H·G complex is [H·G]/[H][G] and $[H]_0$=[H·G]+ [H], equation (4) can be written as

$$\frac{\delta_{obs} - \delta_H}{\delta_{HG} - \delta_H} = \frac{K_a[G]}{1 + K_a[G]} \quad (4)$$

and [G] can be determined using equation (5) by making iteratively guesses of K_a and solving for [G] until the theoretical isotherm matches the experimental data:

$$K_a[G]^2 + (K_a[H]_0 - K_a[G]_0 + 1)[G] - [G]_0 = 0 \quad (5)$$

A Microsoft Excel spreadsheet with purpose-written VBA macros was used to solve equations (4) and (5).[17]

^{1}H-NMR Dilution – General Procedure

A diluted solution of the host (2 mL) in *n*-octane is prepared from the stock solution of the receptor in *n*-octane. Increasing volumes of the host solution were added to 600 μL of *n*-octane. A ^{1}H-NMR spectrum with WET solvent suppression is recorded after each addition.

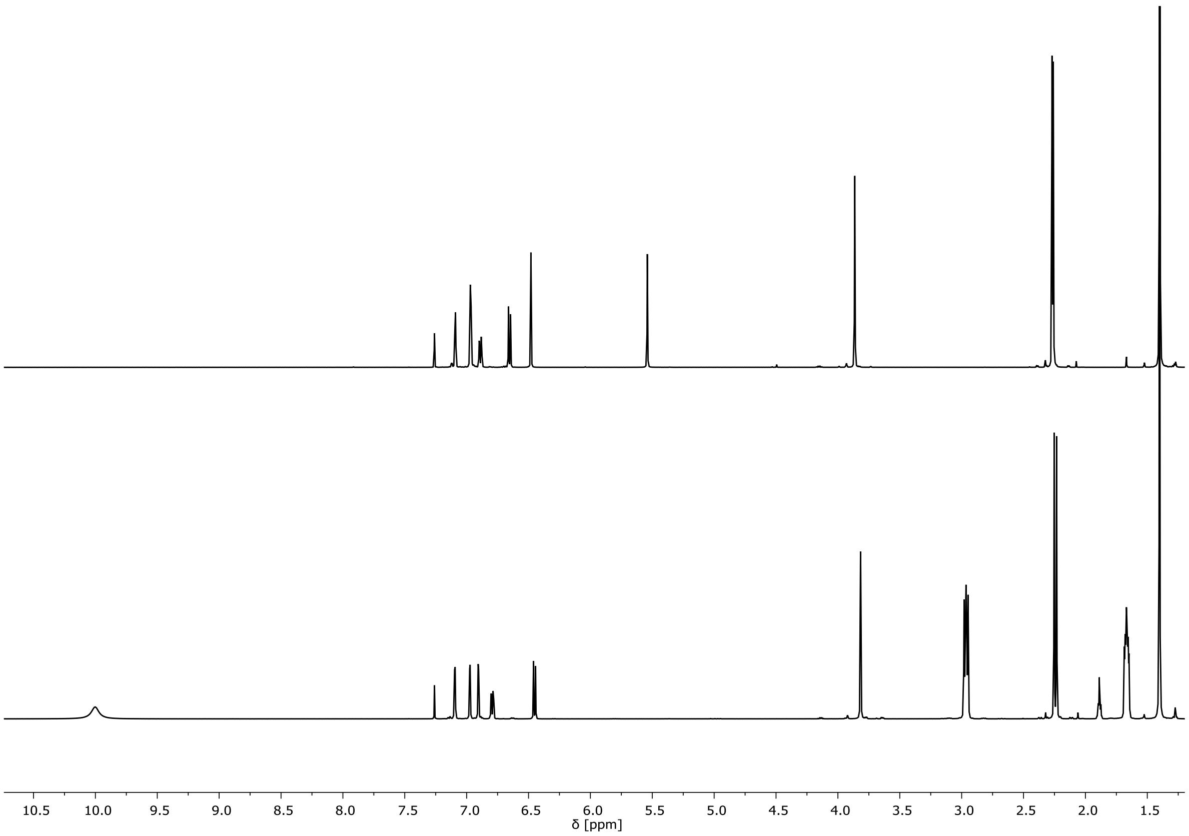

Figure S37. 500 MHz ^{1}H-NMR spectrum of receptor **2** (on top), and of **2**·quinuclidine (1:1.1, at the bottom) in $CDCl_3$.

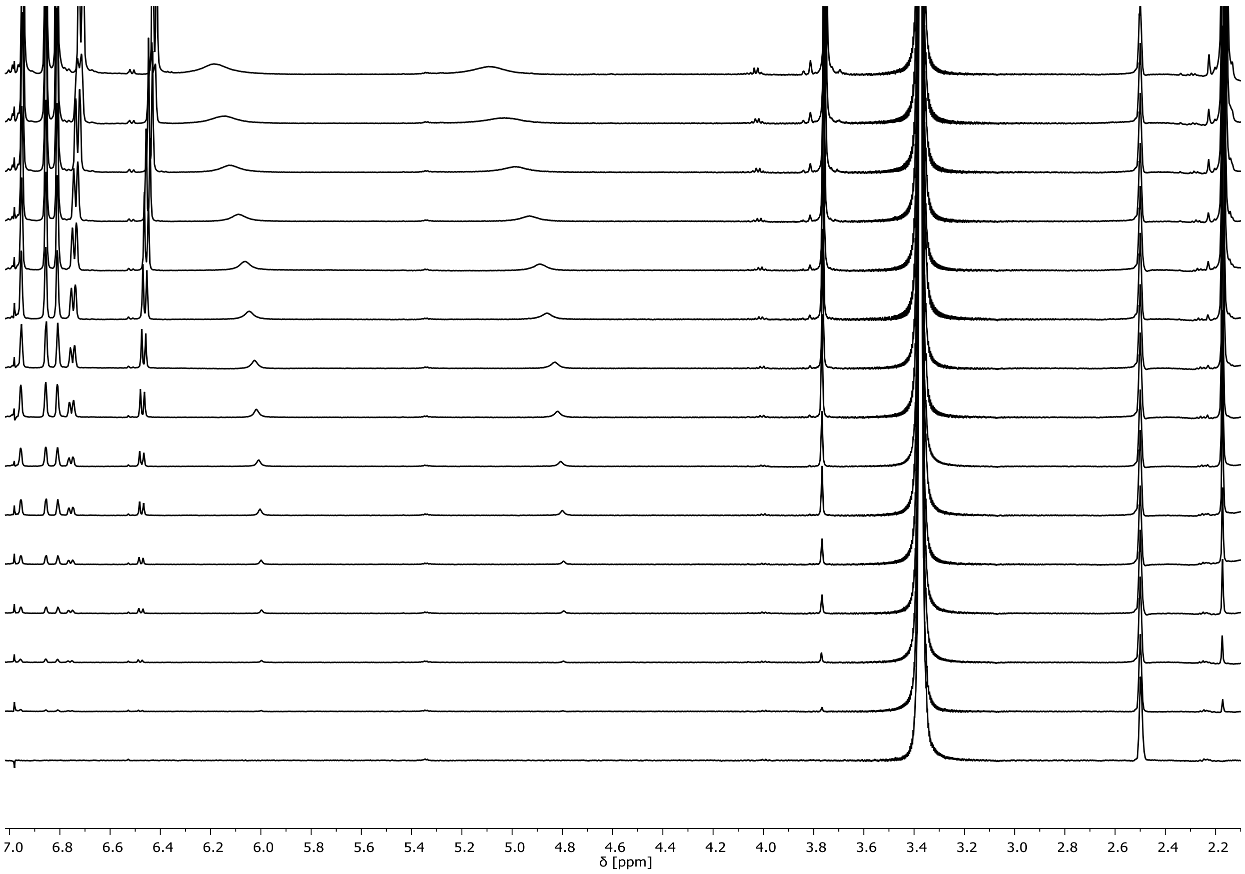

Figure S38. NMR dilution of receptor **2** in *n*-octane (500 MHz ^{1}H-NMR spectra with WET solvent suppression, at the following concentrations of **2** (from bottom to top) 0 mM, 0.046 mM, 0.092 mM, 0.183 mM, 0.273 mM, 0.447 mM, 0.616 mM, 0.939 mM, 1.242 mM, 1.798 mM, 2.295 mM, 3.147 mM, 4.439 mM, 5.749 mM, 7.269 mM; artifact at 6.98 ppm; H_2O at 3.37 ppm; DMSO at 2.50 ppm).

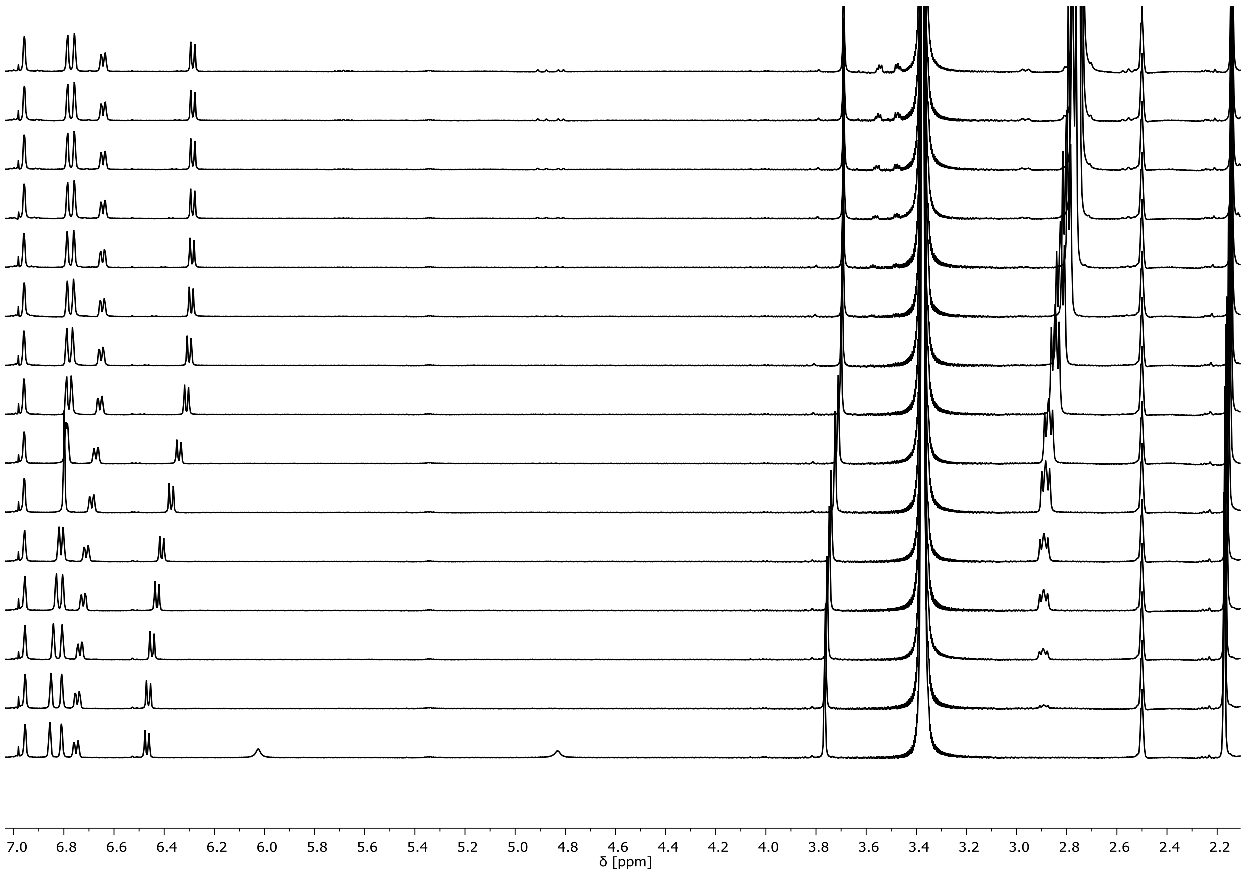

Figure S39. 500 MHz ^{1}H-NMR titration of receptor **2** (1.224 mM) with quinuclidine in *n*-octane (500 MHz ^{1}H-NMR spectra with WET solvent suppression, artifact at 6.98 ppm; H_2O at 3.37 ppm; DMSO at 2.50 ppm).

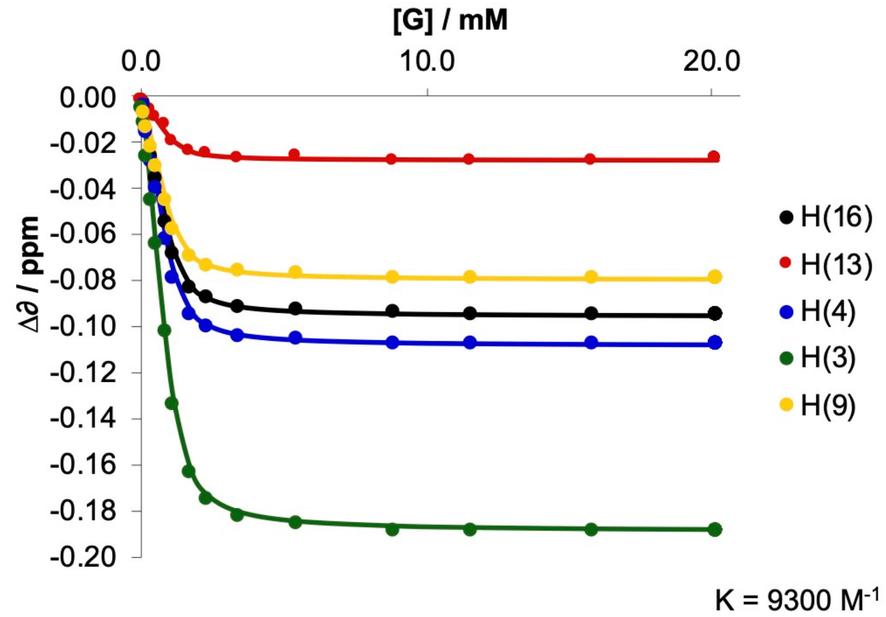

Figure S40. Fitting of the data from the NMR titration of receptor **2** with quinuclidine in *n*-octane (Figure S8).

NOESY Experiments[18,19]

(a)

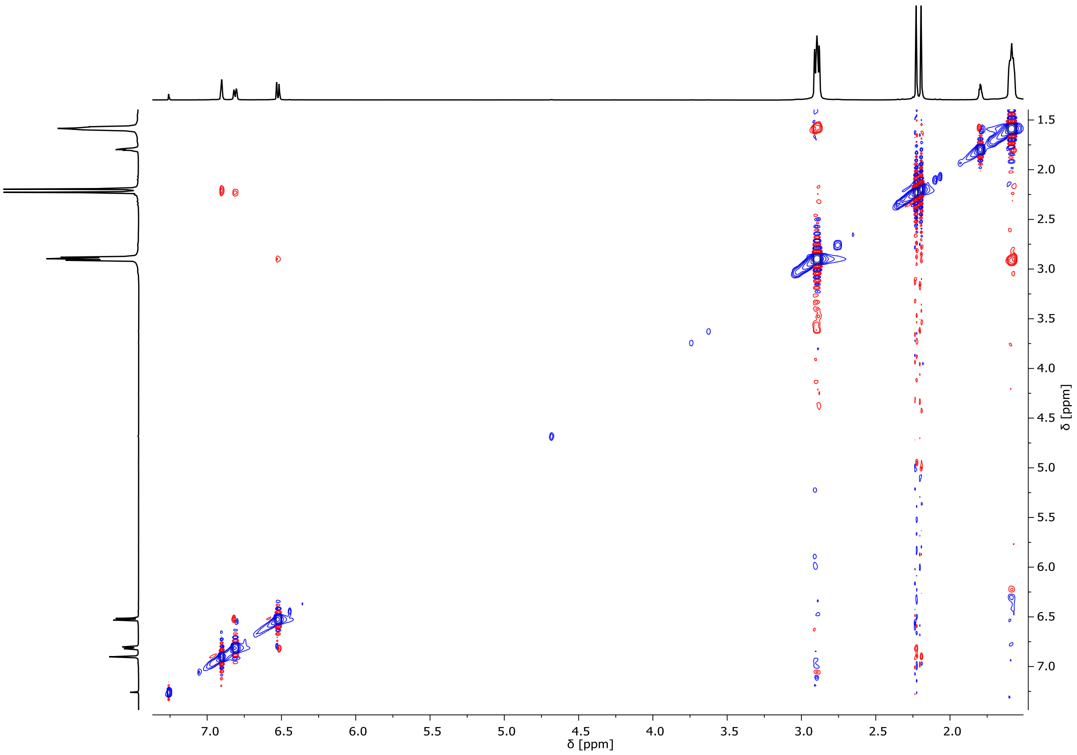

(b)

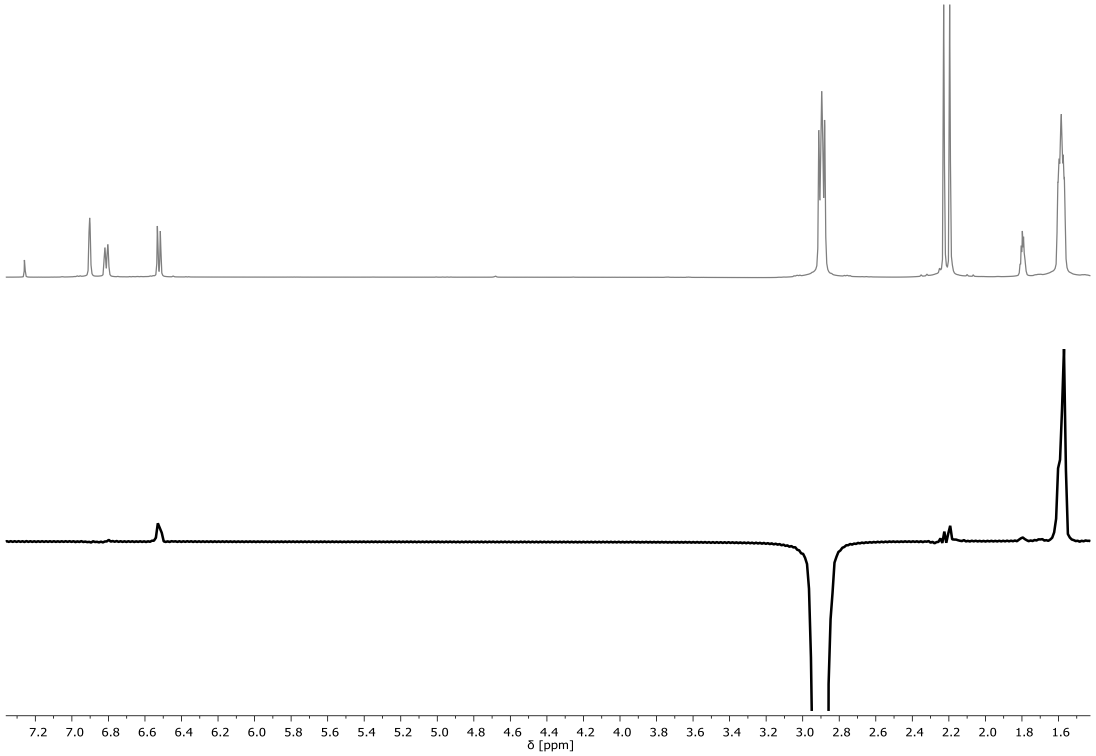

Figure S41. (a) 500 MHz NOESY experiment of **1** and quinuclidine (1:1.3) in $CDCl_3$, and (b) 500 MHz ^1H-NMR spectrum of **1** and quinuclidine (1:1.3) in $CDCl_3$ (on the top) and ^1H-NMR spectrum extracted from the NOESY experiment at 2.90 ppm.

(a)

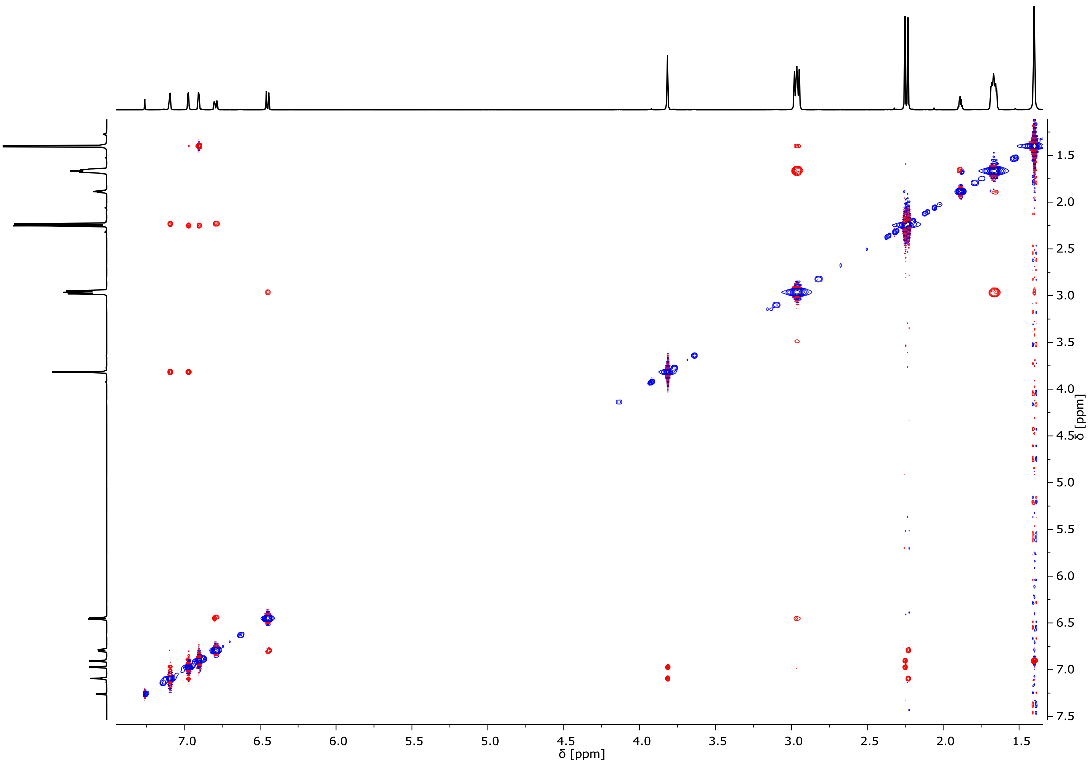

(b)

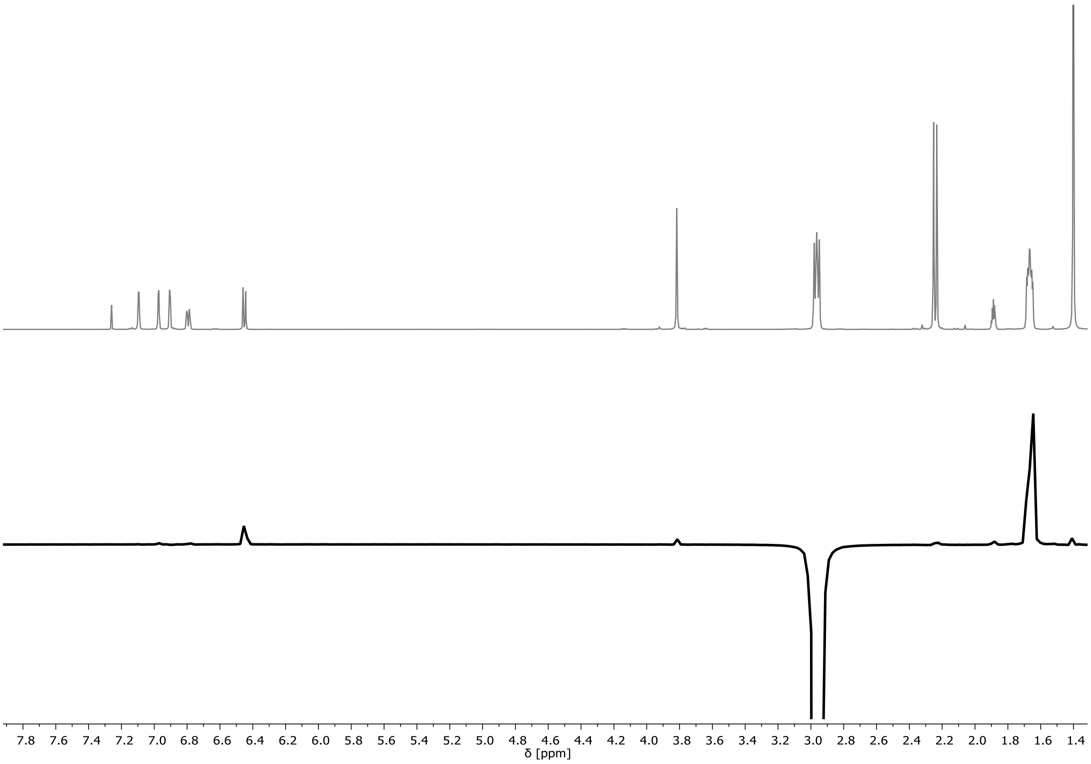

Figure S42. (a) 500 MHz NOESY experiment of **2** and quinuclidine (1:1.1) in $CDCl_3$, and (b) 500 MHz ^1H-NMR spectrum of **2** and quinuclidine (1:1.1) in $CDCl_3$ (on the top) and ^1H-NMR spectrum extracted from the NOESY experiment at 2.96 ppm.

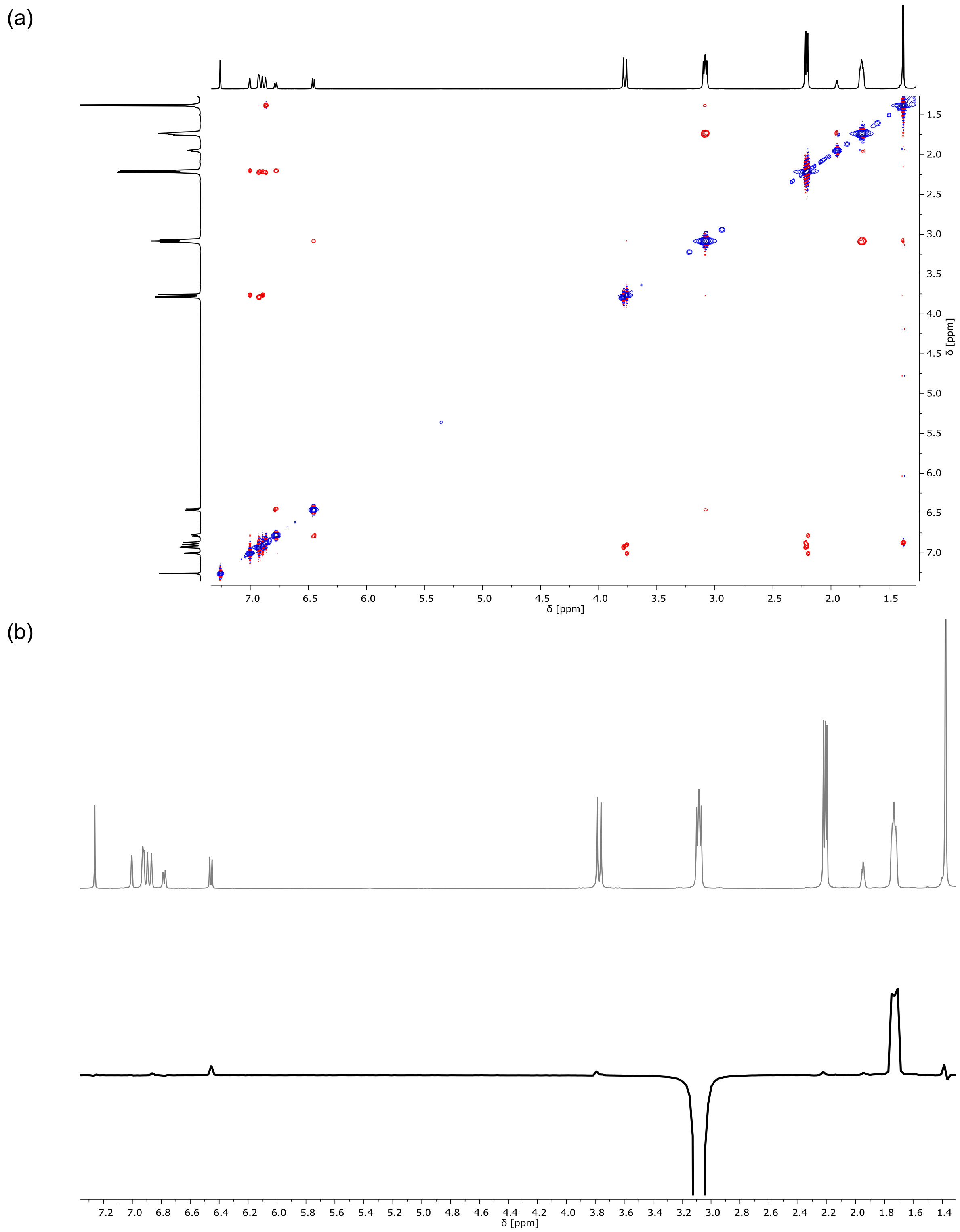

Figure S43. (a) 500 MHz NOESY experiment of **3** and quinuclidine (1:1.1) in $CDCl_3$, and (b) 500 MHz ^{1}H-NMR spectrum of **3** and quinuclidine (1:1.1) in $CDCl_3$ (on the top) and ^{1}H-NMR spectrum extracted from the NOESY experiment at 3.08 ppm.

(a)

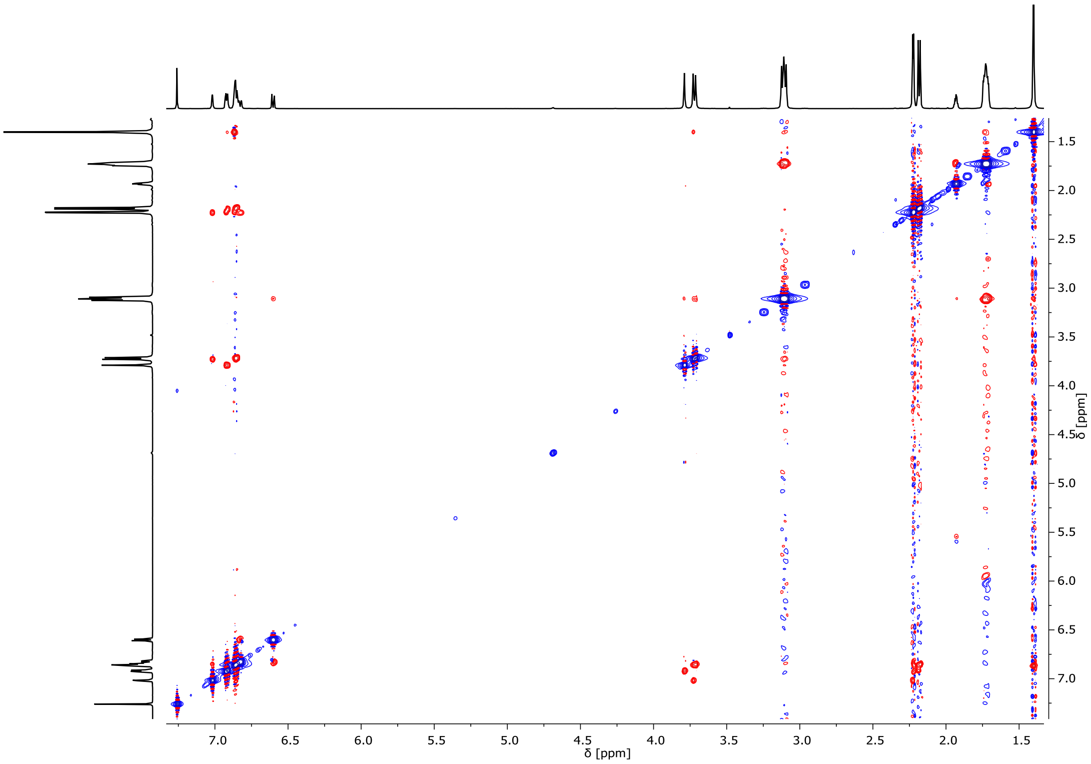

(b)

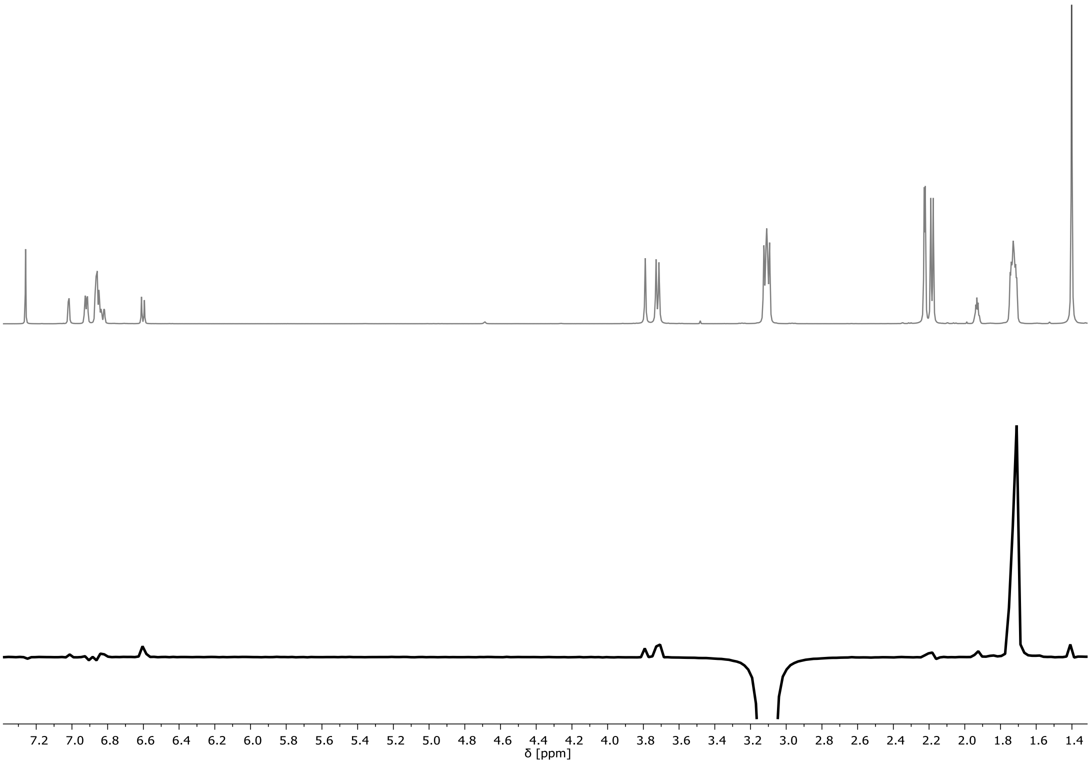

Figure S44. (a) 500 MHz NOESY experiment of **4** and quinuclidine (1:1.6) in $CDCl_3$, and (b) 500 MHz ^1H-NMR spectrum of **4** and quinuclidine (1:1.6) in $CDCl_3$ (on the top) and ^1H-NMR spectrum extracted from the NOESY experiment at 3.11 ppm.

UV-Vis experiments

UV-Vis Titration – General Procedure

A diluted solution of the host (5 mL) in *n*-octane is prepared from the stock solution of the receptor in *n*-octane. 2 mL of the host solution is titrated with a solution of the guest containing also the host at the same concentration in *n*-octane. A UV-Vis spectrum is recorded for every point of the titration. Analogous to the NMR titrations, equation (6) can be written as

$$\frac{A_{obs}-A_0}{A_f-A_0} = \frac{K_a[G]}{1+K_a[G]} \qquad (6)$$

where A_{obs} (a.u.) is the observed absorbance, A_0 (a.u.) is the initial absorbance, A_f (a.u.) is the final absorbance, K_a is the association constant and [G] is the concentration of free guest. [G] can be determined using equation (7) by making iteratively guesses of K_a and solving for [G] until the theoretical isotherm matches the experimental data:

$$K_a[G]^2+ (K_a[H]_0- K_a[G]_0+ 1)[G]- [G]_0=0 \qquad (7)$$

where $[H]_0$ and $[G]_0$ are the total concentrations of the host and guest, respectively.
A Microsoft Excel spreadsheet with purpose-written VBA macros was used to solve equations (6) and (7) fitting the experimentally measured absorbance at specified wavelengths.[17]

Each titration was repeated three times, fitted with equations (6) and (7), and an average value of the association constant along with its standard error (with 95% confidence) is reported in table S2.

UV-Vis Dilution – General Procedure

A diluted solution of the host (5 mL) in *n*-octane is prepared from the stock solution of the receptor in *n*-octane. Increasing volumes of *n*-octane were added to 2 mL or 1.5 mL of the host solution. A UV-Vis spectrum is recorded after each addition.

**Donor**	**Acceptor**		
	**Quin**	**$HeptNH_2$**	**Oct_3N**
**1**	$(1.8 \pm 0.1)\times10^2$	46 ± 9	< 5
**2**	$(9.1 \pm 0.3)\times10^3$	$(1.1 \pm 0.1)\times10^3$	$(3.3 \pm 0.1)\times10^2$
**3**	$(4.5 \pm 0.5)\times10^4$	$(3.3 \pm 0.2)\times10^3$	$(6.7 \pm 0.3)\times10^2$
**4**	$(3.9 \pm 0.5)\times10^4$	$(3.9 \pm 0.2)\times10^3$	$(7.6 \pm 0.2)\times10^2$
**5**	20 ± 6	< 5	< 5
**6**	36 ± 2	9 ± 1	< 5

Table S2. Association constants (M^{-1}) for formation of 1:1 complexes measured by UV-Vis spectroscopy titrations in n-octane at 298 K. Errors are the standard error of the mean of three independent experiments.

Molecule 1

Dilution Experiment of 1 in *n*-octane

(a)

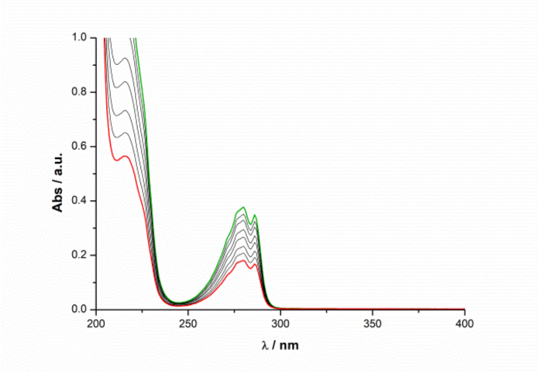

(b)

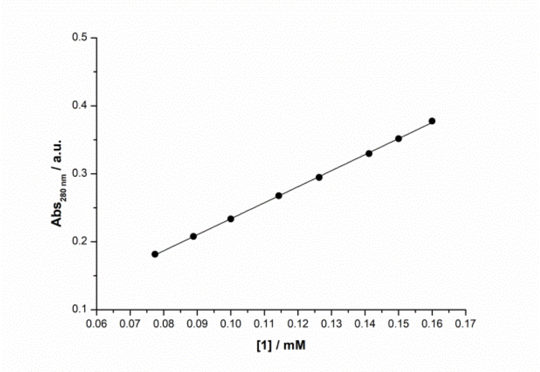

Figure S45. (a) UV-Vis spectra of 2,4-dimethylphenol **1** in *n*-octane at decreasing concentrations (from 0.160 mM in green to 0.077 mM in red), and (b) plot of the absorbance of **1** at 280 nm versus the concentration of **1** and its linear fitting.

Titrations of 1 with quinuclidine in *n*-octane

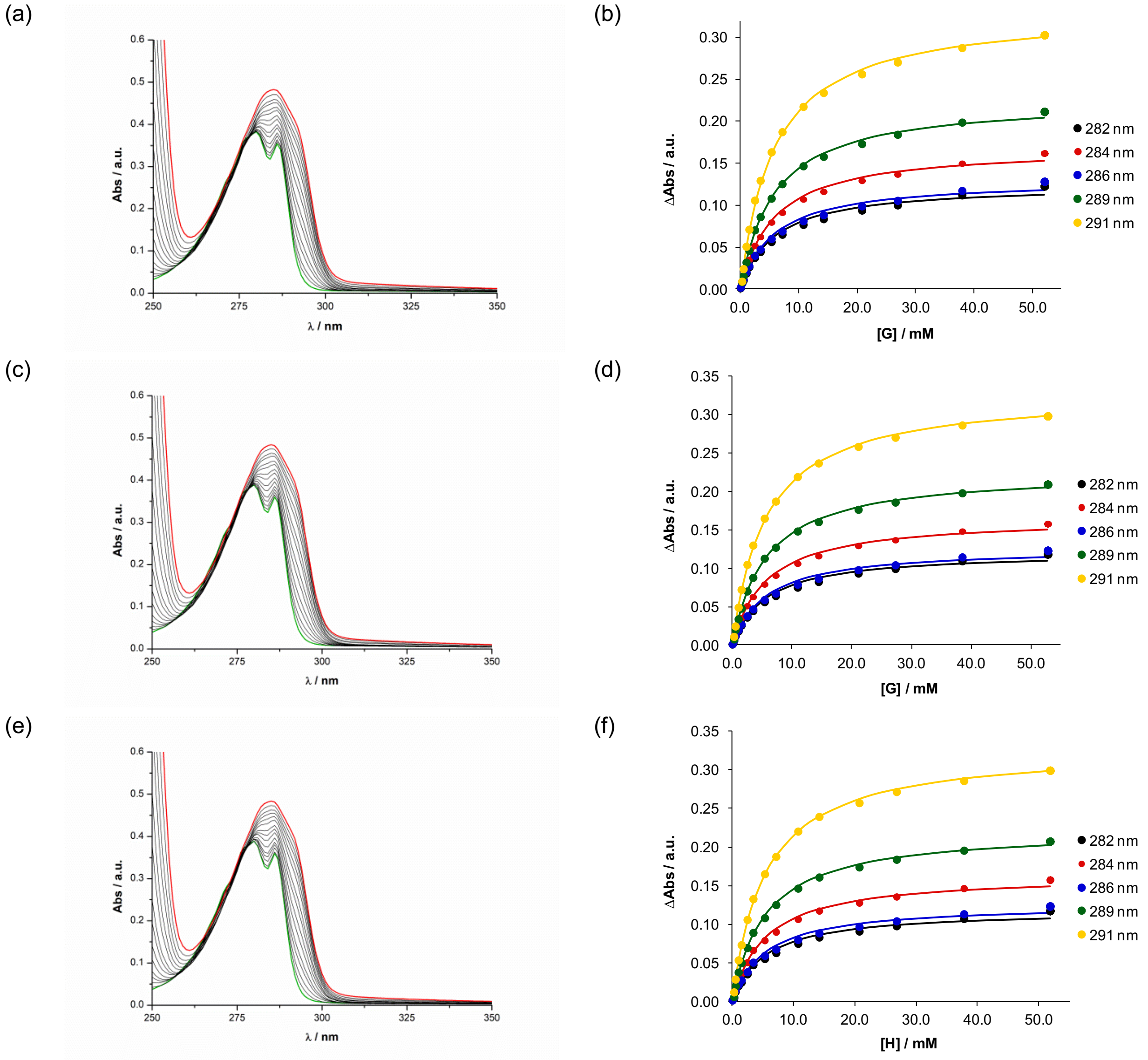

Figure S46. (a, c, e) UV-Vis spectra of 2,4-dimethylphenol **1** (0.160 mM in green) in *n*-octane at increasing concentrations of quinuclidine (from green to red), and (b, d, f) plot of the absorbance of **1** at 282 nm, 284 nm, 286 nm, 289 nm, 291 nm versus the concentration of **1** and its fittings to a 1:1 binding model.

Titrations of 1 with *n*-heptylamine in *n*-octane

(a)

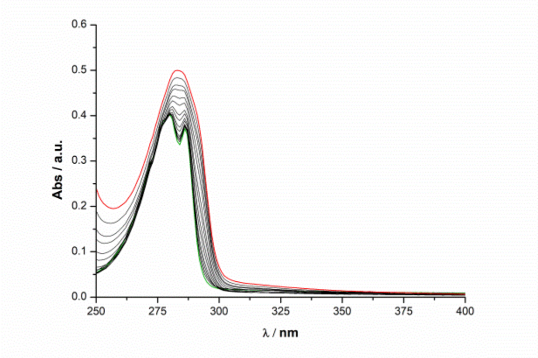

(b)

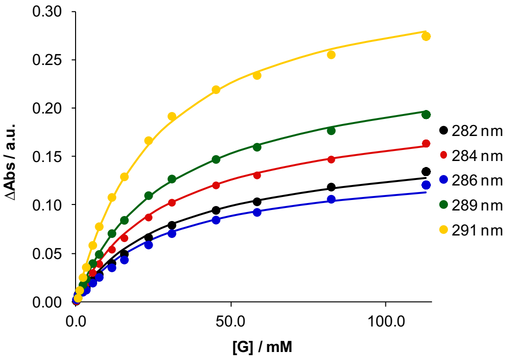

(c)

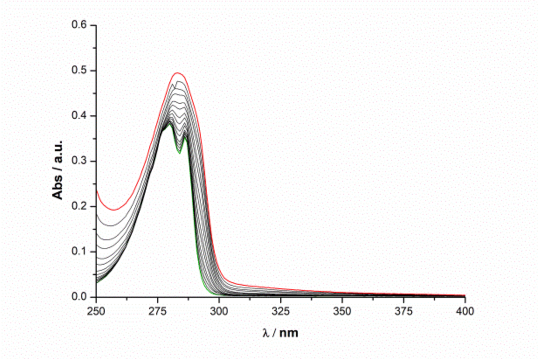

(d)

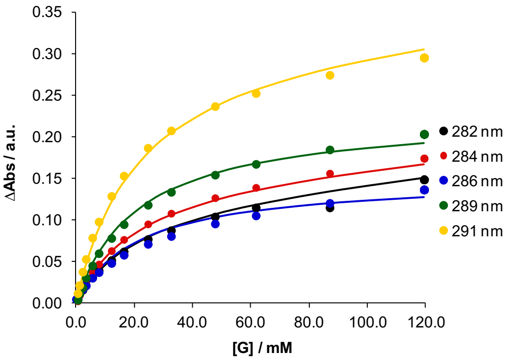

(e)

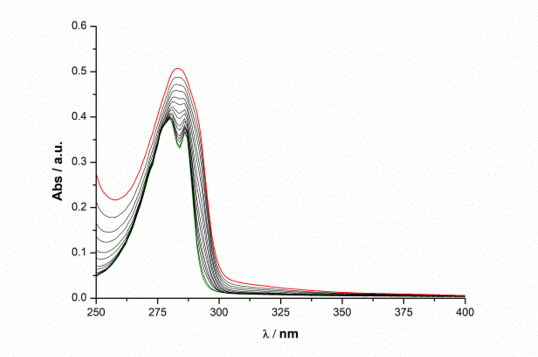

(f)

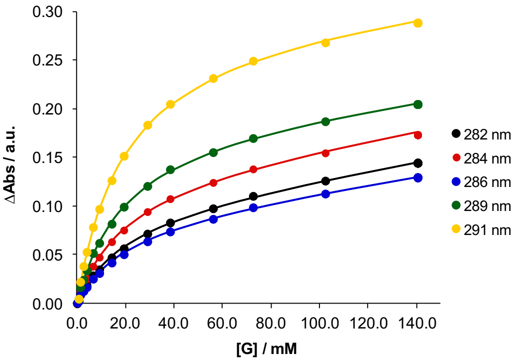

Figure S47. (a, c, e) UV-Vis spectra of **1** (0.160 mM) in *n*-octane at increasing concentrations of *n*-heptylamine (from green to red), and (b, d, f) plot of the absorbance of **1** at 282 nm, 284 nm, 286 nm, 289 nm, 291 nm versus the concentration of **1** and its fittings to a 1:1+non-specific binding model.

Titration of 1 with tri-*n*-octylamine in *n*-octane

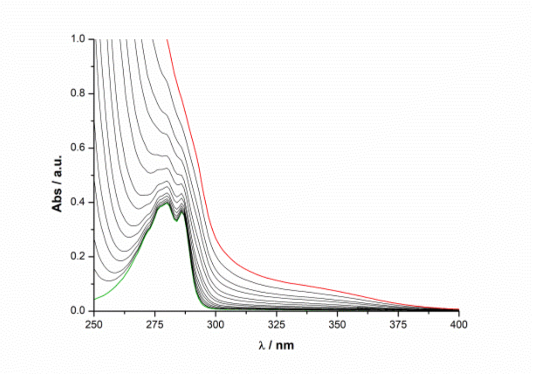

Figure S48. (a) UV-Vis spectra of **1** (0.160 mM) in *n*-octane at increasing concentrations of tri-*n*-octylamine (from green to red).

Molecule 2

Dilution Experiment of 2 in *n*-octane

(a)

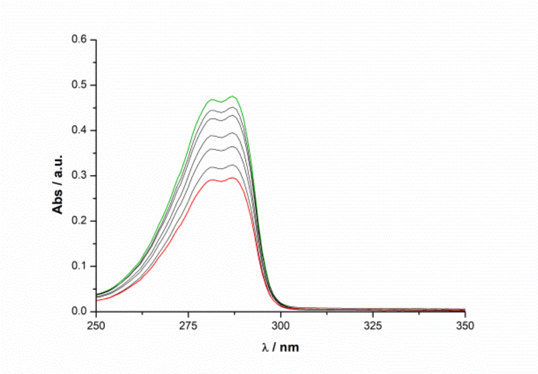

(b)

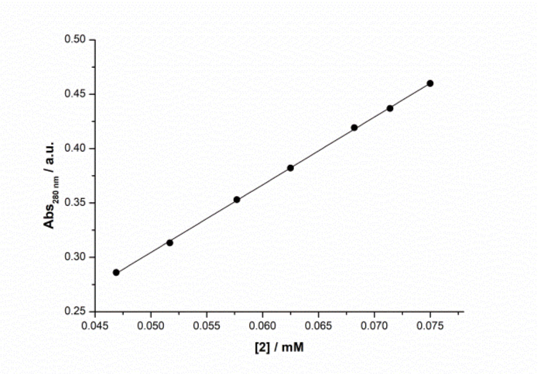

Figure S49. (a) UV-Vis spectra of **2** in *n*-octane at decreasing concentrations (from 0.075 mM in green to 0.047 mM in red), and (b) plot of the absorbance of **2** at 280 nm versus the concentration of **2** and its linear fitting.

Titrations of 2 with quinuclidine in *n*-octane

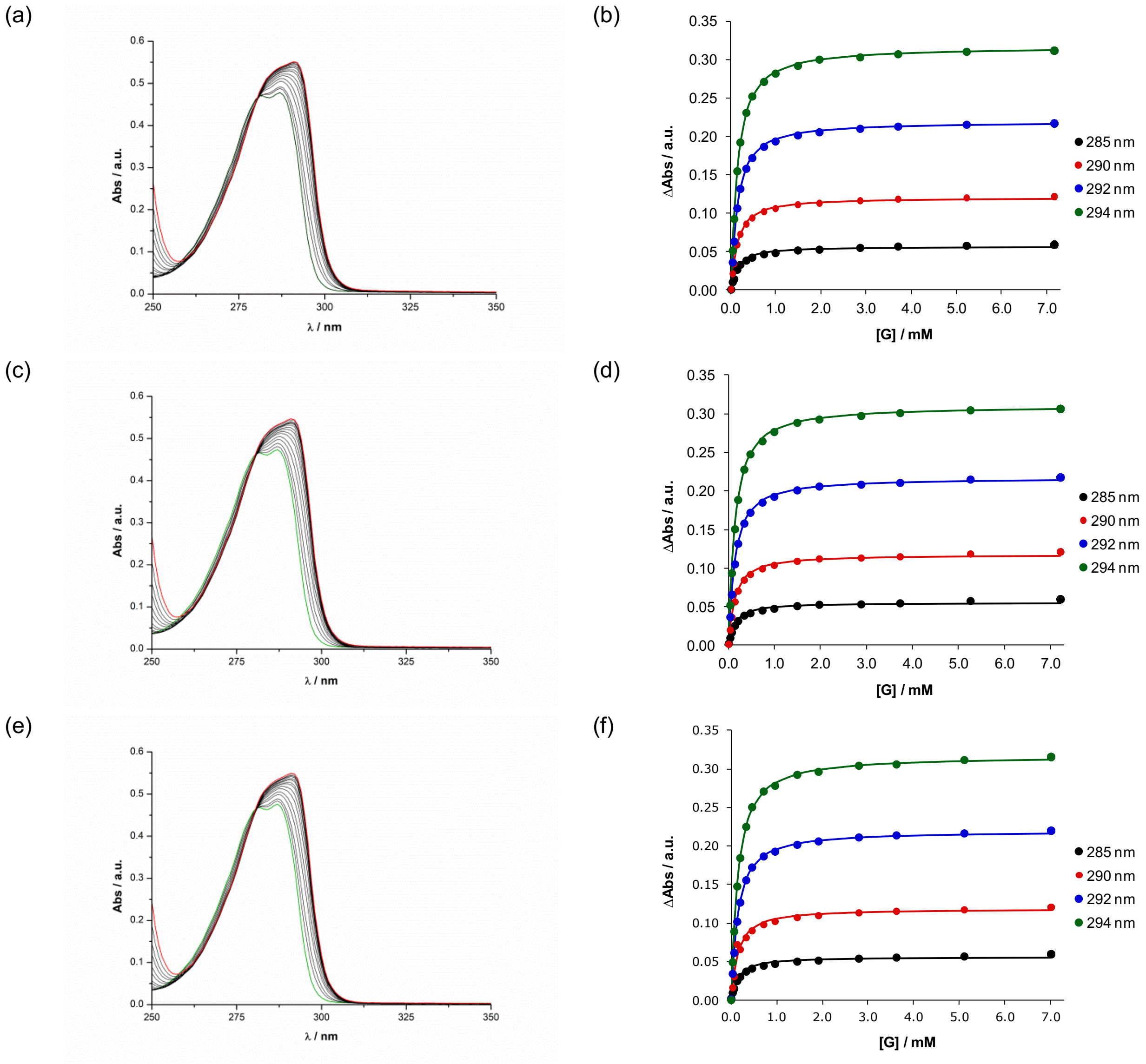

Figure S50. (a, c, e) UV-Vis spectra of **2** (0.075 mM) in *n*-octane at increasing concentrations of quinuclidine (from green to red), and (b, d, f) plot of the absorbance of **2** at 285 nm, 290 nm, 292 nm, 294 nm versus the concentration of **2** and its fittings to a 1:1 binding model.

Titrations of 2 with *n*-heptylamine in *n*-octane

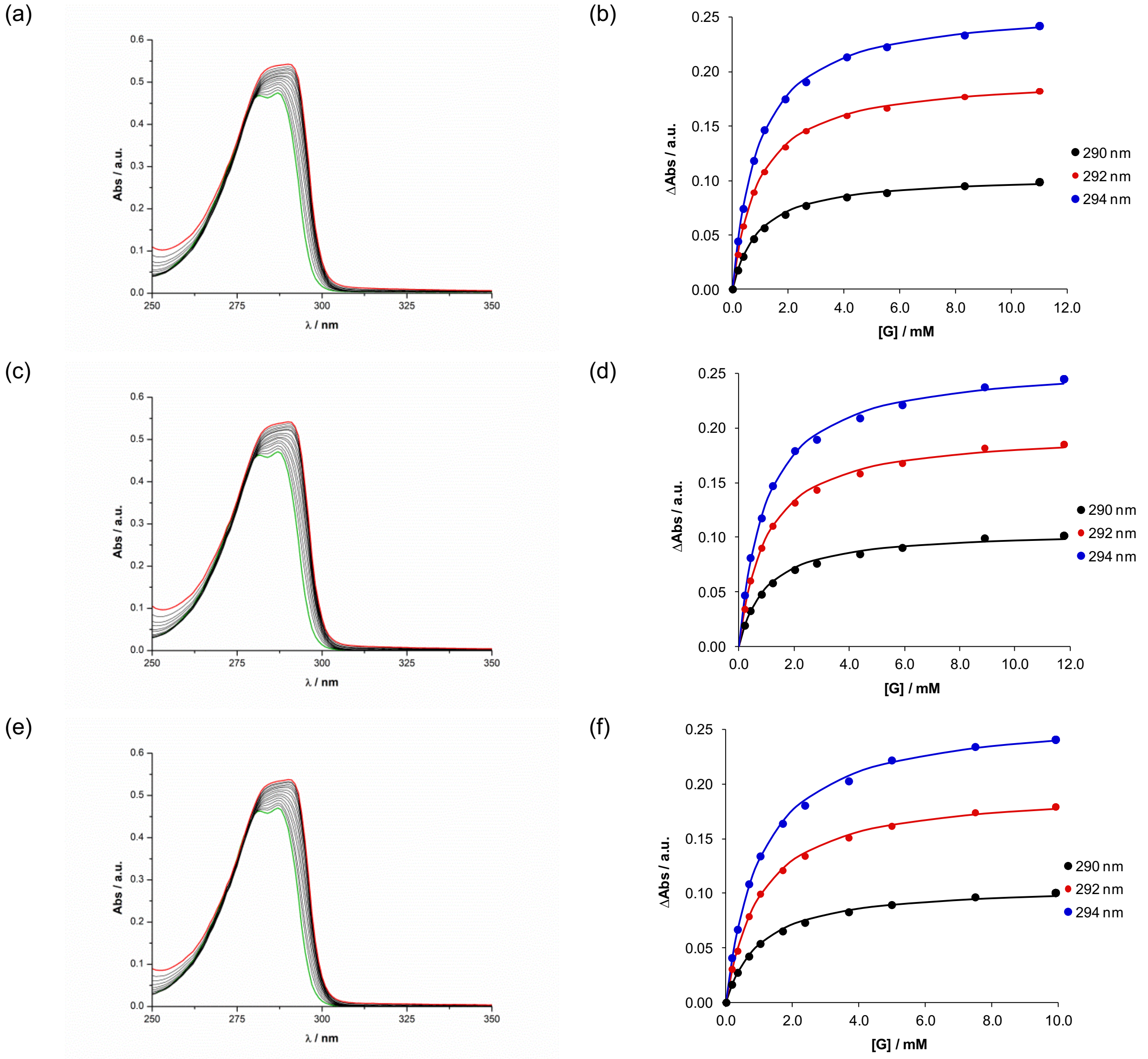

Figure S51. (a, c, e) UV-Vis spectra of **2** (0.075 mM) in *n*-octane at increasing concentrations of *n*-heptylamine (from green to red), and (b, d, f) plot of the absorbance of **2** at 290 nm, 292 nm, 294 nm versus the concentration of *n*-heptylamine and its fittings to a 1:1 binding model.

Titrations of 2 with tri-*n*-octylamine in *n*-octane

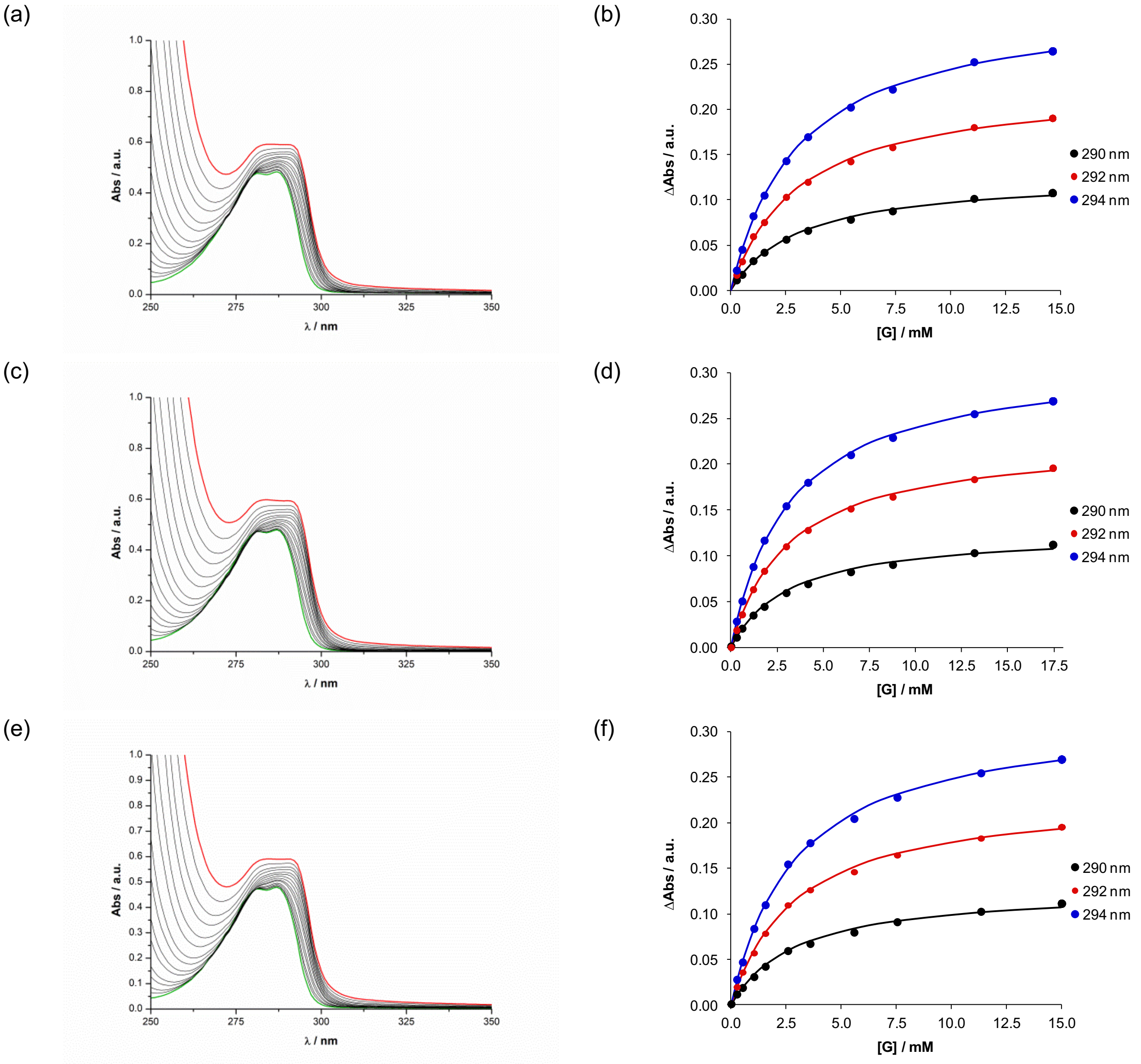

Figure S52. (a, c, e) UV-Vis spectra of **2** (0.075 mM) in *n*-octane at increasing concentrations of tri-*n*-octylamine (from green to red), and (b, d, f) plot of the absorbance of **2** at 290 nm, 292 nm, 294 nm versus the concentration of tri-*n*-octylamine and its fittings to a 1:1 binding model.

Molecule 3

Dilution experiment of 3 in *n*-octane

(a)

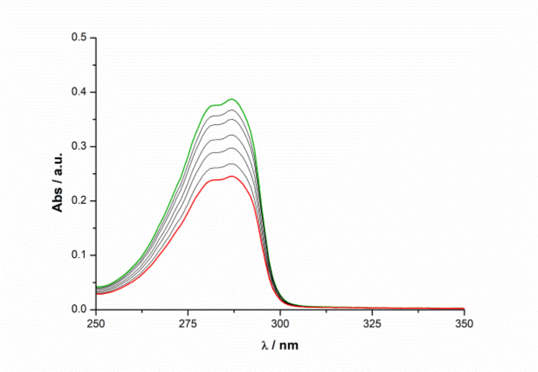

(b)

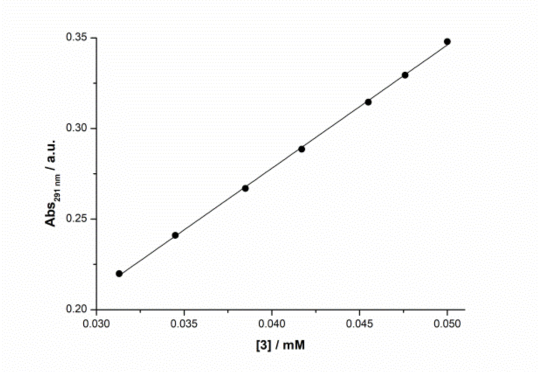

Figure S53. (a) UV-Vis spectra of **3** in *n*-octane at decreasing concentrations (from 0.050 mM in green to 0.031 mM in red), and (b) plot of the absorbance of **3** at 280 nm versus the concentration of **3** and its linear fitting.

Titrations of 3 with quinuclidine in *n*-octane

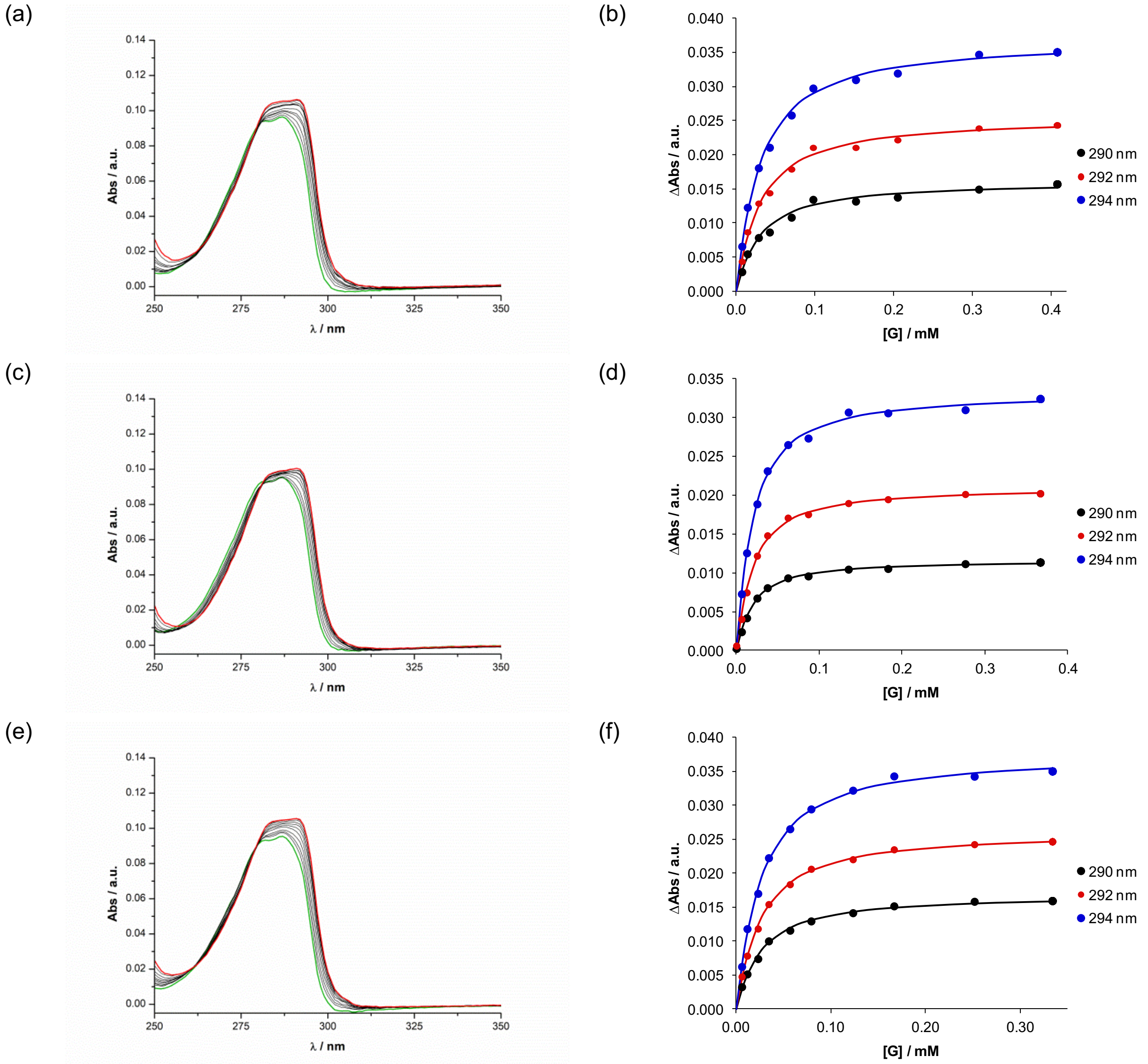

Figure S54. (a, c, e) UV-Vis spectra of **3** (0.010 mM for (a), 0.015 mM for (b) and (c)) in *n*-octane at increasing concentrations of quinuclidine (from green to red), and (b, d, f) plot of the absorbance of **3** at 280 nm versus the concentration of **3** and its fittings to a 1:1 binding model.

Titrations of 3 with *n*-heptylamine in *n*-octane

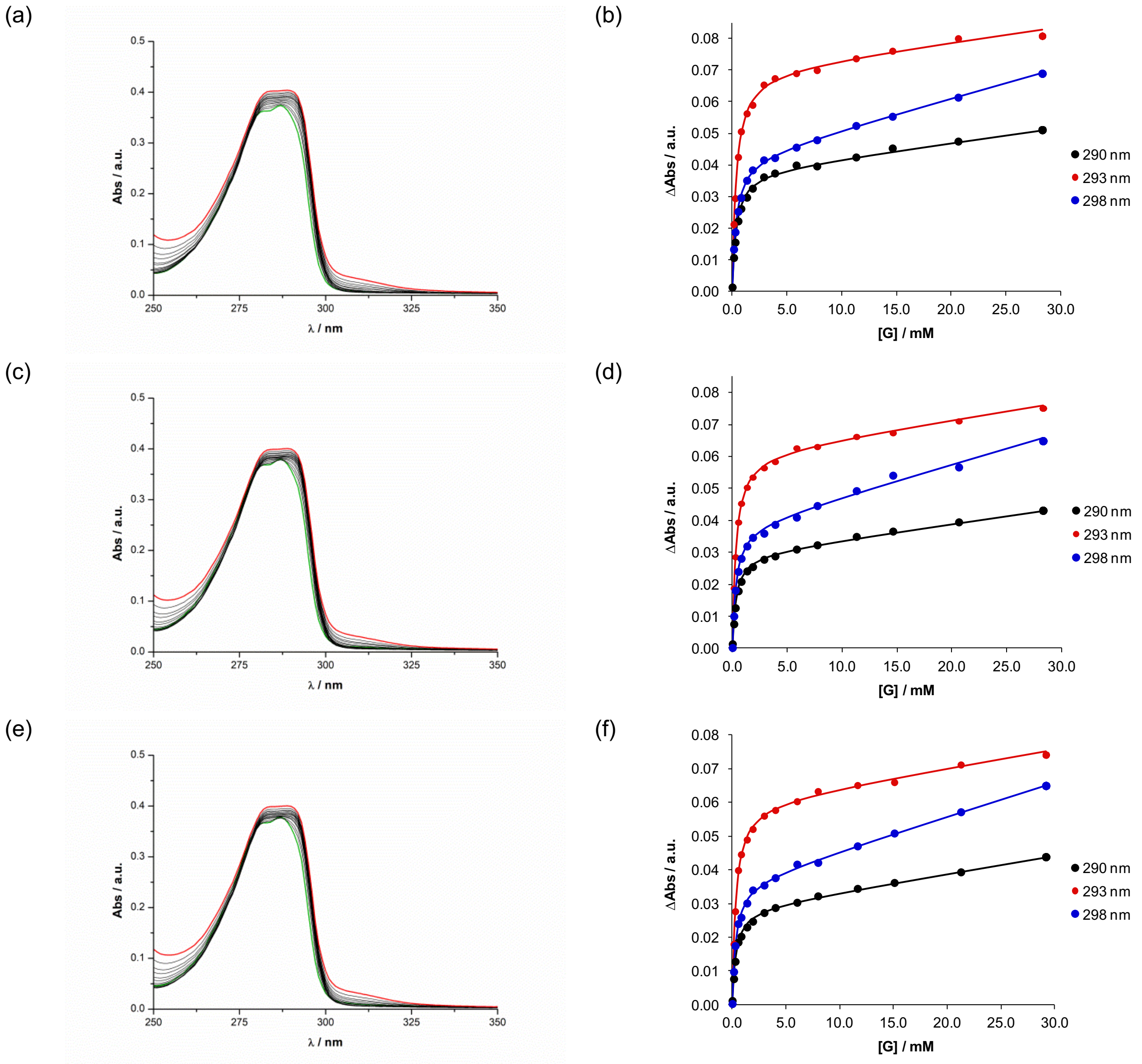

Figure S55. (a, c, e) UV-Vis spectra of **3** (0.050 mM) in *n*-octane at increasing concentrations of *n*-heptylamine (from green to red), and (b, d, f) plot of the absorbance of **3** at 290 nm, 293 nm, 298 nm versus the concentration of *n*-heptylamine and its fittings to a 1:1+non-specific binding model.

Titrations of 3 with tri-*n*-octylamine in *n*-octane

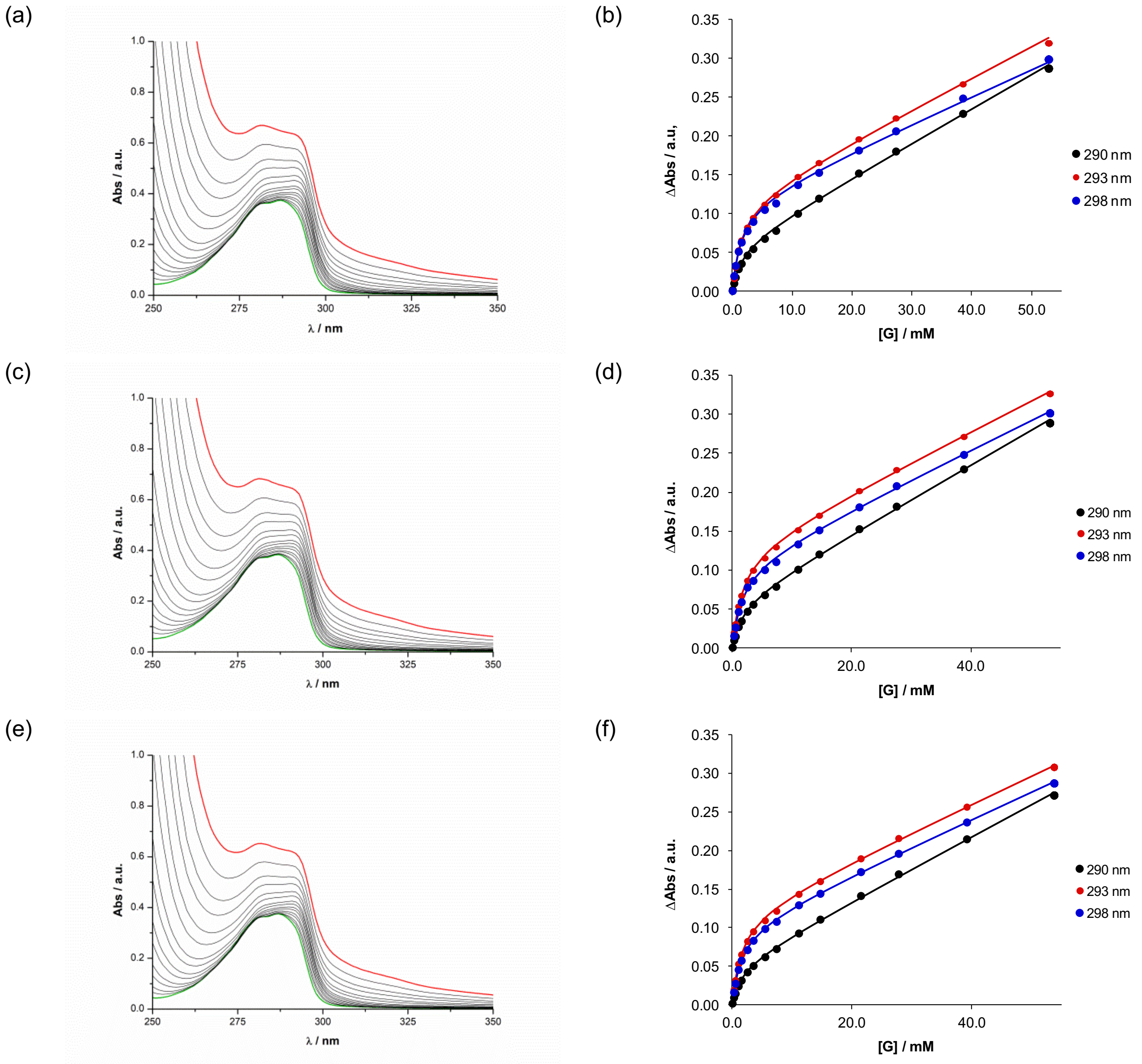

Figure S56. (a, c, e) UV-Vis spectra of **3** (0.050 mM) in *n*-octane at increasing concentrations of tri-*n*-octyalmine (from green to red), and (b, d, f) plot of the absorbance of **3** at 290 nm, 293 nm, 298 nm versus the concentration of **3** and its fittings to a 1:1+non-specific binding model.

Molecule 4
Dilution Experiment of 4 in *n*-octane

(a)

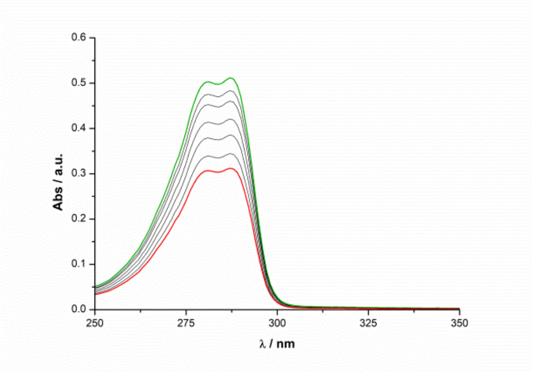

(b)

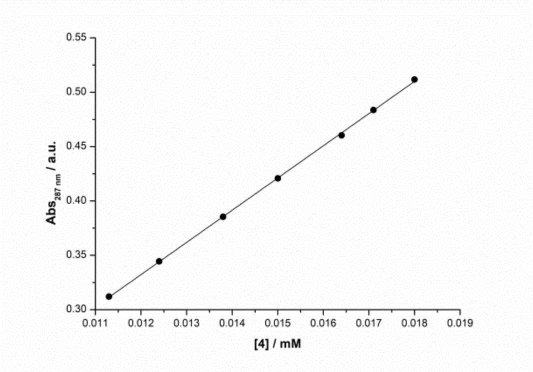

Figure S57. (a) UV-Vis spectra of **4** in *n*-octane at decreasing concentrations (from 0.018 mM in green to 0.011 mM in red), and (b) plot of the absorbance of **4** at 287 nm versus the concentration of **4** and its linear fitting.

Titrations of 4 with quinuclidine in *n*-octane

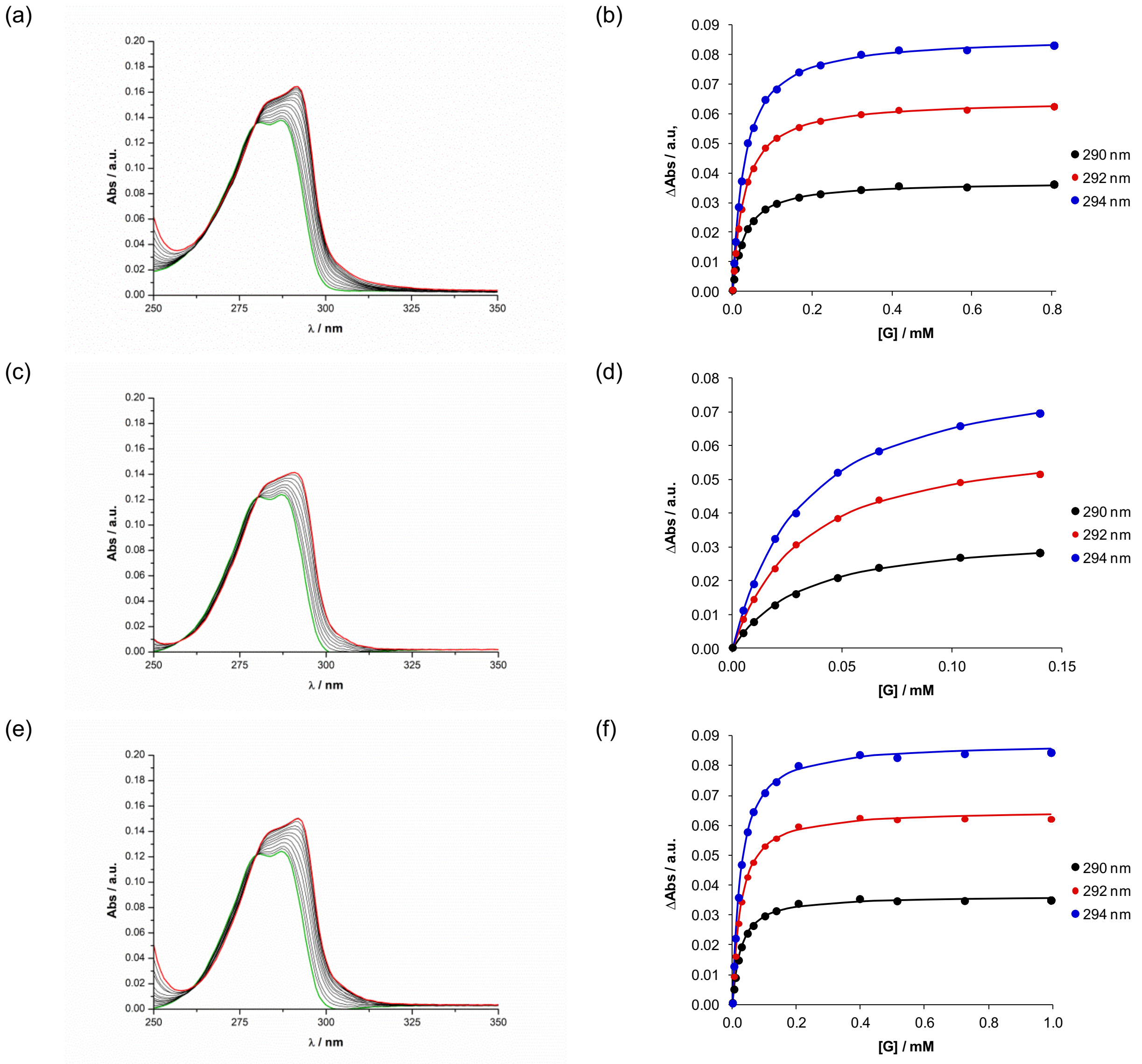

Figure S58. (a, c, e) UV-Vis spectra of **4** (0.005 mM) in *n*-octane at increasing concentrations of quinuclidine (from green to red), and (b, d, f) plot of the absorbance of **4** at 290 nm, 292 nm, 294 nm versus the concentration of **4** and its fittings to a 1:1 binding model.

Titrations of 4 with *n*-heptylamine in *n*-octane

(a)

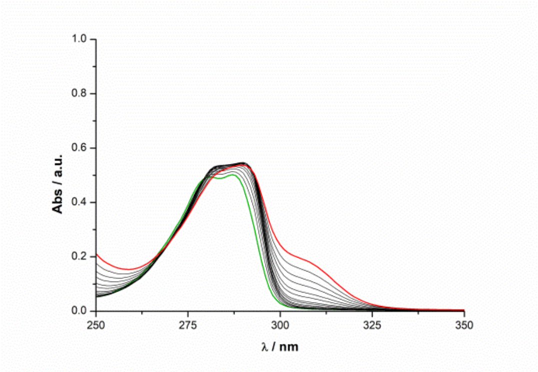

(b)

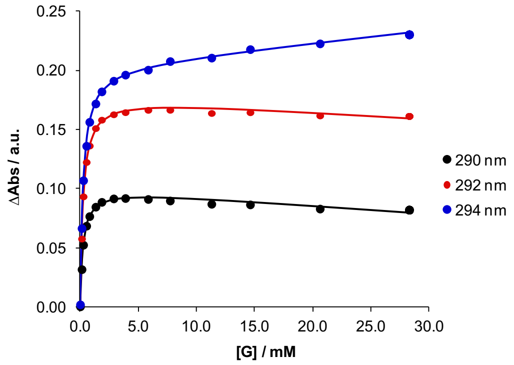

(c)

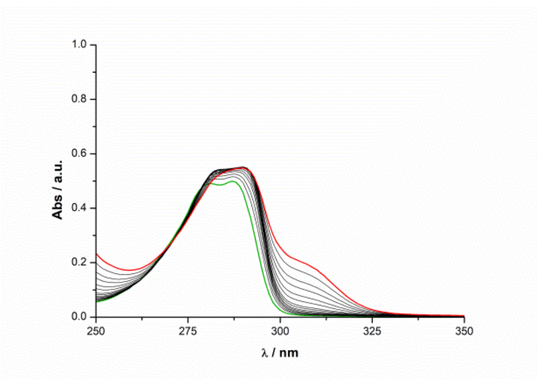

(d)

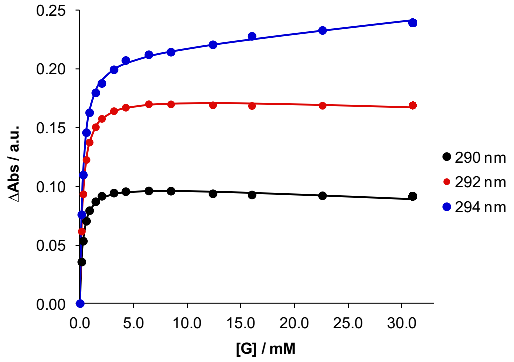

(e)

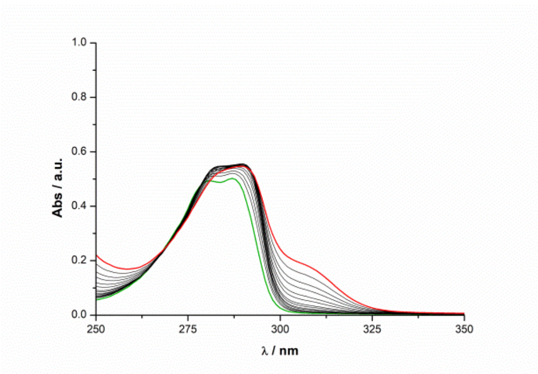

(f)

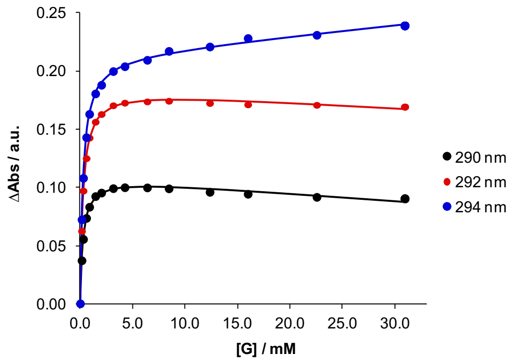

Figure S59. (a, c, e) UV-Vis spectra of **4** (0.018 mM) in *n*-octane at increasing concentrations of *n*-heptylamine (from green to red), and (b, d, f) plot of the absorbance of **4** at 290 nm, 292 nm, 294 nm versus the concentration of **4** and its fittings to a 1:1+non-specific binding model.

Titrations of 4 with tri-*n*-octylamine in *n*-octane

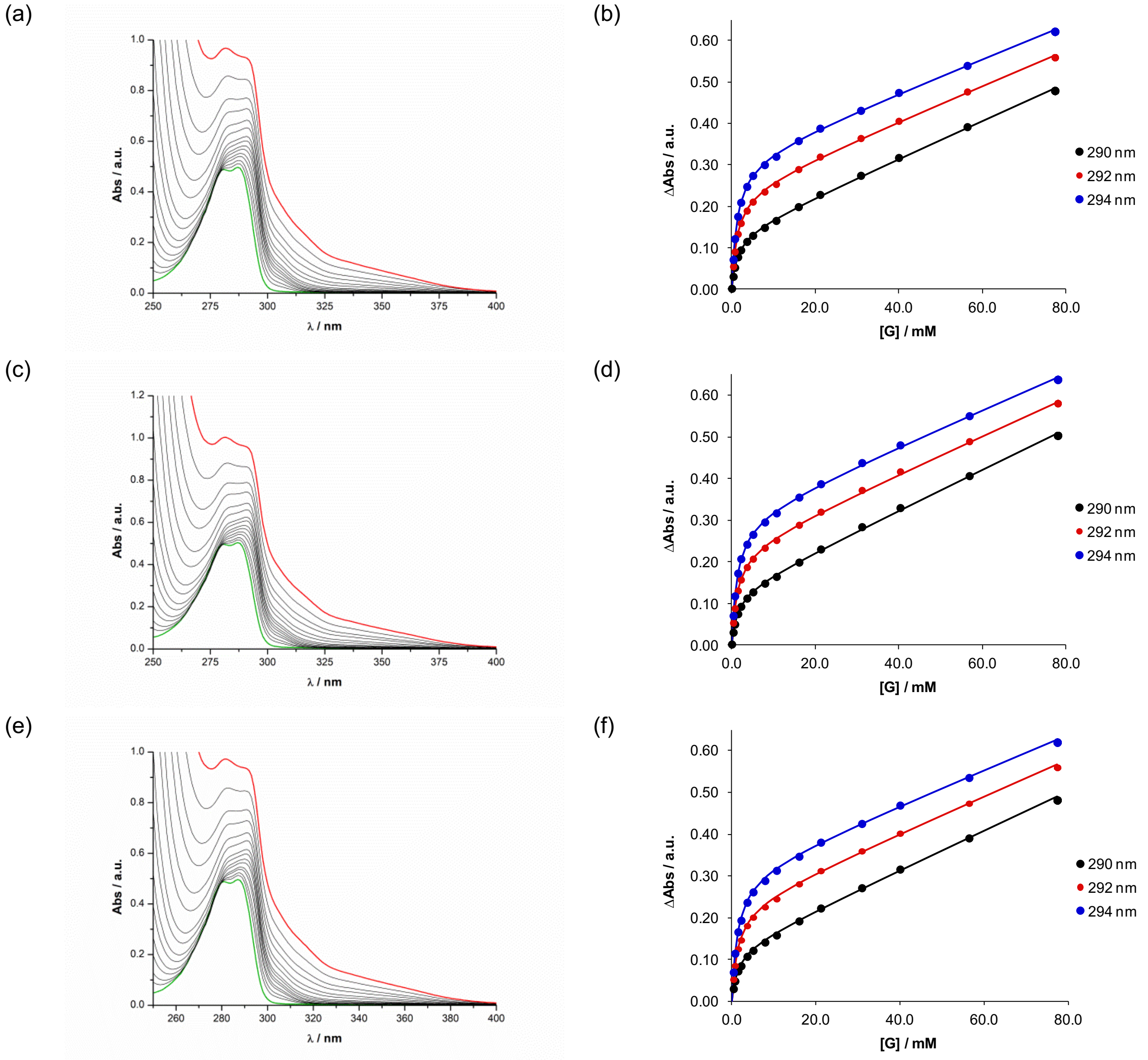

Figure S60. (a, c, e) UV-Vis spectra of **4** (0.018 mM) in *n*-octane at increasing concentrations of tri-*n*-octylamine (from green to red), and (b, d, f) plot of the absorbance of **4** at 290 nm, 292 nm, 294 nm versus the concentration of **4** and its fittings to a 1:1+non-specific binding model.

Molecule 5

Dilution Experiment of 5 in *n*-octane

(a) 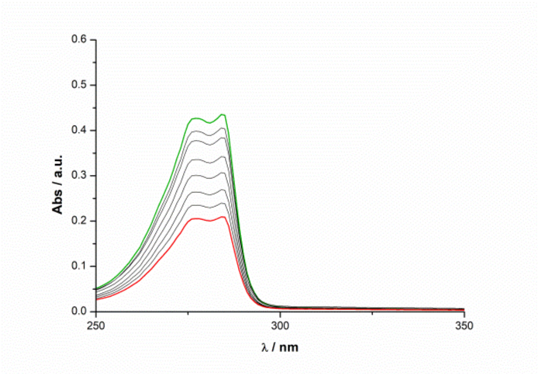

(b) 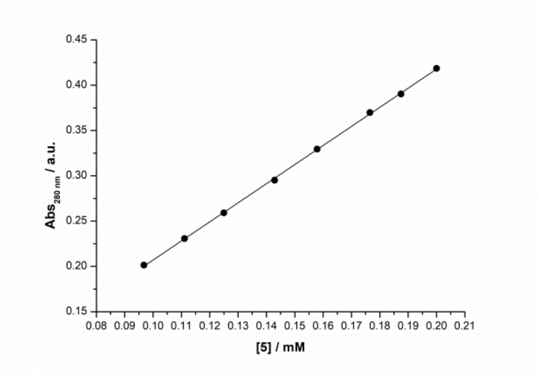

Figure S61. (a) UV-Vis spectra of **5** in *n*-octane at decreasing concentrations (from 0.200 mM in green to 0.111 mM in red), and (b) plot of the absorbance of **5** at 280 nm versus the concentration of **5** and its linear fitting.

Titrations of 5 with quinuclidine in *n*-octane

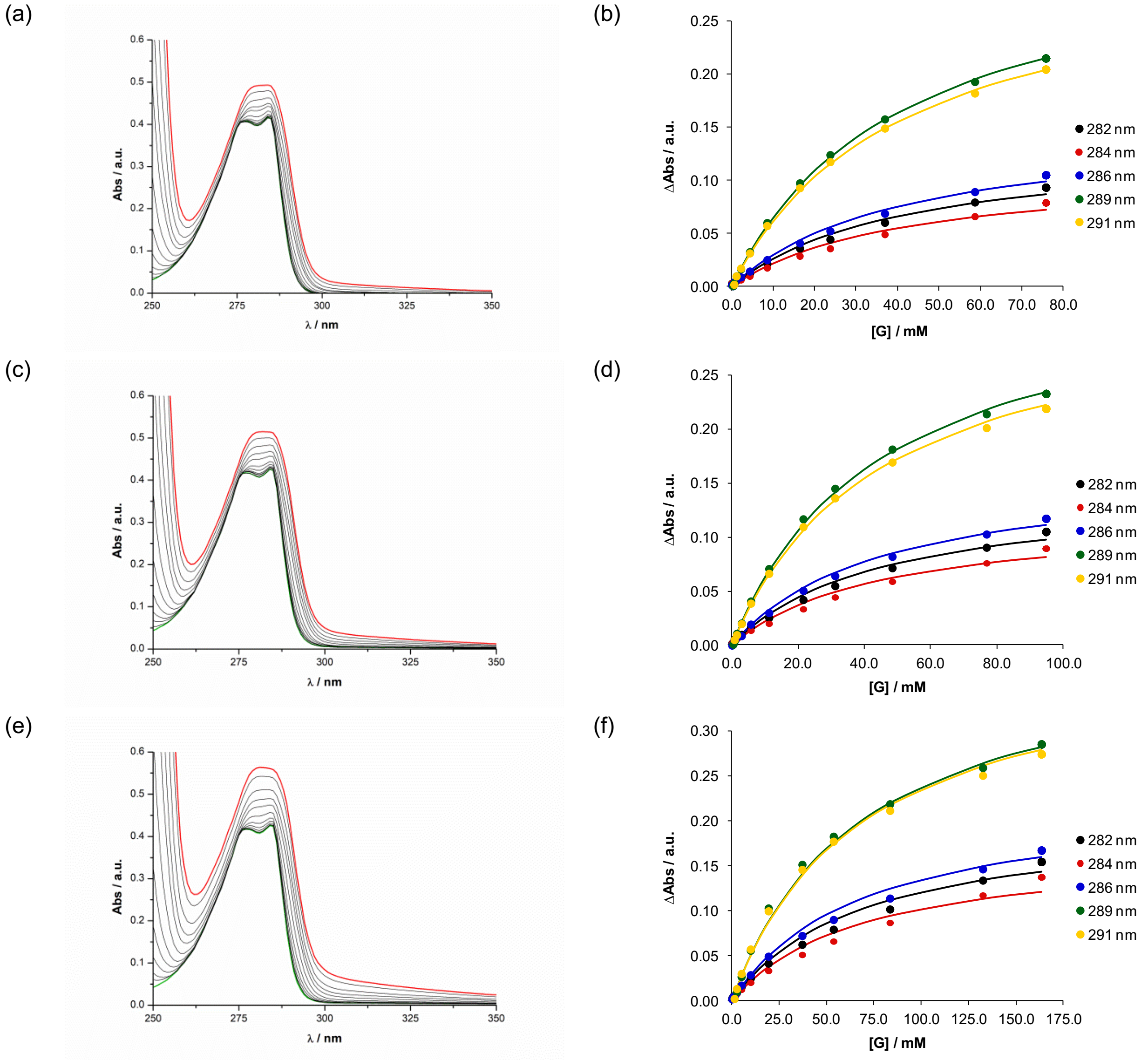

Figure S62. (a, c, e) UV-Vis spectra of **5** (0.200 mM) in *n*-octane at increasing concentrations of quinuclidine (from green to red), and (b, d, f) plot of the absorbance of **5** at 282 nm, 284 nm, 286 nm, 289 nm, 291 nm versus the concentration of **5** and its fittings to a 1:1 binding model.

Titrations of 5 with *n*-heptylamine in *n*-octane

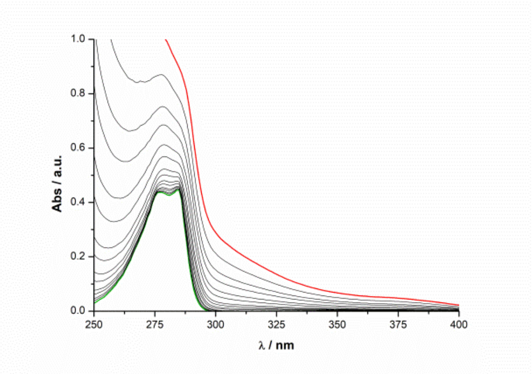

Figure S63. (a, c, e) UV-Vis spectra of **5** (0.200 mM) in *n*-octane at increasing concentrations of *n*-heptylamine (from green to red).

Titration of 5 with tri-*n*-octylamine in *n*-octane

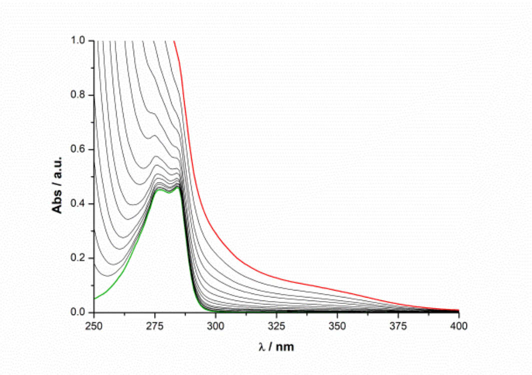

Figure S64. UV-Vis spectra of **5** (0.200 mM) in *n*-octane at increasing concentrations of tri-*n*-octylamine (from green to red).

Molecule 6

Dilution experiment of 6 in *n*-octane

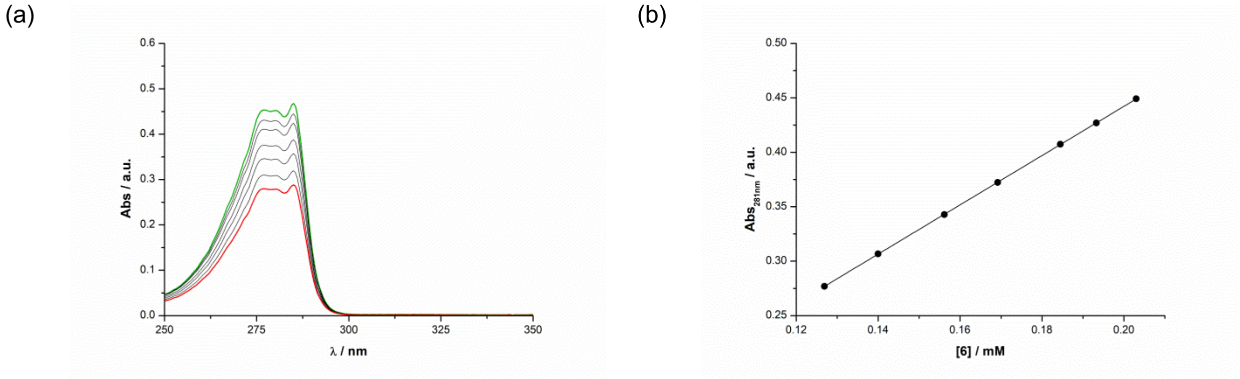

Figure S65. (a) UV-Vis spectra of **6** in *n*-octane at decreasing concentrations (from 0.203 mM in green to 0.127 mM in red), and (b) plot of the absorbance of **6** at 281 nm versus the concentration of **6** and its linear fitting.

Titrations of 6 with quinuclidine in *n*-octane

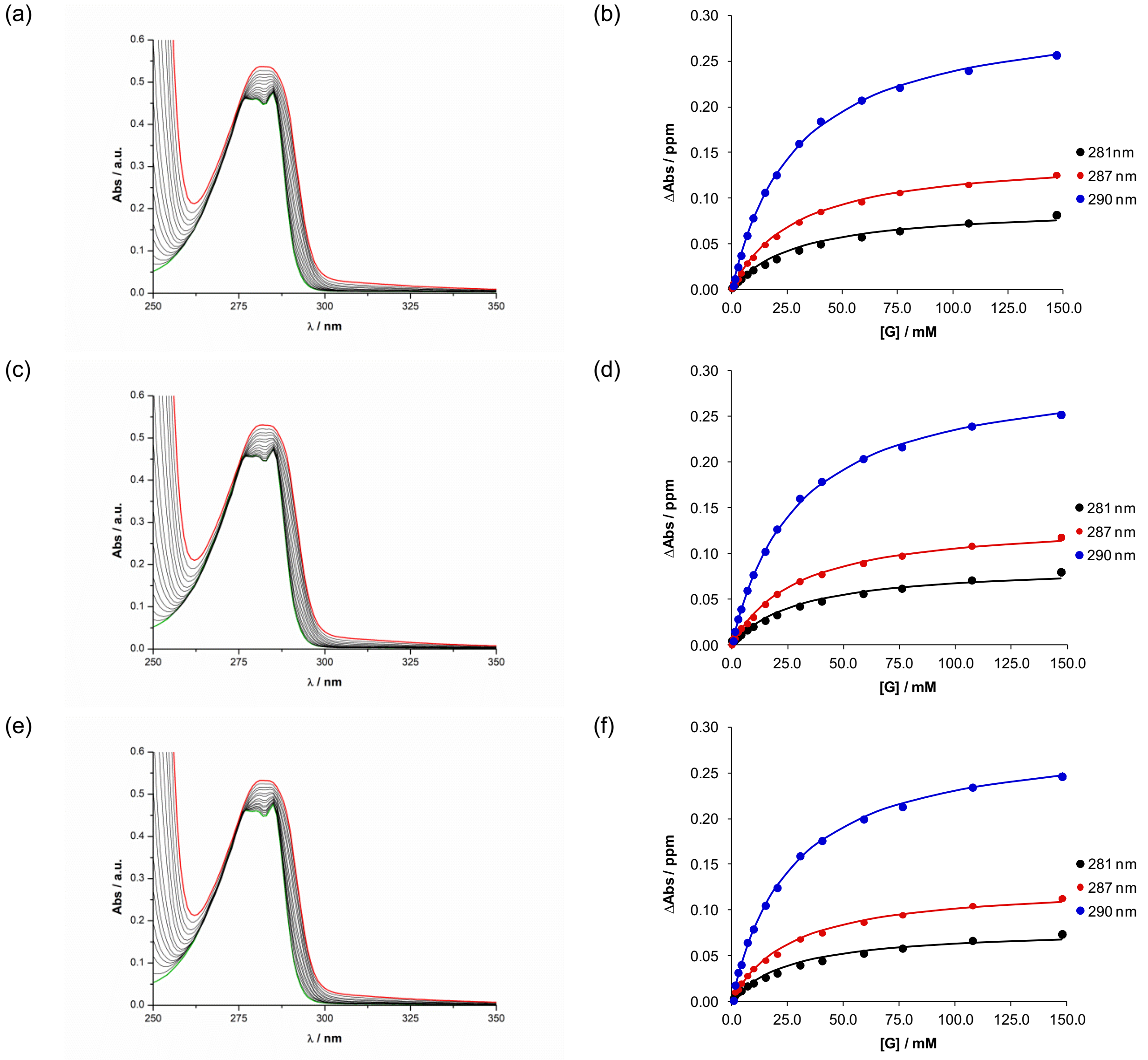

Figure S66. (a, c, e) UV-Vis spectra of **6** (0.203 mM) in *n*-octane at increasing concentrations of quinuclidine (from green to red), and (b, d, f) plot of the absorbance of **6** at 281 nm, 287 nm, 290 nm versus the concentration of **6** and its fittings to a 1:1 binding model.

Titrations of 6 with *n*-heptylamine in *n*-octane

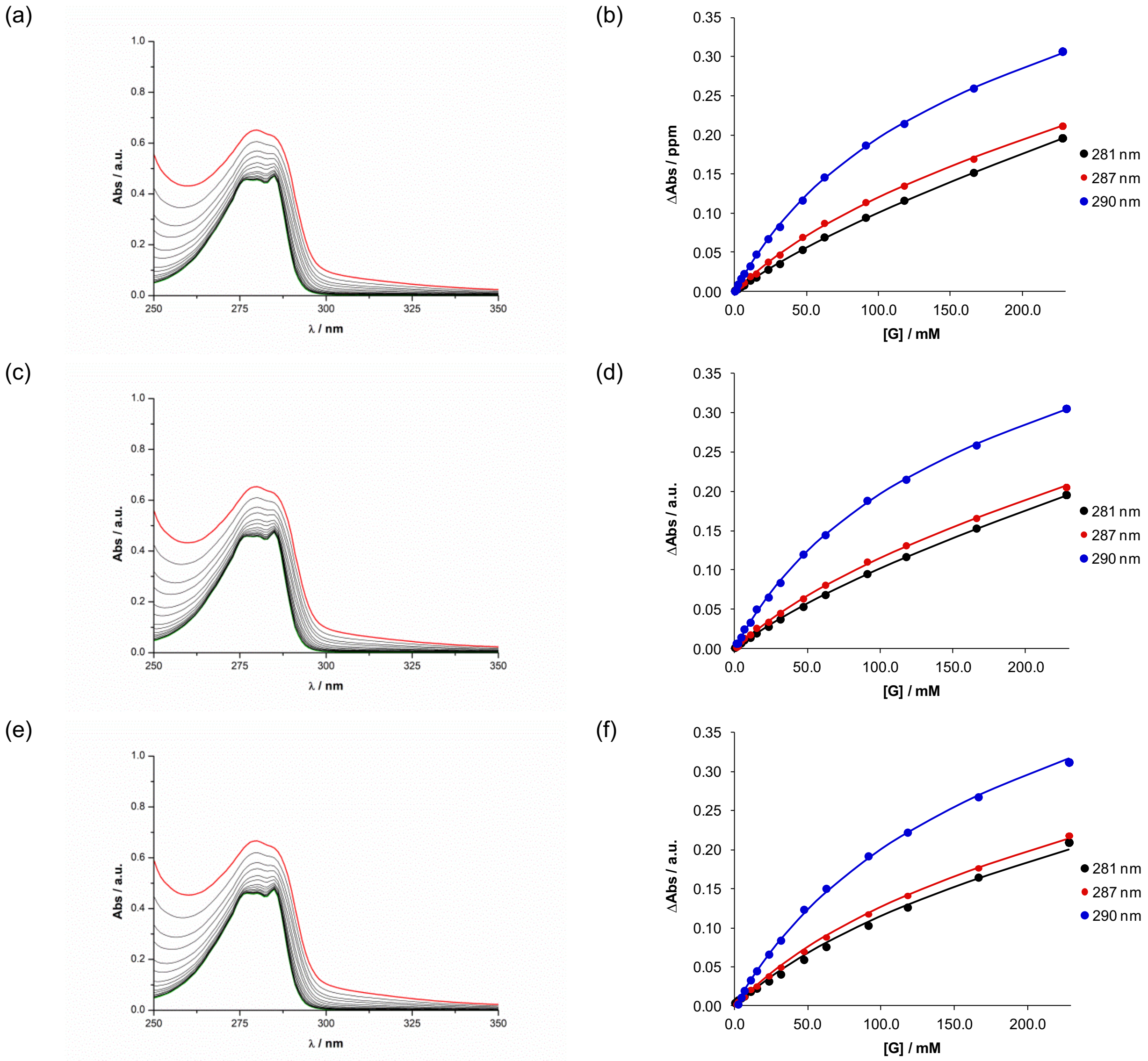

Figure S67. (a, c, e) UV-Vis spectra of **6** (0.203 mM) in *n*-octane at increasing concentrations of *n*-heptylamine (from green to red), and (b, d, f) plot of the absorbance of **6** at 281 nm, 287 nm, 290 nm versus the concentration of **6** and its fittings to a 1:1+non-specific binding model.

Titrations of 6 with tri-*n*-octylamine in *n*-octane

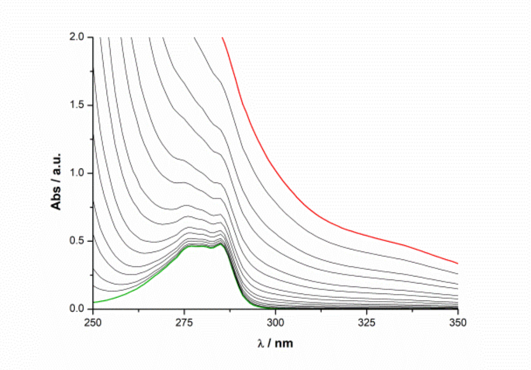

Figure S68. (a, c, e) UV-Vis spectra of **6** (0.203 mM) in *n*-octane at increasing concentrations of tri-*n*-octylamine (from green to red).

Details of computational studies

Molecular mechanics calculations were done using Schrödinger's Maestro software (2019-1 edition), with $CHCl_3$ as the solvent and OPLS 2005 as the force field (charges assigned from the force field, the cut-off was none so that all non-bonded interactions are considered). The minimization method that was used was PRCG (Polak-Ribier Conjugate Gradient)[20] with a maximum iterations number of 10000, the convergence criterion was a gradient with a convergence threshold of 0.01 kJ mol^{-1} $Å^{-1}$. For each conformational search mixed torsional/low mode sampling was used as the method, the maximum number of steps was 10000, the number of structures saved for each search was 50 and the energy window for saving structures was 50.0 kJ/mol.

Starting from the structure with the lowest energy and the desired conformation (i.e. with the intramolecular H-bonds present) calculated with molecular mechanics, all the molecules were footprinted as described previously.[21]

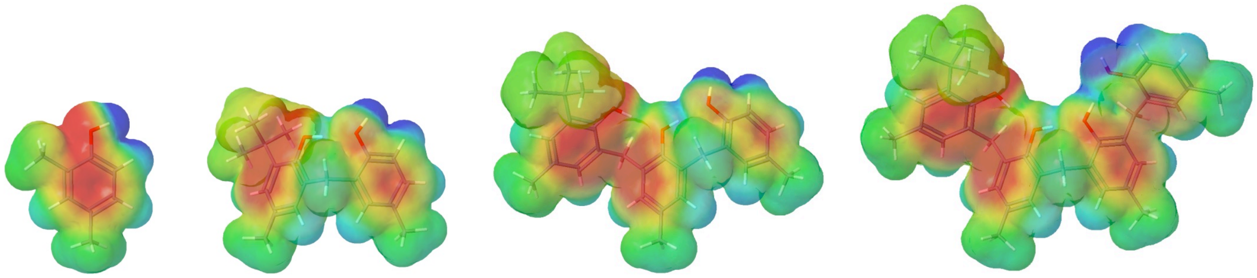

1 **2** **3** **4**

Figure S69. Molecular electrostatic potential surface (MEPS) of **1**, **2**, **3** and **4**.

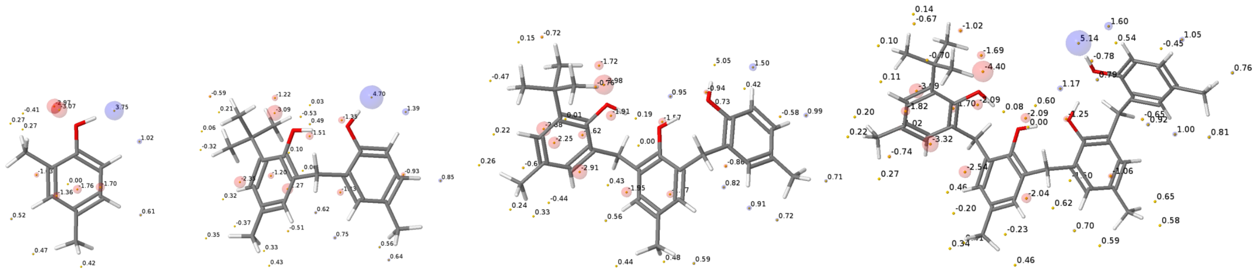

1 **2** **3** **4**

Figure S70. Surface site interaction points (SSIPs) for **1**, **2**, **3** and **4**.

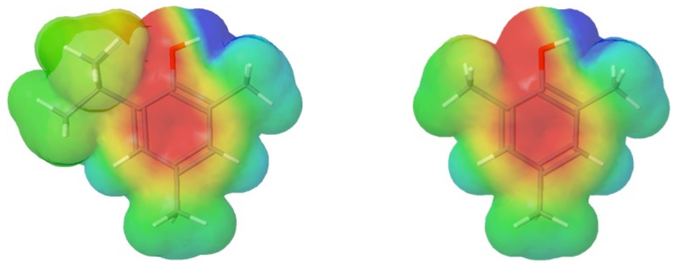

5 **6**

Figure S71. Molecular electrostatic potential surface (MEPS) of **5** and **6**.

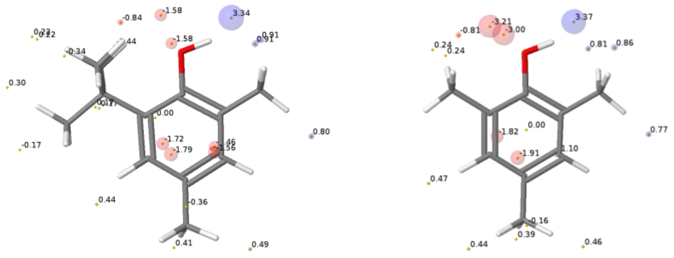

5 6

Figure S72. Surface site interaction points (SSIPs) for **5** and **6**.

	Calculated	Experiment		
**Donor**		**Acceptor**		
		**Quin**	**HeptNH$_2$**	**Oct$_3$N**
**1**	3.7	3.5	3.5	-
**2**	4.7	4.6	4.6	4.5
**3**	5.0	5.1	5.0	4.8
**4**	5.1	5.0	5.1	4.8
**5**	3.3	2.8	-	-
**6**	3.4	3.0	2.9	-

Table S3. H-bond donor parameters (α).

The geometry of the structures of **1-4** with the lowest energy and the desired conformation (i.e. with the intramolecular H-bonds present) calculated with molecular mechanics were optimised with Jaguar (functional MO6-2X, basis set 6-31G**, gas phase) followed by a single point energy calculation to compute the values of the Mulliken atomic charges for the terminal hydroxyl protons (Table S4).

Donor	Mulliken atomic charges
**1**	0.33852
**2**	0.35161
**3**	0.35438
**4**	0.35662

Table S4. Mulliken atomic charges of the terminal hydroxyl protons of **1-4**.

References

1. Fulmer, G. R.; Miller, A. J.; Sherden, N. H.; Gottlieb, H. E.; Mudelman, A.; Stoltz, B. M.; Bercaw, J. E.; Goldberg, K. I. NMR Chemical Shifts of Trace Impurities: Common Laboratory Solvents, Organics, and Gases in Deuterated Solvents Relevant to the Organometallic Chemist. *Organometallics* **2010**, *29* (9), 2176-2179. DOI: 10.1021/om100106e
2. Sartori, G.; Bigi, F.; Maggi, R.; Porta, C. Metal-template *ortho*-Regioselective Mono and Bis-de-*tert*-butylation of Poly-*tert*-butylated Phenols. *Tetrahedron Letters* **1994**, *35* (38), 7073-7076. DOI: 10.1016/0040-4039(94)88229-0
3. Katritzky, A. R.; Zhang, Z.; Lang, H.; Lan, X. *o*-(α-Benzotriazolylalkyl)phenols: Novel Precursors for the Preparation of 1,1-Bis(2-hydroxyaryl)alkanes. *J. Org. Chem.* **1994**, *59* (24), 7209-7213. DOI: 10.1021/jo00103a009
4. Ingenfeld, B.; Straub, S.; Frömbgen, C.; Lützen, A. Synthesis of Monofunctionalized Calix[5]arenes. *Synthesis* **2018**, *50*, 676-684. DOI: 10.1055/s-0036-1589127
5. Bown, D. E. Poly alpha Monoolefins Stabilized with Alkylated Trisphenolic Ether. US3109829 (A), 1963.
6. Böhmer, V.; Beismann, K.; Stotz, D.; Niemann, W.; Vogt, W. Kinetics of the Bromination of Phenols and Oligonuclear Phenolic Compounds, 6. Far reaching effects via chains of intramolecular hydrogen bonds. *Makromol. Chem.* **1983**, *184*, 1793–1806. DOI: 10.1002/macp.1983.021840904
7. Moon, N. G.; Harned A. M. A Concise Synthetic Route to the Stereotetrad Core of the Briarane Diterpenoids. *Org. Lett.* **2015**, *17*, 2218-2221. DOI: 10.1021/acs.orglett.5b00816.
8. Stasiw, D. E.; Luke, A. M.; Rosen, T.; League, A. B.; Mandal, M.; Neisen, B. D.; Cramer, C. J.; Kol, M.; Tolman, W. B. Mechanism of the Polymerization of *rac*-Lactide by Fast Zinc Alkoxide Catalysts. *Inorg. Chem.* **2017**, *56*, 14366-14372. DOI: 10.1021/acs.inorgchem.7b02544
9. Sheldrick, G. M. SHELXT – Integrated Space-group and Crystal-structure Determination. *Acta Cryst. Sect. A* **2015**, *71*, 3-8. DOI: 10.1107/S2053273314026370
10. Sheldrick, G. M. Crystal Structure Refinement with SHELXL. *Acta Cryst. Sect. C*, **2015**, *71*, 3-8. DOI: 10.1107/S2053229614024218
11. Clark, S. J.; Segall, M.D.; Pickard, C. J.; Hasnip, P. J.; Probert, M. I. J.; Refson, K.; Payne M. C. First Principle Methods Using CASTEP. *Z. Kristallogr.* **2005**, *220*, 567-570. DOI: 10.1524/zkri.220.5.567.65075
12. Perdew, J. P.; Burke, K.; Ernzerhof, M. Generalized Gradient Approximation Made Simple. *Phys. Rev. Lett.* **1996**, *77*, 3865-3868. DOI: 10.1103/PhysRevLett.77.3865
13. Grimme, S. Semiempirical GGA-type Density Functional Constructed with a Long-range Dispersion Correction. *J. Comput. Chem.* **2006**, *27* (15), 1787-1799. DOI: 10.1002/jcc.20495
14. Paulus, E.; Böhmer, V. Die Kristallstruktur von Oligo[(2-hydroxy-1,3-phenylen)methylen]en. *Makromol. Chem.* **1984**, *185* (9), 1921-1935. DOI: 10.1002/macp.1984.021850914
15. Macrae, C. F.; Edgington, P. R.; McCabe, P.; Pidcock, E.; Shields, G. P.; Taylor, R.; Towler, M.; van de Streek, J. Mercury: Visualization and Analysis of Crystal Structures. *J. Appl. Cryst.* **2006**, *39*, 453-457. DOI: 10.1107/S002188980600731X
16. van de Streek, J.; Neumann, M. A. Validation of Experimental Molecular Crystal Structures with Dispersion-corrected Density Functional Theory Calculations. *Acta Cryst. Sect. B*, **2010**, *66*, 544-558. DOI: 10.1107/S0108768110031873
17. Anslyn, V. E.; Dougherty, D. A. *Modern Physical Organic Chemistry*; University Science Books, 2005, pp 216-221.

18. Jeener, J.; Meier, B. H.; Bachmann, P.; Ernst, R. R. Investigation of Exchange Processes by Two-dimensional NMR Spectroscopy. *J. Chem. Phys.* **1979**, *71* (11), 4546-4553. DOI: 10.1063/1.438208
19. Berger, S. Gradient-selected NOESY – A Fourfold Reduction of the Measurement Time for the NOESY Experiment. *J. Magn. Reson. Ser. A* **1996**, *123*, 119-121. DOI: 10.1006/jmra.1996.0222
20. Polak, E.; Ribiere G. Note sur la Convergence de Méthodes de Directions Conjuguées. Revue Française d'Informatique et de Recherche Opérationelle. Série rouge, **1969**, *16*, 35-43.
21. Calero, C. S.; Farwer, J.; Gardiner, E. J.; Hunter, C. A.; Mackey, M.; Scuderi, S.; Thompson, S.; Vinter, J. G. Footprinting Molecular Electrostatic Potential Surfaces for Calculation of Solvation Energies. *Phys. Chem. Chem. Phys.* **2013**, *15*, 18262–73. DOI: 10.1039/C3CP53158A
